# ETfinder: harnessing conserved C-terminal tails of single-stranded DNA-binding proteins for mining and engineering RecET systems in non-model microbial chassis

**DOI:** 10.1093/nar/gkag818

**Published:** 2026-08-20

**Authors:** Dongyuan Lv, Mindong Liang, Yiyang Gu, Xiangying Zhu, Hailong Wang, Menglei Xia, Jinkang Hao, Youyuan Li, Weishan Wang, Linquan Bai, Jiagao Cheng, Lixin Zhang, Gao-Yi Tan

**Affiliations:** State Key Laboratory of Bioreactor Engineering (SKLBE) and School of Biotechnology, East China University of Science and Technology (ECUST), Shanghai 200237, China; State Key Laboratory of Bioreactor Engineering (SKLBE) and School of Biotechnology, East China University of Science and Technology (ECUST), Shanghai 200237, China; State Key Laboratory of Bioreactor Engineering (SKLBE) and School of Biotechnology, East China University of Science and Technology (ECUST), Shanghai 200237, China; Shanghai Key Laboratory of Chemical Biology, School of Pharmacy, East China University of Science and Technology, Shanghai 200237, China; State Key Laboratory of Microbial Technology, School of Life Science, Shandong University, Jinan 250100, China; Metabolism and Fermentation Process Control, College of Biotechnology, Ministry of Education, Tianjin University of Science and Technology, Tianjin 300457, China; Metabolism and Fermentation Process Control, College of Biotechnology, Ministry of Education, Tianjin University of Science and Technology, Tianjin 300457, China; State Key Laboratory of Bioreactor Engineering (SKLBE) and School of Biotechnology, East China University of Science and Technology (ECUST), Shanghai 200237, China; State Key Laboratory of Microbial Resources, Institute of Microbiology, Chinese Academy of Sciences, Beijing 100101, China; State Key Laboratory of Microbial Metabolism and School of Life Sciences & Biotechnology, Shanghai Jiao Tong University, Shanghai 200240, China; State Key Laboratory of Bioreactor Engineering (SKLBE) and School of Biotechnology, East China University of Science and Technology (ECUST), Shanghai 200237, China; Shanghai Key Laboratory of Chemical Biology, School of Pharmacy, East China University of Science and Technology, Shanghai 200237, China; State Key Laboratory of Bioreactor Engineering (SKLBE) and School of Biotechnology, East China University of Science and Technology (ECUST), Shanghai 200237, China; State Key Laboratory of Bioreactor Engineering (SKLBE) and School of Biotechnology, East China University of Science and Technology (ECUST), Shanghai 200237, China

## Abstract

Nonmodel microorganisms offer substantial potential as next-generation microbial chassis (NGMCs), yet most lack efficient and broadly transferable genome-editing systems. Here we present ETfinder, a framework that uses the conserved C-terminal tail of host single-stranded DNA-binding proteins (SSB-Ct) as a biochemical constraint to guide the discovery of RecET recombineering systems. Applied to *Rhodobacter sphaeroides*, ETfinder identified 91 candidates from 18 841 α-proteobacterial genomes, and all five experimentally tested RecT homologs supported measurable double-stranded DNA (dsDNA) recombineering, with the *Paracoccaceae* SJ630 system reaching 8.9 × 10² colony-forming units (CFU) per μg of dsDNA and 100% editing accuracy. Testing in *Halomonas* further showed that RecT proteins from evolutionarily distant taxa remain functional within the same halophilic chassis, indicating that SSB-Ct-guided selection enriches for portable recombination modules beyond phylogenetic proximity. To facilitate broad adoption, we compiled 25 529 RecT–SSB pairs into a curated database and implemented ETfinder as a standalone, locally deployable toolkit for mining, ranking, and phylogenetic visualization. This framework prioritizes high-compatibility homologs, reduces experimental screening burden, and expands the accessible genome-editing toolbox for NGMCs. ETfinder is freely available at https://github.com/lvdongyuan/ETfinder.

## Graphical Abstract

**Graphical Abstract:**
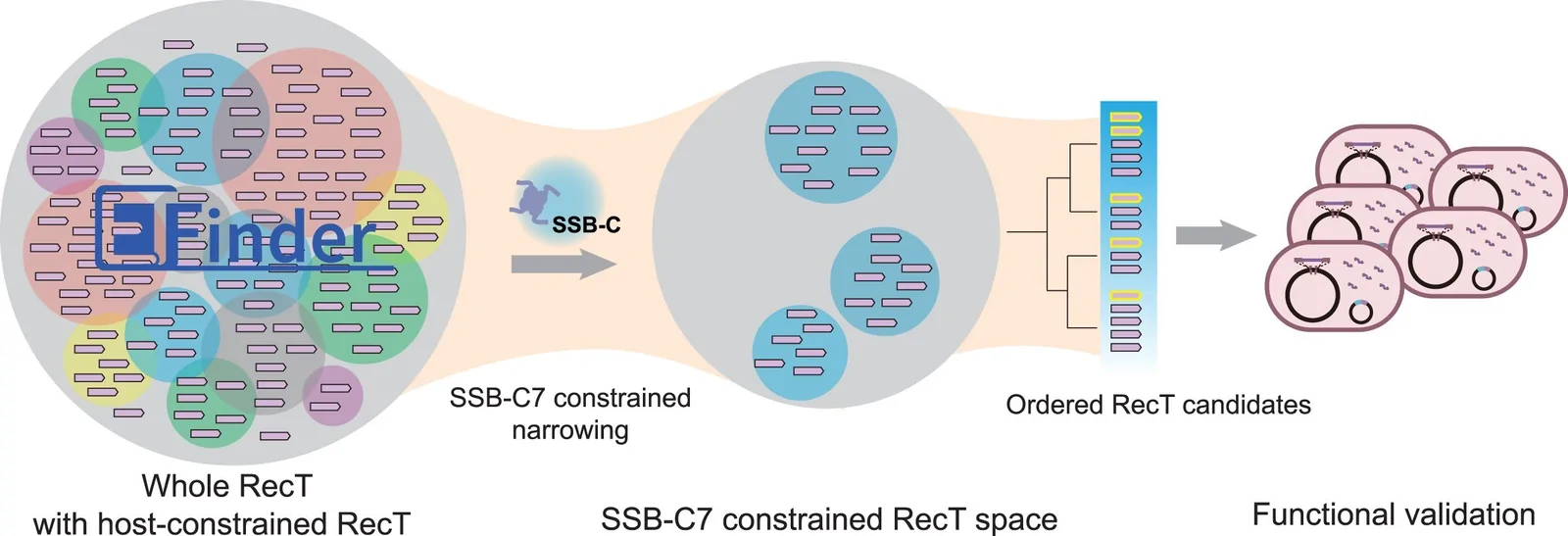

## Introduction

Next-generation microbial chassis (NGMCs), such as photosynthetic bacteria, thermophiles, acidophiles, and halophiles, are derived from extreme environments or possess unique physiological traits, demonstrating great potential for bioproduction applications [1–5]. However, most NGMCs are nonmodel organisms or have been minimally explored. The gene editing tools and techniques available for these organisms are far behind those developed for widely used model chassis, such as *Escherichia coli, Bacillus subtilis*, and *Saccharomyces cerevisiae* [6,7]. This gap in tools and technologies has become a significant obstacle to the development and application of NGMCs, limiting progress in synthetic biology and biomanufacturing.

Gene editing tools derived from bacteriophage-encoded DNA recombination systems (BEDRS), such as RecET and Redαβ in *E. coli* [8, 9], are well-known for their simplicity and efficiency in genome editing (GE). RecE/Redα generates 3′ single-stranded DNA (ssDNA) overhangs from double-stranded DNA (dsDNA), whereas RecT/Redβ binds these overhangs as a single-strand annealing protein (SSAP) to promote recombination [10, 11]. During replication, this recombinogenic complex anneals to homologous genomic DNA, enabling insertion of exogenous DNA into the genome [12]. Compared with RecA-dependent homologous recombination, which is often inefficient and labor-intensive in prokaryotes, RecET and Redαβ systems provide a simpler and more efficient route for GE [13, 14]. Because CRISPR/Cas editing also commonly requires homologous recombination (HR) -mediated repair of Cas-induced double-strand breaks [15, 16], developing efficient BEDRS-based tools remains important for both NGMCs and established chassis microorganisms.

However, the limited portability of BEDRS-derived GE tools requires repeated re-mining and characterization in individual microorganisms, restricting their broader use [17–19]. Accordingly, such tools have been established in only a few hosts, including *Mycobacterium tuberculosis*, lactic acid bacteria, Burkholderiales strains, and *Mycoplasma pneumoniae* [19–22]. Conventional 16S-guided searches prioritize RecET homologs from closely related species, but this strategy is unreliable because SSAPs such as RecT and Redβ are highly divergent and sequence similarity does not necessarily predict functional compatibility. Indeed, diverse SSAPs can function in a common *E. coli* host, indicating that recombination activity can extend beyond close taxonomic boundaries [23]. Therefore, a new selection criterion beyond taxonomic proximity is needed to capture conserved biochemical features of DNA processing across hosts.

Single-stranded DNA-binding (SSB) proteins play essential roles in DNA replication, repair, and recombination by stabilizing ssDNA intermediates and coordinating multiple DNA-processing enzymes [24]. As central hubs of genome maintenance, SSBs use conserved C-terminal motifs as docking sites for diverse interaction partners [25, 26]. Recent studies have shown that the functional compatibility of SSAPs is closely associated with host SSBs [27]. Structural and mutational analyses further revealed that the C-terminal helix of λ Redβ directly engages the last seven residues of *E. coli* SSB (SSB-C7), forming a defined biochemical interface for recruitment to ssDNA [26]. However, SSB–SSAP interactions have been experimentally confirmed in only a few model systems, and their generality across diverse bacterial hosts remains uncertain. It also remains unclear whether similar compatibility rules apply to distantly related RecT homologs or whether alternative loading mechanisms have evolved in other lineages. Nevertheless, these findings suggest that SSBs not only orchestrate host DNA metabolism but also shape a biochemical environment that constrains phage-derived recombination proteins. We therefore reasoned that SSB-guided compatibility could provide an extended functional criterion for identifying RecET systems, shifting the search paradigm from phylogenetic relatedness toward biochemical compatibility.

To explore the generalizability of this SSB-guided constraint, we applied it to two taxonomically and physiologically distinct NGMCs: the photosynthetic α-proteobacterium *Rhodobacter sphaeroides* and the halophilic γ-proteobacterium *Halomonas bluephagenesis. Rhodobacter sphaeroides* is an established industrial chassis for coenzyme Q10 production [28–31] and has potential for producing lycopene [32, 33], β-carotene [34], porphyrins, heme [4], and high-purity biohydrogen [35, 36], whereas *H. bluephagenesis* is widely used for efficient polyhydroxyalkanoate production [37, 38]. Despite their biotechnological potential, RecET-based GE systems have not been systematically identified or established in either host. Here, guided by the SSB-C7 motif of *R. sphaeroides*, we developed ETfinder, an SSB-guided screening framework that prioritizes RecET homologs predicted to share a compatible biochemical interface with the target chassis. ETfinder identified multiple RecET homologs suitable for *R. sphaeroides*, enabling efficient GE across several strains. We further refined and applied the workflow to *H. bluephagenesis*, demonstrating its generalizability and enabling robust gene editing in this halophilic chassis. To facilitate broader use, we packaged ETfinder as a user-friendly, locally deployable web tool for RecET-based GE development across diverse microorganisms.

## Materials and methods

### Bacterial strains and medium

All bacterial strains used in this study are listed in Supplementary Table S1. *Escherichia coli*-derived strains were cultured in Luria–Bertani (LB) medium containing 10 g/l NaCl, 10 g/l tryptone, and 5 g/l yeast extract at 37°C. *Rhodobacter sphaeroides* and its derivative strains were cultured in either a medium containing 5 g/l yeast extract, 2 g/l NaCl, 1.3 g/l KH_2_PO_4_, 0.125 g/l MgSO_4_, and 15 g/l agar, supplemented with 15 mg/l biotin, 1 mg/l thiamine hydrochloride, and 1 mg/l nicotinic acid, or in tryptone soya broth (TSB) prepared at 30 g/l, at 32°C. *Halomonas bluephagenesis* and its derivative strains were cultured in LB60 medium containing 10 g/l tryptone, 5 g/l yeast extract and 60 g/l NaCl, as previously described [39]. For electroporation, the super optimal broth with catabolite repression (SOC) medium was prepared with the following ingredients: 20 g/l of tryptone, 5 g/l of yeast extract, 0.5 g/l NaCl, 0.186 g/l KCl, 0.95 g/l MgCl_2_, 2.408 g/l MgSO_4_, and 3.6 g/l of glucose. For *Halomonas*, a high-salt SOC variant (SOC60) was used, in which the NaCl concentration was increased to 60 g/l. Sistrom’s minimal medium was prepared following the methods described previously [40]. The fermentation culture medium composition is as follows: 30 g/l glucose, 10 g/l MgSO_4_, 10 g/l CaCO_3_, 2.94 g/l monosodium glutamate, 2.94 g/l corn steep powder, 2 g/l NaCl, and 1.5 g/l K_2_HPO_4_, supplemented with 15 mg/l biotin, 1 mg/l thiamine hydrochloride, and 1 mg/l nicotinic acid. Antibiotic concentrations used in this study are listed in Supplementary Table S2. All strains constructed in this study were preserved using glycerol stocks.

### Plasmid, oligonucleotide, and conjugal transformation

The genes of the *recET* loci from five candidate species and oligonucleotides used were synthesized by Tsingke Biotechnology Co., Ltd. and then cloned into the pUC57 vector. The plasmids and primer sequences used in this study are listed in Supplementary Tables S3 and S4. The pIND4-oriT plasmid was linearized by double digestion with *Nco*I and *Hind*III (NEB). All insert DNA fragments used for plasmid construction were amplified using PrimeSTAR MAX DNA polymerase (TAKARA). The apramycin resistance gene cassette for plasmid or GE was amplified using 2x SuperTaq Mix (GeneStar). The amplified products were purified using Gel Extraction Kit (OMEGA Bio-Tek) and subsequently recombined using NovoRec^®^ Plus one step polymerase chain reaction (PCR) Cloning Kit (Novoprotein, China). The recombinant products were transformed into *E. coli* S17-1 competent cells by calcium chloride chemical transformation and plated on LB agar plates containing kanamycin. The plasmids were then transferred to *R. sphaeroides* by conjugal transfer. Briefly, fresh cultures of *R. sphaeroides* at an OD_700_ of 1.0 and *E. coli* at an OD_600_ of 0.6 were collected and washed. The cells were mixed at a 6:1 ratio and spotted onto nonselective plates overlaid with 0.22 μm nitrocellulose filters. After 20 h of incubation, the cells were scraped into TSB, diluted, and plated on selective plates containing nalidixic acid and kanamycin. Conjugation into *Halomonas* was performed as previously described [39].

### Recombineering via electroporation

Colonies of *R. sphaeroides* HY01, 2.4.1 or ZX-5 were picked from the plates and inoculated into 5 ml of TSB medium, with the cultures incubated until reaching an OD_700_ of 2–3. The culture was then diluted in fresh TSB to an OD_700_ of 1.0. When required, isopropyl β-D-1-thiogalactopyranoside (IPTG) was added to a final concentration of 0.5 mM, and the incubation continued until the OD_700_ exceeded 2.0. For induction, the cultures were induced for the durations specified in the corresponding experiments. Cells were harvested by collecting cultures equivalent to 2 OD units per tube. The cells were washed with 500 μl of sterile water or a specialized electroporation buffer (ddH_2_O for HY01 and 10% glycerol for wild type (WT) strains). This washing step was repeated twice, discarding 80% of the supernatant and leaving ∼100 μl of cell suspension with the electroporation buffer. The buffers used for recovery at either ice-cold or room temperature included 10% sucrose (S), 1 mM HEPES (H), 10% glycerol (G), combinations of sucrose and HEPES (SH), and deionized water (ddH₂O). Electroporation was performed with specific concentrations of plasmid or linear DNA fragments. For electroporation, an exponential decay program was used with the following parameters: for strain HY01, a voltage of 2500 V, a capacitance of 25 μF, and a resistance of 200 Ω were applied using a 2 mm cuvette, resulting in a typical pulse time of 5.0 ms. For *R. sphaeroides* strain 2.4.1, as described earlier [41], a resistance of 400 Ω was used, with the remaining parameters unchanged, yielding a typical pulse time of around 8.0 ms. Immediately after electroporation, 1 ml of antibiotic-free medium was added, and the cells were incubated at 32°C and 220 revolutions per minute for 2 h. The cultures were then plated on selective media, and positive transformants were identified and verified using previously described methods.

### Measurement of growth curves

Overnight cultures of *R. sphaeroides* HY01 were diluted to an OD_700_ of 0.05 in 250 ml Erlenmeyer flasks. The cultures were incubated, and samples were taken every 2–3 h to measure the OD_700_ using a CLARIOstar plate reader (BMG LABTECH). At selected time points (2 h, 5 h, 11 h, 15 h, 27 h, and 36 h), samples were also taken for electroporation experiments to assess the electroporation efficiency of the cells.

### Implementation of SSB-C7-based mining of RecET systems

ETfinder implements a unified framework that integrates an SSB-C7 motif constraint, RecT/RecE homology searches, and candidate scoring and ranking, and applies this framework through three operational modes. For *R. sphaeroides*, we used pre-downloaded RefSeq genome assemblies and construct a local protein BLAST database for each assembly. The C-terminal heptapeptide of the host SSB (SSB-C7) is used as a constraint to select assemblies carrying a matching SSB-C7, and RecT/Redβ and RecE/Redα candidates are then identified within this restricted set by PSI-/PHI-BLAST [42] searches seeded with family-specific position-specific scoring matrices (PSSMs). The resulting RecT/RecE hits are linked to 16S ribosomal DNA (rDNA) and annotation metadata to support subsequent scoring and prioritization. For *Halomonas*, we omit per-assembly local databases and instead query NR/RefSeq directly using the *H. bluephagenesis* D50 SSB-C7 as a seed, normalize hits to RefSeq assemblies via IPG/Assembly metadata, and perform SSAP homology searches and neighborhood-based RecE pairing only within this trimmed subset, integrating all candidates into the same scoring and ranking scheme. The web server operates entirely on a precomputed RecT–SSB–context dataset: all RecT-SSB pairs and their flanking Pfam-annotated neighborhoods are processed offline through a unified pipeline for scoring, classification, and ranking, and serialized into a versioned JSON mapping; online queries are resolved solely against this mapping to provide species-, protein-, and SSB-context views without invoking BLAST or HMMER at runtime. Full implementation details and command-line parameters are provided in the Supplementary Methods and Supplementary Datasets.

### Bioinformatic analysis

Multiple sequence alignments were performed using the ClustalW [43] algorithm within the MEGA software [44], applying default parameters. For the *R. sphaeroides* case, iqtree2 (v2.3.6) was used, whereas the remaining analyses were performed using the updated iqtree3 (v3.0.1) release, with the same parameter settings applied throughout [45]. An ultrafast bootstrap analysis was performed with 1000 replicates (-B 1000) [46], and the optimal model was automatically detected (-m MFP) [47]. The Bootstrap Nearest Neighbor Interchanges (BNNI) option (-bnni) was chosen. File format conversions were carried out using Mesquite software (version 3.81) [48] to convert the FASTA files generated from the multiple sequence alignments into the phylogenetic input format required for iqtree analysis. The resulting phylogenetic trees were visualized and refined using iTOL (https://itol.embl.de/) [49]. Protein structure predictions were performed using AlphaFold Server with random seeds [50]. The predicted protein structures were visualized and analyzed using UCSF ChimeraX (v1.9) [51].

### Analysis of SSB-C7 identity in established Red system

To evaluate the functionality of λ Red system or strain-specific RecET systems in Red/ET- established bacterial hosts and their relationship with the corresponding host SSB proteins, relevant studies were examined. Specifically, the focus was on the last seven amino acids of the SSB proteins (SSB-C7) in both the recipient host (SSB-C7-R) and the donor host from which the RecET system originates (SSB-C7-D). The sequences of SSB-C7-D and SSB-C7-R were compared by quantifying the differences in their seven amino acid residues. The differences were measured in terms of the number of amino acid mismatches and their percentage of the total SSB-C7-D/R pairs. For the λ Red system, its activity in recipient strains was recorded as “Active” if reported active and “Inactive” if reported inactive. The SSB-C7 differences were assessed based on their deviation from the *E. coli* SSB-C7 sequence FDDDIPF. This deviation was quantified using substitution scores from the BLOSUM62 [52] matrix for the corresponding amino acids. The final score was calculated by subtracting the substitution scores of mismatched amino acids from the total score of completely identical substitutions.

### Data analysis

Statistical analyses were performed in GraphPad Prism version 10.2.0. Data are expressed as the mean ± standard deviation (SD). Student’s *t*-tests were used for statistical analysis, and *P* <.05 indicated statistical significance.

## Results and discussion

### SSB-C7 sequence analysis, BLAST-based algorithm design, and workflow of ETfinder

**Figure 1.**
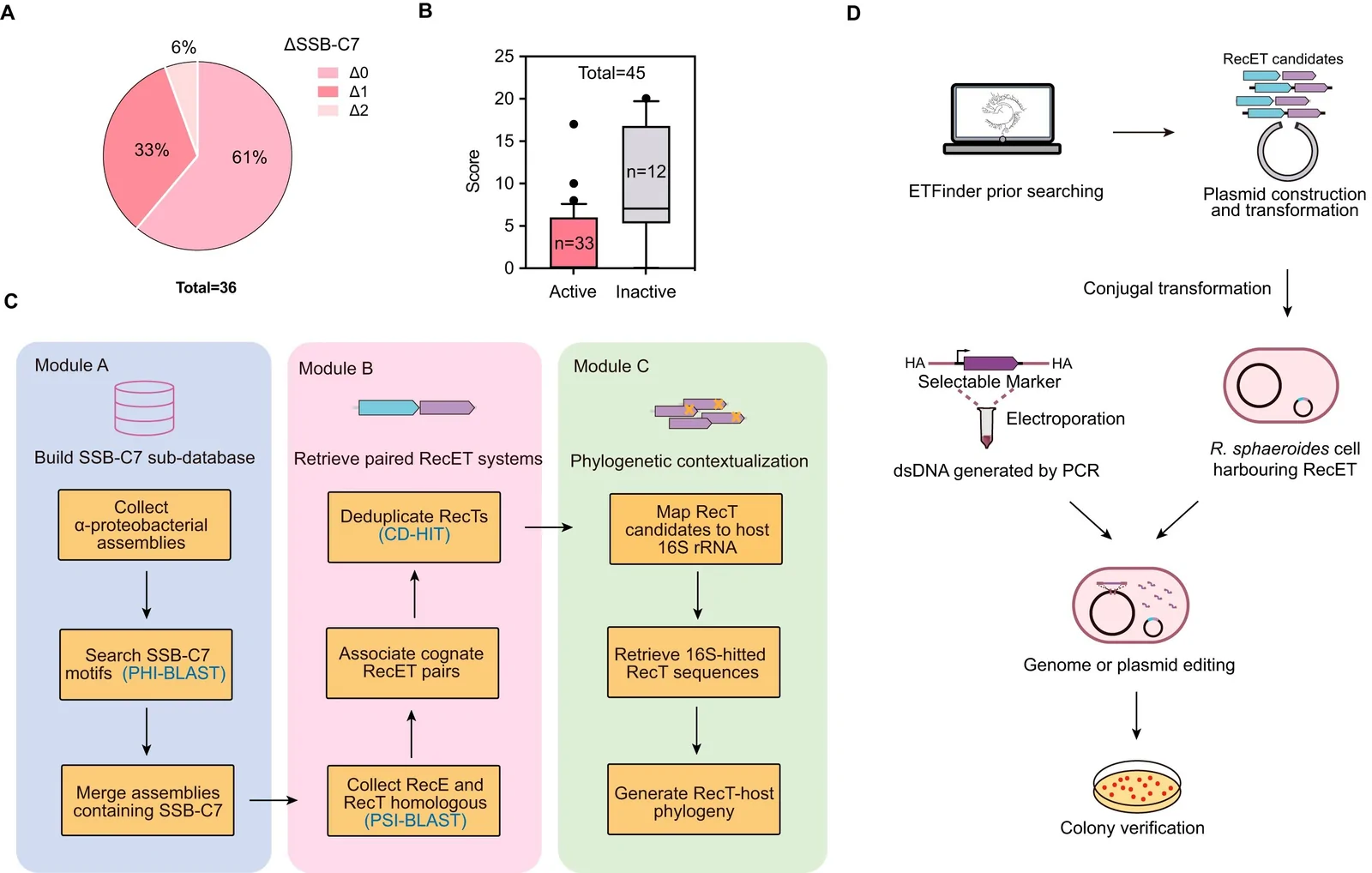
BLAST-based algorithm assists in mining potential RecET homologs from databases for target microbes. (**A**) Statistical analysis of SSB-C7 sequence in recipient and donor microbes. Δ0, Δ1, and Δ2 indicate the number of amino acid residue differences between SSB-C7-R and SSB-C7-D; for example, 0 indicates identical amino acid sequences. (**B**) Analysis of λ Red system application in non-*E. coli* microorganisms using BLOSUM62 scoring matrix. ‘Active’ represents gene editing activity observed according to the reports, while ‘Inactive’ indicates no observed gene editing activity. (**C**) ETfinder algorithm workflow for mining heterologous RecET homologs. The selected α-proteobacterium and the SSB-C7 feature motif X-LDDEIPF used in PHI-BLAST are based on the example of *R. sphaeroides*. For other microorganisms, appropriate substitutions need to be made accordingly. (**D**) The workflow for validating the gene editing activity of the selected RecET homologs in target microbes (e.g. *R. sphaeroides*).

We first compiled 36 reported cases of dsDNA-mediated GE using heterologous RecET homologs (Supplementary Table S5). The SSB-C7 sequences of the recipient microorganisms (Receptors) and the corresponding RecET donor microorganisms (Donors, which naturally carry the genes encoding RecET homologs) were then analyzed. The seven C-terminal residues of SSB in Receptors and Donors are hereafter referred to as SSB-C7-R and SSB-C7-D, respectively. SSB-C7-R and SSB-C7-D were identical in 61% (22/36) of cases and differed by only one residue in 33% (12/36); only 6% (2/36) differed by two residues (Fig. 1A). To further evaluate this relationship, we used the BLOSUM62 scoring matrix to examine 45 reported cases in which the λ Red system from *E. coli* was used for GE in non-*E. coli* microorganisms (Fig. 1B and Supplementary Table S6). Among the 33 cases with detectable Redαβ activity, >90% had matrix scores below 6, indicating high SSB-C7 similarity with *E. coli*. In contrast, the 12 inactive cases generally showed higher scores, suggesting lower SSB-C7 similarity. A few exceptions were observed, including *Lactobacillus casei* and *B. subtilis*, whose SSB-C7 sequences differ from that of *E. coli* by two to three residues but still support Redαβ editing at very low levels [53, 54]. Overall, the strong association between SSB-C7 similarity and RecET editing activity across known cases suggests that SSB-tail compatibility, reflecting the C-terminal recruitment function of SSB, can serve as a practical proxy for host–RecT interface compatibility.

These findings suggest that SSB-C7 compatibility may serve as a useful criterion for prioritizing heterologous RecET systems for functional deployment. To systematically identify compatible RecET homologs, we developed ETfinder, a multi-stage algorithm integrating SSB-C7 motif filtering, RecET operon mining, and phylogenetic contextualization (Fig. 1C). In Module A, a sub-database of α-proteobacterial assemblies was constructed, and PHI-BLAST was used to identify sequences encoding SSB proteins whose C-terminal motifs matched the SSB-C7 of the target host, such as *R. sphaeroides*, thereby defining a motif-constrained search space. In Module B, PSI-BLAST was used to identify RecE and RecT homologs from these assemblies. Candidate RecE–RecT pairs were automatically assigned according to genomic context and retained only when the two genes were adjacent and consecutively ordered in the original GenBank annotation. Redundant pairs were clustered and removed using CD-HIT at 95% identity to generate a nonredundant candidate set. In Module C, the 16S rDNA sequence corresponding to each retained RecT homolog was retrieved for phylogenetic mapping, enabling candidate ranking based on both SSB-C7 compatibility and host evolutionary proximity. Because this algorithm is designed for dsDNA-based editing, it specifically prioritizes complete RecE–RecT pairs to preserve structural and functional integrity; for ssDNA-based methods, such pairing may not be required [18, 55].

Collectively, this integrated workflow enables high-throughput, motif-guided mining of RecET systems predicted to be compatible with the target SSB environment and provides a rational basis for prioritizing homologs for functional testing. Candidate RecET genes identified by ETfinder were then cloned into expression plasmids and introduced into the target chassis, and their recombination activity was evaluated using dsDNA editing assays with selectable-marker substrates (Fig. 1D). Detected recombination events validated the activity of the mined RecET homologs, and all scripts and database resources supporting this pipeline are provided in the Supplementary Methods.

### Mining RecET homolog candidates from *α-proteobacteria* for *R. sphaeroides*

**Figure 2.**
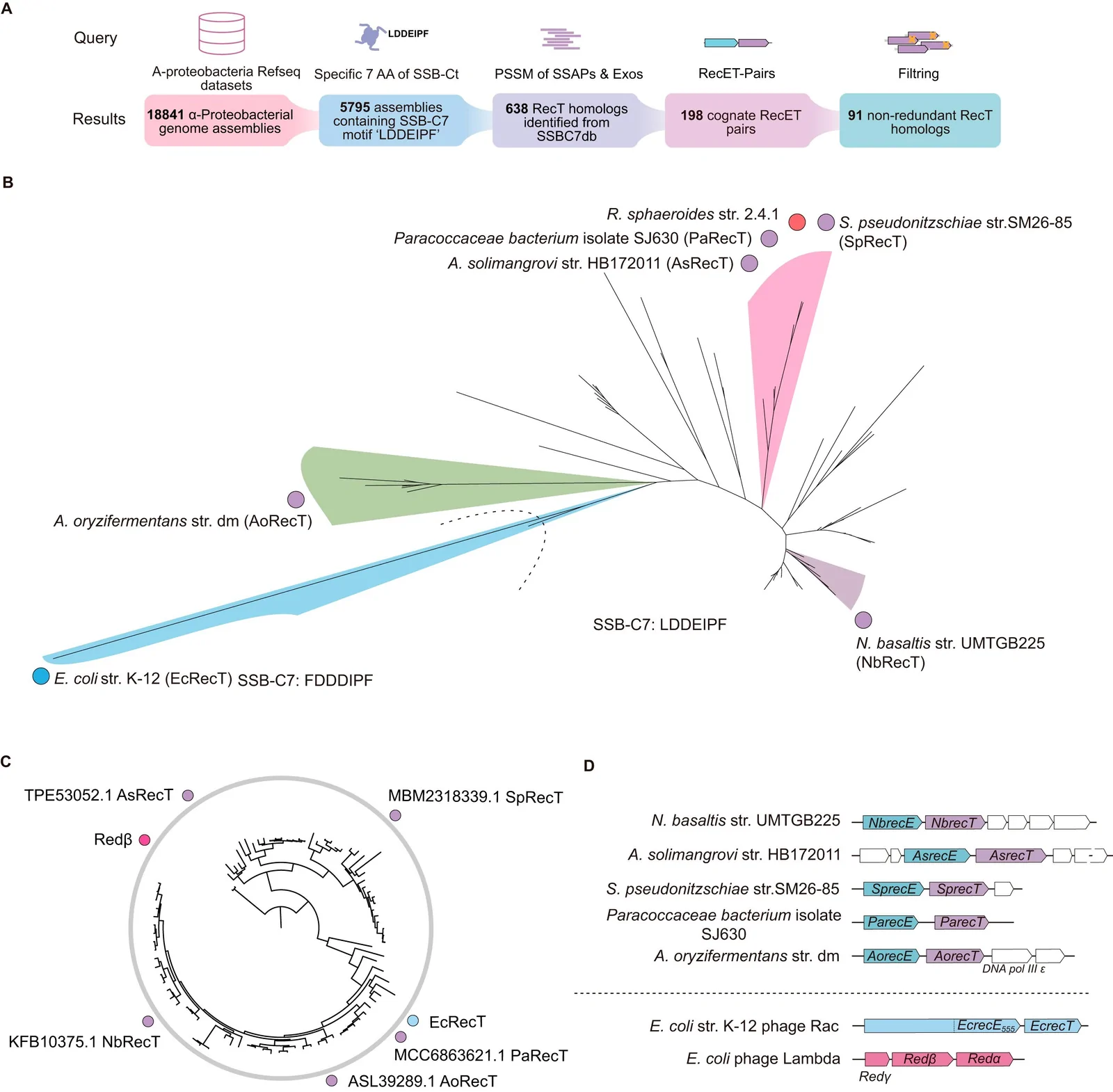
Mining and selection of RecET homologs from NCBI database for gene editing in *R. sphaeroides*. (**A**) Mining of RecET homologs from α-proteobacterial genomes. Numbers in bold represent the remaining hits after each round of conditional filtering. (**B**) 16S rDNA evolutionary tree of 91 microorganisms naturally hosting RecET homologs with that of *E. coli* and *R. sphaeroides* for reference. The microorganisms to which the selected RedET homologs belong, the nodes of *R. sphaeroides* and *E. coli*, are represented by purple, red, and blue solid dots, respectively, on the evolutionary tree, while different background colors indicate distinct evolutionary branches. (**C**) Evolutionary tree of 91 RecT hits obtained through the algorithm with Redβ and EcRecT. The positions of the selected RecT homologs, Redβ and EcRecT in the evolutionary tree are marked with purple, magenta, and blue solid dots, respectively. (**D**) The arrangement of the original operon of the five selected RecET homologs in their respective genomes.

To test this mining algorithm, we applied it to identify RecET homologs potentially active in *R. sphaeroides*. No complete endogenous RecET pair was detected in *R. sphaeroides*, and the only apparent *recET*-like locus appeared to be disrupted (Supplementary Fig. S1). Protein and GenBank files from all 18 841 α-proteobacterial assemblies available in the NCBI Assembly database as of 22 July 2023, were retrieved (Fig. 2A). PHI-BLAST identified 5795 assemblies encoding SSB proteins with the *R. sphaeroides* SSB-C7 motif, LDDEIPF. From these assemblies, PSI-BLAST retrieved 638 RecT homologs. Genomic-context analysis further identified 198 cognate RecE–RecT pairs. After removing redundancy based on host 16S rDNA similarity, 91 unique RecET homolog pairs were retained as candidates.

A phylogenetic tree was constructed using the 16S rDNA sequences of the corresponding hosts, with *R. sphaeroides* and *E. coli* included as references (Fig. 2B). To explore recombination compatibility beyond phylogenetic proximity, we selected three RecET homologs from species closely related to *R. sphaeroides* and two from more distant taxa for experimental evaluation. Because phage-derived SSAPs may not strictly co-evolve with their bacterial hosts, and to avoid testing highly similar RecT homologs, we also constructed a phylogenetic tree based on the 91 RecT sequences. *Escherichia coli* RecT and Redβ were included as references. The five selected RecT homologs were distributed across distinct regions of the RecT phylogeny, rather than clustering within a single closely related subgroup (Fig. 2C), indicating substantial sequence diversity among the selected homologs.

The five selected RecET pairs were derived from different bacterial strains: *Paracoccaceae* bacterium isolate SJ630 (PaRecET), *Sulfitobacter pseudonitzschiae* str. SM26-85 (SpRecET), *Acetobacter oryzifermentans* str. dm (AoRecET), *Nitratireductor basaltis* str. UMTGB225 (NbRecET), and *Amaricoccus solimangrovi* str. HB172011 (AsRecET). Detailed information on these RecT homologs is provided in Supplementary Table S7. All five RecET pairs displayed the canonical co-directional *recE–recT* arrangement (Fig. 2D). In some loci, *recE* and *recT* were embedded within larger multigene operons, as observed for *A. solimangrovi*, whereas in others they formed an independent bicistronic unit, as observed for *Paracoccaceae* bacterium SJ630. These architectures are consistent with the expected organization of functional RecET modules. Therefore, the five RecET homolog pairs were selected for testing gene-editing activity in *R. sphaeroides*.

### Testing gene editing activity of selected RecET homologs and developing GE tools in *R. sphaeroides* HY01

**Figure 3.**
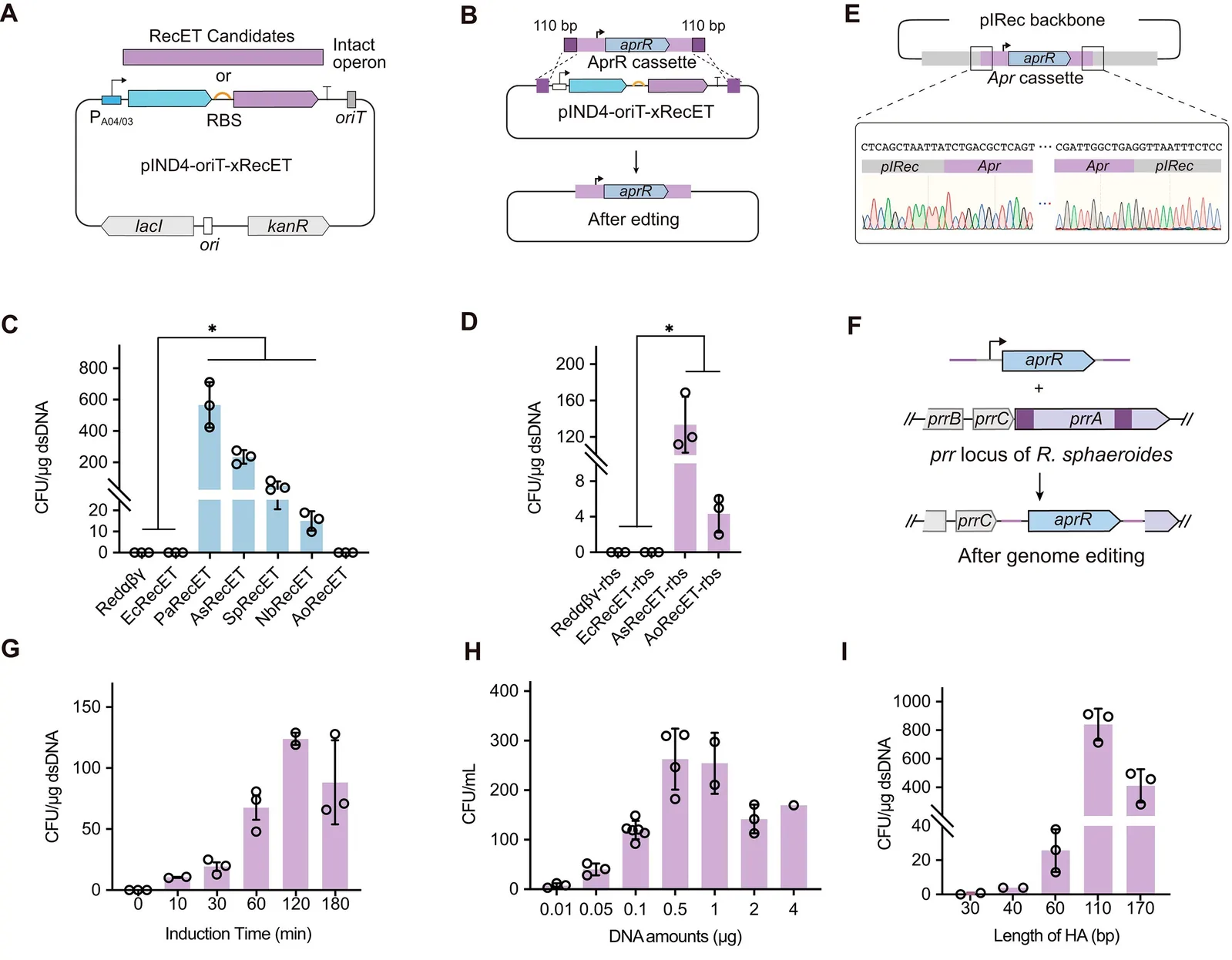
Evaluation of RecET homologs (PaRecET, AsRecET, SpRecET, NbRecET, and AoRecET) for gene editing in *R. sphaeroides* HY01. (**A**) Plasmid design for inducible expression of RecET homologs. RBS sequence: gacaccggaggaccccttaa. (**B**) Diagram illustrating dsDNA-mediated gene editing of RecET homologs. The homologous arms at both ends of the dsDNA target the *recE* homolog’s upstream and *recT* homolog’s downstream regions, respectively. (**C**) Plasmid editing activity of different RecET homologs in HY01. Following induction of RecET expression, 1 μg of dsDNA was introduced by electroporation, and recombinants were quantified as CFU on apramycin-containing plates. For AsRecET, editing was quantified using a donor with 400-bp homology arms, whereas all other RecET systems were assayed with donors carrying 110-bp homology arms. (**D**) Plasmid editing activity of different RecET homologs after applying the high-activity RBS upstream of *recT* homologs. All experimental conditions were identical to those outlined in Supplementary Fig. S2C, except for the RBS replacement. (**E**) Verification of PaRecET-mediated plasmid editing by Sanger sequencing. (**F**) Diagram of dsDNA-mediated PaRecET knockout of the *prrA* gene in the HY01 genome. (**G**) Effects of PaRecET induction duration on GE efficiency in HY01. The final concentration of IPTG used for induction is 0.5 mM. (**H**) Effects of dsDNA concentration on GE efficiency in HY01. (**I**) Effects of dsDNA homology arm length on GE efficiency in HY01. For panels (C), (D), and (G)–(I), bars represent mean values, error bars indicate SD, and open circles represent individual biological replicates.

We next tested the five ETfinder-selected RecET systems in HY01, together with the *E. coli*-derived Redαβγ and RecET systems. All recombinase operons were placed under the control of the IPTG-inducible P_A1/O4/O3_ promoter [56] (Fig. 3A) and evaluated under optimized electroporation conditions (Supplementary Fig. S2A–C). To avoid homology-length limitation, we used a linear apramycin-resistance cassette flanked by 110-bp homology arms. Because plasmids are generally easier to edit than chromosomal loci owing to their higher copy numbers [36, 57], we first targeted the *recET* region on pIND4 plasmid (Fig. 3B). Both *E. coli*-derived systems showed no detectable activity, consistent with previous results. In contrast, PaRecET, AsRecET, SpRecET, and NbRecET all supported dsDNA recombineering, albeit with markedly different efficiencies, whereas AoRecET was initially inactive (Fig. 3C). To improve AoRecT expression, we replaced its native ribosome-binding site (RBS) with a highly active endogenous RBS from HY01 [33], with the same substitution introduced into AsRecT, Redβ, and RecT as controls. This RBS substitution enabled detectable but low AoRecET activity, while AsRecET remained highly active and the *E. coli-*derived systems remained inactive (Fig. 3D). PCR screening of randomly selected colonies confirmed a 100% positive rate (Supplementary Fig. S2D), and sequencing verified the accuracy of the recombination events (Fig. 3E).

After RBS optimization, all five ETfinder-selected RecET systems showed detectable gene-editing activity in HY01. The high hit rate across both close and distant taxa supports the utility of ETfinder for identifying functionally compatible RecET systems beyond phylogenetic proximity. Compared with traditional phylogeny-based searches [18], ETfinder achieved a high success rate in this test set. Consistent with the SSB-guided hypothesis, homologs from taxa more closely related to HY01 showed higher editing efficiencies, whereas distant homologs remained functional but generally less active (Figs 2B, and 3C and D). This pattern may reflect shared SSB-tail motifs as well as broader DNA-processing contexts, including replication/repair factors and regulatory environments that facilitate RecT recruitment and recombineering. Thus, evolutionarily closer candidates may be prioritized when higher editing activity is desired, whereas the SSB-guided strategy can still recover active systems from distant lineages.

Recent work by Zakharova *et al*. showed that the aspartate residue at the eighth position from the C-terminus of *E. coli* SSB also contributes to compatibility [58]. We therefore searched GCAdbs for the SSB-C8 pattern “X-DLDDEIPF”, yielding 4352 hit assemblies. Among them, 4342 overlapped with the C7-based results, covering 99.77% of the C8 hits and 74.93% of the C7 hits (Supplementary Table S8). Thus, C8 filtering represents a more stringent subset of the C7-based search. Because the terminal seven residues of SSB-Ct have established functional relevance [27], and C7 provided sufficient candidate coverage to validate the SSB-Ct-guided discovery workflow, we retained C7 as the primary query for subsequent ETfinder development and candidate prioritization.

To optimize GE in HY01, we selected *prrA* [59], whose disruption produces a colorless phenotype (Supplementary Fig. S2B), as a visible reporter locus (Fig. 3F). Using PaRecET as the recombineering module, we systematically evaluated three parameters: PaRecET induction time, homology-arm (HA) length, and linear dsDNA amount. Editing activity was detectable after 10 min of induction and increased progressively, reaching a plateau at ∼120 min (Fig. 3G), followed by a slight decline that may reflect an imbalance between RecE and RecT levels during prolonged induction [8, 60]. Similarly, ≥0.1 µg linear dsDNA was sufficient for robust editing (Fig. 3H). HAs as short as 30 bp supported detectable recombination, with maximal efficiency at 110 bp (Fig. 3I). Under optimized conditions (60 min induction, 60–110 bp HAs, and ≥0.1 μg dsDNA), PaRecET-mediated editing reached up to 892 CFU per µg DNA, with a 100% editing accuracy confirmed by PCR and sequencing (Supplementary Fig. S2D and E).

### Plasmid curing and application of GE tools in other *Rhodobacter* strains

**Figure 4.**
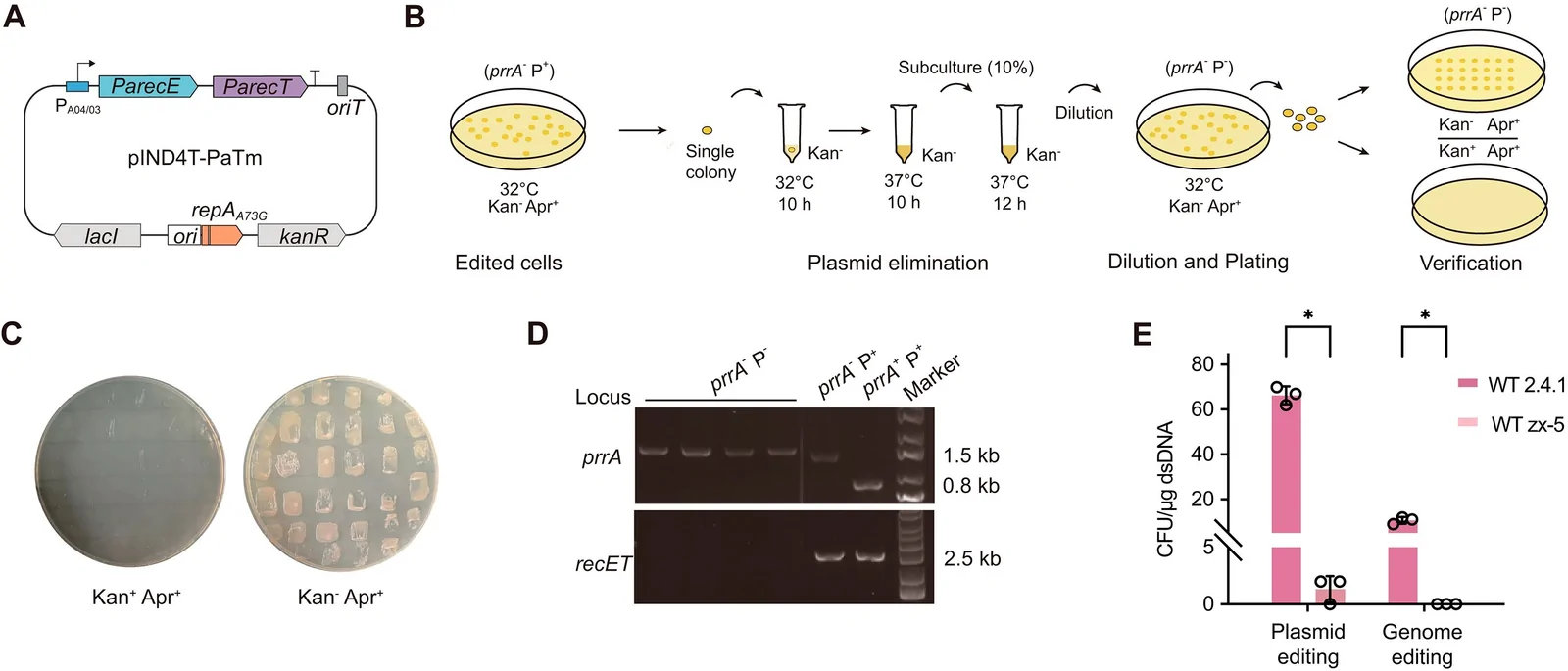
Plasmid curing in *R. sphaeroides* HY01 and gene editing activity of PaRecET in *R. sphaeroides* 2.4.1 and *R. sphaeroides* ZX-5. (**A**) Design of the temperature-sensitive plasmid pIND4T-PaTm. (**B**) Workflow of plasmid curing. (**C**) Testing plasmid curing efficiency on antibiotic plate after 37°C cultivation. (**D**) Verification of plasmid curing by PCR amplification of specific fragments. (**E**) PaRecET-mediated plasmid and GE in *R. sphaeroides* strains 2.4.1 and ZX-5.

To generate plasmid-free edited strains, we introduced a temperature-sensitive replicon based on RepA^A73G^ [61] into pIND4-*oriT*-PaRecET, yielding pIND4T-PaTm (Fig. 4A). This modification did not compromise PaRecET-mediated recombination efficiency in HY01. After cultivation at 37°C, a temperature above the HY01 growth optimum (32°C) and nonpermissive for plasmid replication, none of the 32 randomly selected transformants grew on kanamycin plates, whereas all grew normally on nonselective medium (Fig. 4B and C). PCR analysis detected no plasmid-specific bands (Fig. 4D), confirming efficient plasmid curing by temperature elevation.

Having established efficient curing in HY01, we next assessed the transferability of PaRecET in *R. sphaeroides* 2.4.1 and ZX-5. Strain 2.4.1 is a model photosynthetic bacterium that shares identical 16S rDNA with HY01 but differs in envelope physiology and plasmid maintenance [62, 63]. Using a reported electroporation protocol [41], PaRecET enabled both plasmid and chromosomal recombineering in 2.4.1 (Fig. 4E and Supplementary Fig. S3A). In ZX-5, however, its intrinsically low transformation efficiency restricted PaRecET activity to detectable plasmid editing (Fig. 4E and Supplementary Fig. S3B). These results indicate that PaRecET functions across multiple *R. sphaeroides* strains, although genome-level editing remains constrained by host-specific transformation barriers.

We further benchmarked PaRecET against reported *R. sphaeroides* editing methods, including homologous recombination [64], CRISPR/Cas [65], and site-specific recombination [66]. PaRecET showed higher efficiency and practicality, completing the genome-editing workflow within a single cultivation cycle (4–7 days; Table 1), a timescale not previously achieved in this organism. Together, these results establish PaRecET as the most rapid and efficient genome-editing system currently reported for *R. sphaeroides*.

**Table 1.** Comparison of GE methods in *R. sphaeroides*

Method	PC	TM	GE			LHA (bp)	PR	Time ^a^	Ref
			Replacement	Deletion	Insertion				
HR	Yes	Conj	Yes	Yes	Yes	>500	<15%	>14 d	[64]
CRISPR/Cas	Yes	Conj	PAM-dep	Yes	PAM-dep	>500	100%^b^	>6 d	[65]
Site-specific Recombination	Yes	Conj or Electro	attP-dep	attP-dep	attP-dep	/	100%	>6 d	[66]
RecET	No	Electro	Yes	Yes	No	∼50	100%	∼4 d	This study

a Time duration from designing gene replacement cassette to obtaining positive recombinants.

b 100% positive rate for *upp* deletion, 15% for *upp* base replacement. Abbr.: PC, plasmid construction; TM, transformation method; GE, genome editing; LHA, length of homology arm; PR, positive rate; HR, homologous recombination; Conj, conjugation; Electro, electroporation; PAM-dep, PAM-dependent; attP-dep, attP-dependent.

### Mining and evaluating gene editing activities of RecET homologs in *H. bluephagenesis*

**Figure 5.**
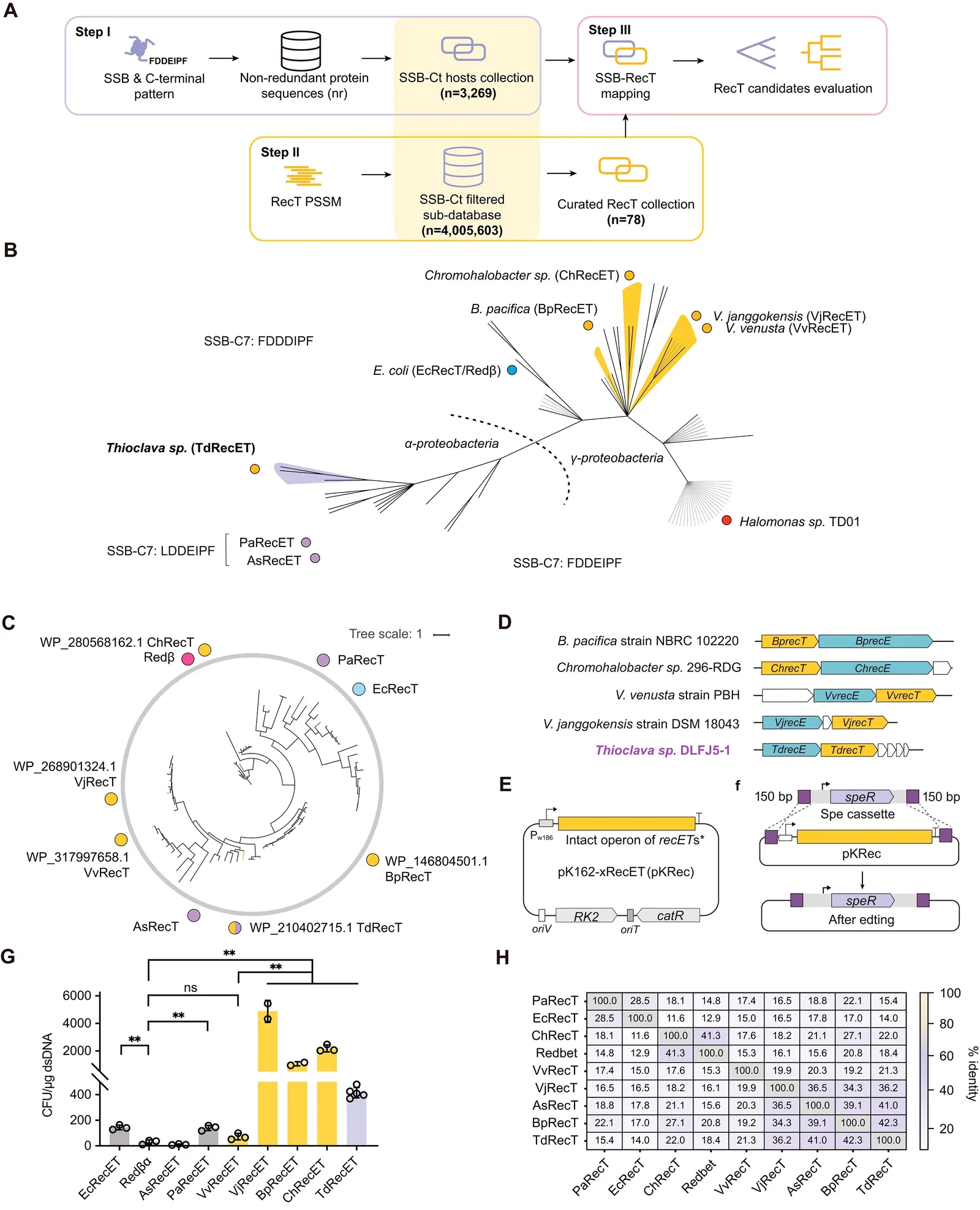
Mining and functional evaluation of RecET homologs predicted by ETfinder in *H. bluephagenesis* D50. (**A**) Overview of the SSB-Ct-guided mining strategy applied to *H. bluephagenesis* D50. PSI-BLAST using the FDDEIPF-pattern retrieved 3269 assemblies encoding matching SSBs, forming a sub-database of 4005 603 proteins, from which 78 nonredundant RecT homologs were identified. (**B**) Phylogenetic analysis of RecT homologs and their host species, revealing major lineages corresponding to γ-proteobacterial halomonads and α-proteobacteria. RecT homologs selected for experimental testing in *H. bluephagenesis* D50 are highlighted, including candidates from both closely related halomonads and more distant α-proteobacterial lineages. (**C**) Subtree showing representative RecT homologs selected for experimental testing. The yellow semicircle marks RecT homologs selected for testing in *H. bluephagenesis* D50, whereas the purple semicircle marks α-proteobacterial RecT homologs included in the *R. sphaeroides* case. PaRecT and AsRecT, shown in purple, are previously validated functional homologs in *R. sphaeroides*. Redβ and EcRecT are included as canonical reference proteins. Corresponding branches are colored using the same scheme. (**D**) Genomic organization of the selected *recT* operons in their native hosts. (**E**) Schematic of the test vector (pKRec) used for *Halomonas* recombineering assays. Asterisk indicates that the VvRecET construct contains only the VvRecE and VvRecT coding sequences to allow direct expression from the promoter. (**F**) Diagram of the plasmid editing assay design, in which a reporter cassette containing a *speR* gene was used to quantify recombination efficiency. (**G**) dsDNA recombination efficiencies of the RecET candidates expressed in *H. bluephagenesis* D50. Bars represent mean values, error bars indicate standard deviation, and open circles represent individual biological replicates. Gray bars indicate reference RecT proteins, yellow bars indicate RecET candidates selected from halomonads and related *γ-proteobacteria*, and the purple bar indicates TdRecET, an α-proteobacterial RecET candidate that exhibited relatively high activity in *H. bluephagenesis* D50. (**H**) Pairwise sequence identity matrix of the tested RecT proteins. Cell colors indicate the percentage of global amino-acid sequence identity, ranging from white to purple to gray according to the scale bar. The matrix shows that editing activity does not correlate with global sequence similarity.

To evaluate the portability of ETfinder in a physiologically distinct NGMC, we applied a streamlined SSB-C7-guided workflow to *Halomonas*. Instead of using a self-built genome database, we performed iterative NR/RefSeq searches and omitted nuclease–SSAP pairing (Supplementary Dataset S2). Starting from the host *ssb* sequence of *H. bluephagenesis* D50 and the FDDEIPF SSB-Ct pattern, PSI-BLAST retrieved 3269 assemblies encoding matching SSBs. These assemblies contained 4005 603 proteins, from which 78 nonredundant RecT homologs were identified (Fig. 5A).

Phylogenetic analysis of both hosts and SSAPs revealed two major clusters: γ-proteobacterial *Halomonadaceae* and α-proteobacterial hits. Reference RecTs (EcRecT, λ-Redβ, PaRecT, AsRecT) branched apart, highlighting the evolutionary divergence of the *Halomonas* candidates (Fig. 5B). Our SSB-guided survey showed that multiple evolutionarily distant RecT homologs were detected across closely related species and homologous sequences appeared in taxonomically distinct lineages (Supplementary Fig. S4A and B, see also Supplementary Fig. S5A and B), suggesting a mosaic distribution likely shaped by horizontal gene transfer. To capture this candidate diversity, we selected four candidates from non-*Halomonas* genera within *Halomonadaceae* that were positioned on dispersed RecT branches, together with one phylogenetically distant but SSB-Ct-matched α-proteobacterial candidate from *Thioclava* (Fig. 5C).

Functional validation in *H. bluephagenesis* D50 showed that three candidates from the *Halomonadaceae* (VjRecET, ChRecET, BpRecET) displayed robust dsDNA editing, whereas VvRecET was weakly active. Notably, TdRecET from *Thioclava*—phylogenetically distant from *Halomonadaceae* but sharing the same FDDEIPF SSB-Ct—retained moderate activity. In contrast, λ Redβ and AsRecET were inactive, while EcRecET and PaRecET exhibited only modest efficiencies (Fig. 5D–G).

Because intrageneric RecET systems were also available in *Halomonas* spp., this dataset further allowed us to ask whether extrageneric candidates are inherently less active than intrageneric homologs. We therefore tested three native RecET systems from *Halomonas* spp. as an intrageneric reference set (Supplementary Fig. S6A). All three were functional, with two displaying average efficiencies comparable to VjRecET (Fig. 5G and Supplementary Fig. S6B). Correct plasmid editing events were further confirmed by PCR verification, yielding amplicons of the expected sizes for all tested strains (Supplementary Fig. S7A–C). The comparable activities of native *Halomonas* RecETs and several RecET candidates from other *Halomonadaceae* genera indicate that RecET homologs from outside the target genus are not necessarily less active in *Halomonas*. Thus, ETfinder does not merely identify distant alternatives when native systems are unavailable but can define a functionally productive search space beyond the target genus. This observation is consistent with recent reports showing that SSAP/RecT variants can retain recombineering activity across phylogenetically distant bacterial hosts [67]. At the sequence level, active candidates could retain robust activity even with low global similarity: ChRecET and VjRecET shared only 18.2% sequence identity, yet both supported robust editing in *H. bluephagenesis* D50. Conversely, Redβ and AsRecT showed the highest sequence similarity among the tested RecT homologs but failed to function (Fig. 5H).

### ETfinder captures diverse RecT candidates beyond sequence similarity

By using a host-derived SSB-Ct constraint rather than similarity to a single reference RecT as the primary basis for candidate prioritization, ETfinder retained a broader range of naturally occurring RecT diversity, which may have contributed to its recovery of active candidates from different phylogenetic branches. This strategy enabled the recovery of distantly related RecTs with limited sequence similarity that remained functional in the same host (Supplementary Figs S4A and B, and S5A and B), revealing diversity beyond the conserved annealase core [68]. One aspect of this diversity is reflected in partner cooperation requirements. Evidence from *E. coli* RecT indicates that productive ssDNA filament formation can require RecE-mediated loading, and RecT shows lower oligonucleotide recombination activity than Redβ [69, 70]. Our ssDNA-editing assays in *R. sphaeroides* showed a similar tendency: only two newly identified variants generated the expected targeted mutations, whereas several homologs that supported dsDNA recombineering when paired with RecE produced only background mutations under the same conditions (Supplementary Fig. S8A–D). Such partner dependence would require RecT to retain features that support cognate RecE interaction [8], even when overall sequence similarity is limited (see also below).

**Figure 6.**
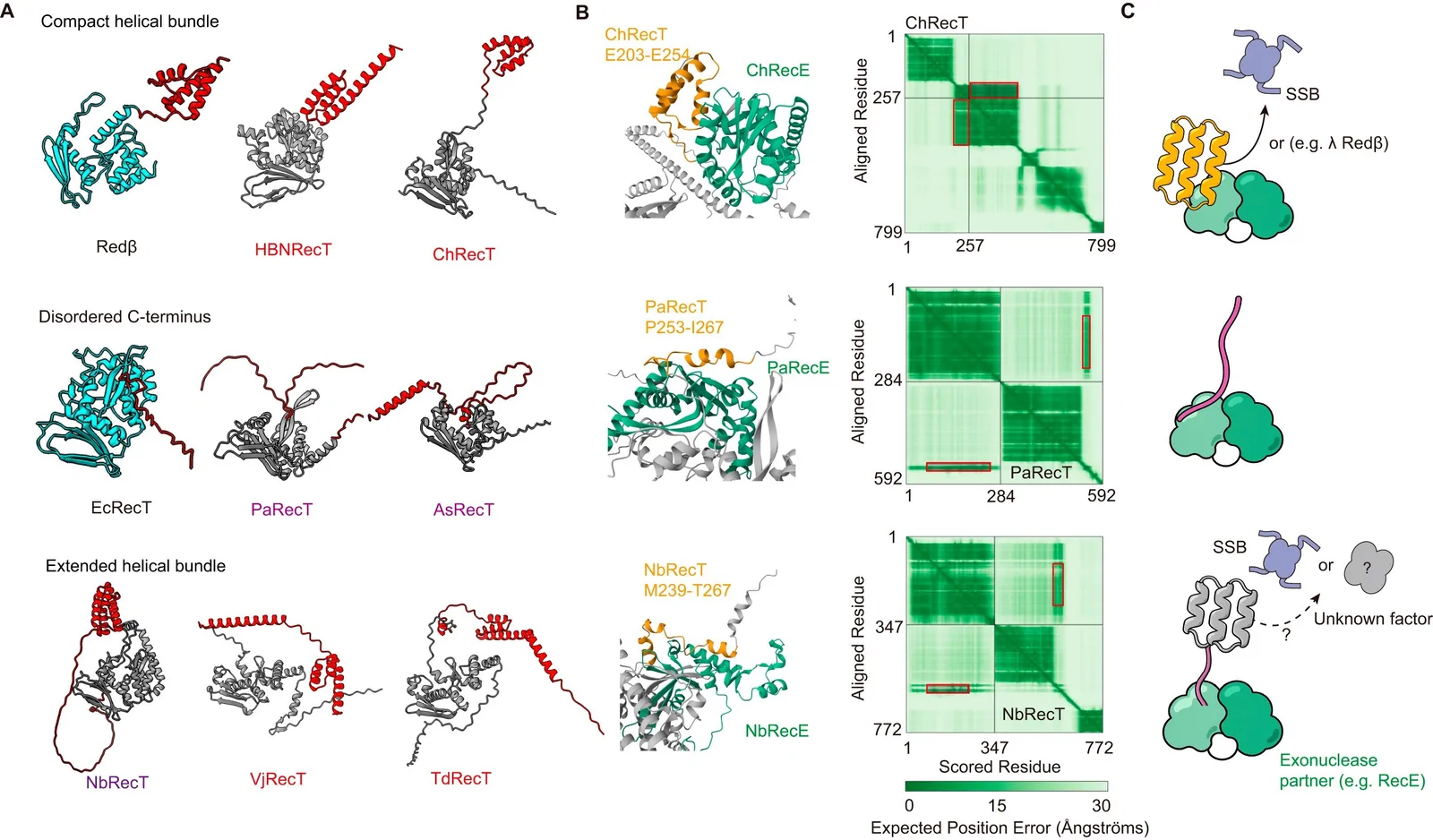
Structural diversity of RecT C termini suggests multiple C-terminal coupling modes. (**A**) Representative RecT structures grouped by predicted C-terminal architecture: C-terminal helical bundle, disordered C terminus, and extended C-terminal helical bundle. The N-terminal regions of the reference structures EcRecT and Redβ are shown in cyan, whereas the N-terminal regions of other RecTs are shown in gray. C-terminal regions are shown in red. (**B**) AlphaFold3 predictions of full-length RecT–RecE complexes. RecT segments corresponding to the boxed inter-chain regions in the predicted aligned error (PAE) maps are colored yellow, and the corresponding RecE regions are colored green. PAE maps are shown on the right, with darker green indicating lower PAE. Lower PAE values within the boxed inter-chain regions indicate higher confidence in the predicted relative positioning of the RecT and RecE regions. All structural predictions were performed in at least three independent runs. (**C**) Schematic model illustrating possible coupling modes of different RecT C-terminal architectures. C-terminal helical bundles may couple to SSB or exonuclease partners, disordered C termini may rely more strongly on auxiliary loading, and extended C-terminal helical bundles may provide additional coupling surfaces for SSB or other unknown host or phage factors.

This diversity was also reflected in C-terminal architectures outside the annealase domain. Several homologs exhibited similar C-terminal architectures despite substantial full-length sequence divergence, suggesting that RecT C termini represent a conserved structural feature within otherwise diverse RecT proteins (Supplementary Figs S6C–E and S9A–C). Structural predictions further revealed three broad C-terminal classes among the tested RecT proteins: a compact helical bundle, a disordered C terminus [71] and an extended helical bundle (Fig. 6A and Supplementary Fig. S10). All three supported RecET-mediated dsDNA recombination, indicating that no single C-terminal architecture is required for activity. In some predicted RecT–RecE complexes, the RecE-facing interface was formed mainly by residues in a loop or short helix near the C terminus rather than by the entire C-terminal bundle (Fig. 6A and B, and Supplementary Fig. S10). Extended elements outside this predicted interface may therefore provide accessory surfaces for SSB coupling or other host-factor interactions, analogous to the interactions of the Redβ C terminus with Redα and SSB-Ct [58, 72] (Supplementary Fig. S11A). Several RecT C-terminal extensions also showed fold-level similarity to specific regions of individual nucleic-acid-associated proteins, including a DEAD/DEAH-box-containing helicase, an RMF-like proteins, the C-terminal region of a NinB protein and UvsW.1 [73] (Supplementary Fig. S11B–E). These fold-level similarities suggest that RecT C-terminal extensions may represent functionally constrained regions rather than purely variable appendages, providing a structural basis for diverse loading or host-coupling scenarios (Fig. 6C).

Collectively, SSB-Ct-guided ETfinder mining therefore captures a compatibility space shaped by a conserved host-interface signal rather than relying on sequence similarity alone. This diversification may also reflect multiple recombination solutions that emerged as phages or prophages adapted to different host DNA-repair environments [74]. In this view, distinct RecT lineages may converge on productive recombination through diverse structural and loading strategies while retaining a conserved functional core. This view may explain why both closely related and distantly related RecTs can function in the same host, whereas sequence similarity alone does not reliably predict portability. Although the precise molecular determinants of RecT compatibility remain unclear, ETfinder provides a strategy to access diverse RecT solutions and may facilitate future engineering of recombineering portability in nonmodel microbial chassis.

### Development of ETfinder as a locally deployable toolkit for RecET discovery and prioritization

**Figure 7.**
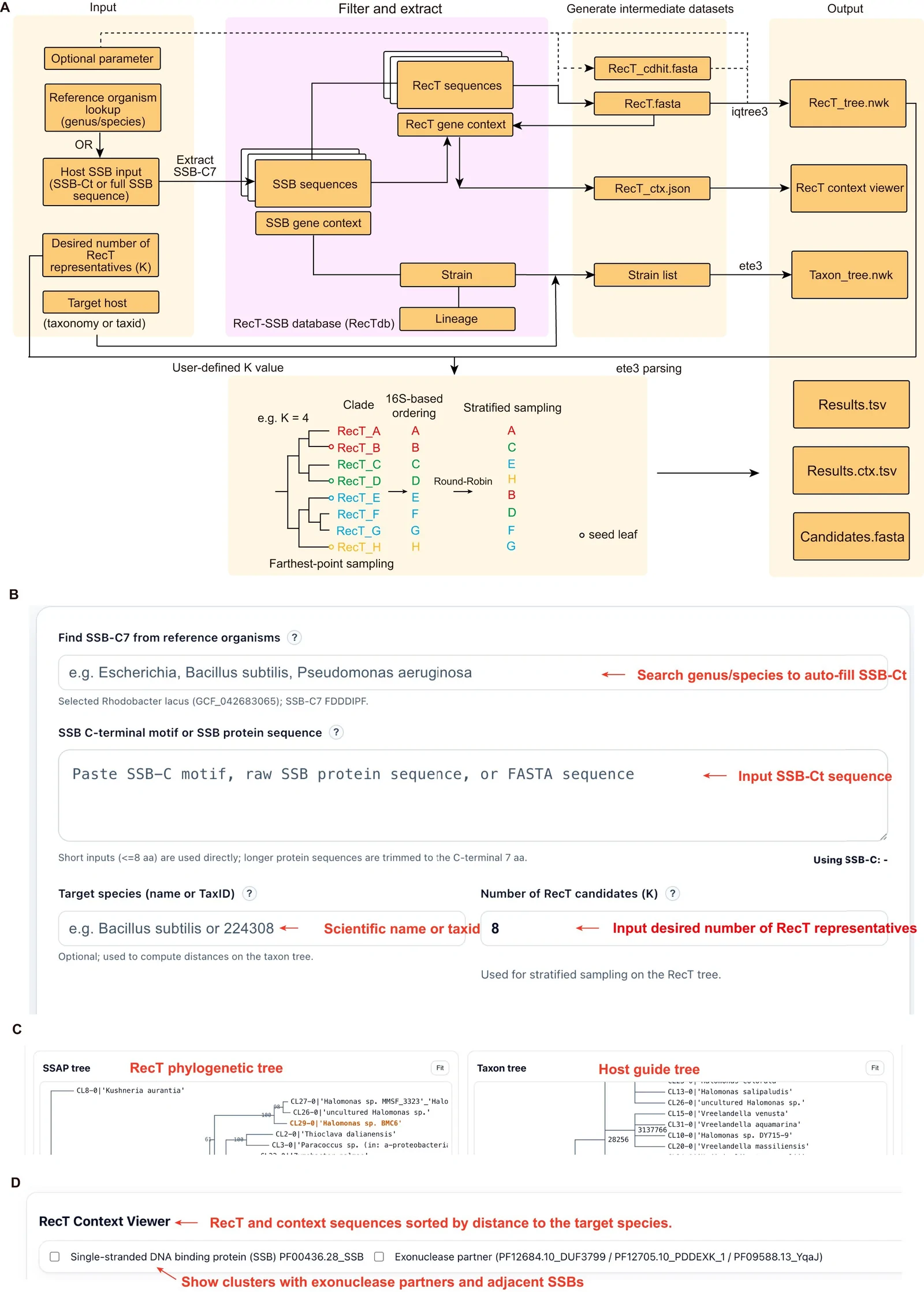
Overview of the ETfinder workflow and local web interface. (**A**) Schematic representation of the ETfinder workflow. Users provide host SSB information, an optional target host, and an optional desired number of RecT representatives (K). The host SSB input can be supplied through a reference organism lookup by genus or species name, as a short SSB C-terminal motif, or as a full-length SSB protein sequence, from which ETfinder extracts the C-terminal seven amino acids (SSB-C7). The resulting SSB-C7 motif is used to query the RecT–SSB database (RecTdb), retrieve matched SSBs, associated RecT proteins and their genomic contexts, and generate intermediate FASTA, context JSON, and strain-list files. RecT sequences are clustered and aligned for phylogenetic reconstruction, while host taxonomic information is parsed to build the corresponding host guide tree. Candidate prioritization is performed using the user-defined K value through phylogeny-aware stratified sampling and taxonomic-distance-based ordering, producing ranked result tables and candidate FASTA files. (**B**) Input interface of the ETfinder local web server. Users can search the built-in reference SSB table, paste an SSB-C motif or full-length raw/FASTA-formatted SSB protein sequence, specify the target species or TaxID, and define the desired number of RecT representatives. (**C**) Example output tree interface showing the inferred RecT phylogeny and the corresponding host guide tree. (**D**) RecT context viewer showing ranked RecT–SSB neighborhoods, with filters for clusters containing adjacent SSBs or exonuclease partners.

To facilitate broader adoption and reproducibility, we encapsulated the ETfinder core algorithm into a locally hosted web interface that runs entirely offline on a standard workstation. The program integrates SSB-Ct–guided homology search, clustering, and dual phylogenetic analysis modules, enabling users to identify RecT candidates compatible with virtually any bacterial or archaeal host through an intuitive browser-based interface (Fig. 7A). To enhance computational efficiency and ensure reproducibility, we built a local RecT protein database (RecTdb) from RefSeq seeded with RecT family profiles (PF03837 and TIGR01913). Each entry is linked to its endogenous SSB via assembly accession and annotated with lineage information, the host SSB-C7 motif, and ±2-gene neighborhoods (protein identifiers, products, coordinates, sequences, and optional Pfam annotations).

We first established an order-level reference for SSBs using all bacterial reference genomes available in NCBI Datasets [75] (as of 23 February 2025). SSB loci encoding the PF00436 OB-fold were identified, neighboring open reading frames (ORFs) (±2) were extracted, and proteins were annotated against Pfam-A, yielding 32 954 SSB contexts across 20 434 assemblies spanning ∼4103 genera. Family-level Pfam enrichments were then aggregated into order-level contextual clusters and applied genome-wide to classify SSB loci as host-type (*n* = 19 863), nonhost (*n* = 12 688), or suspect (*n* = 403). The order-level contextual clusters—constructed from significantly enriched Pfam co-occurrence patterns within bacterial families—recapitulate the conserved genomic neighborhoods of chromosomal SSBs and consequently enrich for SSBs carrying the canonical acidic C-terminal tail (Supplementary Fig. S12, see Supplementary Methods and Supplementary Datasets S3 and S4).

Using this reference, RecT–SSB pairs were identified by querying against a local NR snapshot (downloaded on 23 February 2025) using PSSM-seeded PSI-BLAST, generating a versioned JSON catalog of 25 529 RecT–SSB pairs with contextual annotation. This catalog represents the currently observable occurrence space of RecT homologs associated with canonical SSBs across sequenced bacteria; taxa lacking PF00436-type SSBs or carrying highly divergent ssDNA-binding proteins are outside the scope of this analysis (see Supplementary Methods).

To avoid over-concentrating prioritized candidates within a single branch of the RecT phylogeny, the same diversification logic that emerged during manual candidate selection was later implemented in ETfinder as a tree-aware stratified sampling procedure. First, a small set of K seed leaves (where K is user-defined) are chosen such that they are maximally separated along the RecT tree, thereby defining representative phylogenetic regions. All remaining RecT homologs are then assigned to the nearest seed and partitioned into a fixed number of groups, within which candidates are ranked by host taxon distance (Fig. 7A and B). Finally, ETfinder performs a round-robin traversal of these groups when generating the prioritized candidate list, interleaving phylogenetically distant RecT variants at the top of the table rather than returning a single dense clade. The detailed implementation of this stratified sampling algorithm is provided in the Supplementary Methods.

Because RecTdb and all scoring and mapping routines execute entirely on a local workstation, analyses are fully reproducible and require no network access. Users can initiate a search by specifying a target host taxonomic identifier together with the host SSB information (Fig. 7B). The SSB input can be supplied as a C-terminal motif, pasted as a full-length raw or FASTA protein sequence, or selected through a built-in reference organism lookup, after which ETfinder automatically extracts the C-terminal seven amino acids for SSB-guided RecT filtering. Users can also adjust optional parameters, including whether the RecT tree is built from cluster representatives or all sequences, the CD-HIT identity threshold, and the IQ-TREE substitution model (Supplementary Fig. S13A). ETfinder then queries RecTdb for entries with identical or highly similar motifs (labeled host or host_low), extracts the corresponding RecT loci, and performs clustering, multiple sequence alignment, and phylogenetic reconstruction. The RecT and host phylogenies are automatically linked through ETE3 and rendered as synchronized, interactive trees (Fig. 7C). Depending on computational resources and dataset size, each analysis can generally be completed within several minutes on a standard workstation, producing an annotated candidate table, a RecT phylogeny, a corresponding host subtree, and FASTA files for candidate sequences and context inspection (Supplementary Fig. S13B). The web interface also provides an optional genomic-context inspection module, allowing users to examine whether candidate RecT loci are accompanied by nearby SSB-domain-containing genes or exonuclease partners (Fig. 7D and Supplementary Fig. S13C). These features are provided as contextual annotations in the current version and may help users identify candidate loci in which native or mobile-element-associated SSBs could be considered in future SSAP–SSB co-expression tests.

The complete implementation is available at https://github.com/lvdongyuan/ETfinder. For demonstration and reproducibility, a Google Colab notebook version (ETfinder-Colab) is also provided, which reproduces the same workflow for users without a local Python environment. ETfinder thus provides a lightweight, browser-based yet locally executable framework for discovering and evaluating portable RecET recombination systems across diverse microbial hosts. As with any sequence-driven search, several limitations remain. Enrichment for putatively functional alleles does not ensure activity: some candidates may be inactivated by accumulated mutations, others may represent relics of horizontal transfer with no current phage function, and host-specific factors—including tolerance to foreign DNA—are difficult to anticipate. Prior work has demonstrated that strains of the same species can exhibit the entire spectrum of RecET outcomes—from no measurable activity to strong recombination [53]—consistent with our own observations (Fig. 4E). Thus, optimizing transformation and expression conditions in the target host remains essential for any RecET-based workflow [76]. While the current implementation focuses on RecT-based recombination modules, its motif-guided and colinearity-based architecture can, in principle, be generalized to other DNA-processing families, such as Erf or Sak. Moreover, its dual-phylogeny visualization offers a convenient framework for exploring RecT–host coevolution and for assessing system-level compatibility between recombination and defense modules, thereby extending the conceptual reach of recombineering studies beyond traditional phylogenetic boundaries.

## Conclusion

In this work, we establish an SSB-Ct-guided framework for discovering portable RecET recombineering systems and demonstrate its generality across taxonomically and physiologically distinct nonmodel microorganisms. Unlike approaches that rely on 16S rRNA similarity or Redβ-derived sequence probes, ETfinder uses the host SSB C-terminal heptapeptide as a biochemical filter, allowing RecT homologs with potential functional compatibility to be recovered even from distantly related species. By formalizing the conserved C-terminal motif of host SSBs as a biochemical constraint and integrating it with sequence profiling, contextual filtering, and dual phylogenetic analysis, ETfinder shifts RecET discovery from taxonomy-driven heuristics toward a compatibility-driven search principle. Application of this framework to 18 841 α-proteobacterial genomes yielded a focused candidate set for *R. sphaeroides*, all of which proved functionally active and enabled the development of the most rapid and efficient genome-editing system currently available for this chassis. Iterative deployment in the halophilic *Halomonas* further showed that SSB-Ct-guided mining enriches for RecT homologs capable of functioning across broad evolutionary distances, revealing a functional compatibility space not predicted by global sequence similarity alone.

To support broad application, we consolidated 25 529 RecT–SSB pairs into RecTdb and implemented ETfinder as a locally deployable, offline web toolkit integrating mining, ranking, phylogeny, and context visualization. Together, the biochemical constraint, experimental validation, and supporting informatics establish a coherent platform for accelerating RecET-based GE in diverse NGMCs. Importantly, our validation suggests that ETfinder’s constraint-driven approach provides a more informative route to functional compatibility than relying on primary-sequence similarity alone. As additional host-determinant factors are experimentally defined and structural insights into RecT loading and C-terminal coupling continue to accumulate, future versions of ETfinder may incorporate structure-aware filters or fold-based classifiers into the search pipeline. In this sense, ETfinder provides an extensible framework that can progressively absorb emerging structural insights, enabling increasingly accurate, host-tailored discovery of RecT systems and expanding the accessible recombineering landscape across previously inaccessible microbial lineages.

## Supplementary Material

gkag818_Supplemental_Files

## Data Availability

The ETfinder source code, together with the RecT-SSB database and the reference SSB database compiled in this study, is publicly available in Zenodo at https://doi.org/10.5281/zenodo.21399393. The GitHub repository at https://github.com/lvdongyuan/ETfinder serves as the project landing page and links to the permanently archived release. The input data and analysis files for the *Rhodobacter sphaeroides* and *Halomonas bluephagenesis* case studies are provided in Supplementary Datasets S1 and S2, respectively. All other data supporting the findings of this study are included in the article and its Supplementary Data.

## References

[1] Ye JW, Lin YN, Yi XQ. et al. Synthetic biology of extremophiles: a new wave of biomanufacturing. Trends Biotechnol. 2023;41:342–57. 10.1016/j.tibtech.2022.11.010. 36535816

[2] Adams BL. The next generation of synthetic biology chassis: moving synthetic biology from the laboratory to the field. ACS Synth Biol. 2016;5:1328–30. 10.1021/acssynbio.6b00256. 27665861

[3] Chen GQ, Jiang XR. Next generation industrial biotechnology based on extremophilic bacteria. Curr Opin Biotechnol. 2018;50:94–100. 10.1016/j.copbio.2017.11.016. 29223022

[4] Chen H, Wang Y, Wang W. et al. High-yield porphyrin production through metabolic engineering and biocatalysis. Nat Biotechnol. 2025;43:1717–27. 10.1038/s41587-024-02267-3. 38839873

[5] Jung H, Inaba Y, Banta S. Genetic engineering of the acidophilic chemolithoautotroph *Acidithiobacillus ferrooxidans*. Trends Biotechnol. 2022;40:677–92. 10.1016/j.tibtech.2021.10.004. 34794837

[6] Hwang S, Joung C, Kim W. et al. Recent advances in non-model bacterial chassis construction. Curr Opin Syst Biol. 2023;36:100471. 10.1016/j.coisb.2023.100471.

[7] Yang Z, Li Z, Li B. et al. A thermostable type I-B CRISPR-Cas system for orthogonal and multiplexed genetic engineering. Nat Commun. 2023;14:6193. 10.1038/s41467-023-41973-5. 37794017 PMC10551041

[8] Muyrers JP, Zhang Y, Buchholz F. et al. RecE/RecT and Redalpha/Redbeta initiate double-stranded break repair by specifically interacting with their respective partners. Genes Dev. 2000;14:1971–82. 10.1101/gad.14.15.1971. 10921910 PMC316823

[9] Zhang Y, Muyrers JP, Testa G. et al. DNA cloning by homologous recombination in *Escherichia coli*. Nat Biotechnol. 2000;18:1314–7. 10.1038/82449. 11101815

[10] Noirot P, Kolodner RD. DNA strand invasion promoted by *Escherichia coli* RecT protein. J Biol Chem. 1998;273:12274–80. 10.1074/jbc.273.20.12274. 9575178

[11] Erler A, Wegmann S, Elie-Caille C. et al. Conformational adaptability of Redbeta during DNA annealing and implications for its structural relationship with Rad52. J Mol Biol. 2009;391:586–98. 10.1016/j.jmb.2009.06.030. 19527729

[12] Maresca M, Erler A, Fu J. et al. Single-stranded heteroduplex intermediates in lambda Red homologous recombination. BMC Mol Biol. 2010;11:54. 10.1186/1471-2199-11-54. 20670401 PMC2918612

[13] Yu D, Ellis HM, Lee EC. et al. An efficient recombination system for chromosome engineering in *Escherichia coli*. Proc Natl Acad Sci USA. 2000;97:5978–83. 10.1073/pnas.100127597. 10811905 PMC18544

[14] Li R, Li A, Zhang Y. et al. The emerging role of recombineering in microbiology. Eng Microbiol. 2023;3:100097. 10.1016/j.engmic.2023.100097. 39628926 PMC11610958

[15] Wang S, Zeng X, Jiang Y. et al. Unleashing the potential: type I CRISPR-Cas systems in actinomycetes for genome editing. Nat Prod Rep. 2024;41:1441–55. 10.1039/D4NP00010B. 38888887

[16] Jiang W, Bikard D, Cox D. et al. RNA-guided editing of bacterial genomes using CRISPR-Cas systems. Nat Biotechnol. 2013;31:233–9. 10.1038/nbt.2508. 23360965 PMC3748948

[17] Bian Z, Li S, Yang R. et al. Development of a new recombineering system for *Agrobacterium* species. Appl Environ Microb. 2022;88:e0249921. 10.1128/aem.02499-21. PMC8904050 35044833

[18] Chang Y, Wang Q, Su T. et al. Identification of phage recombinase function unit in genus *Corynebacterium*. Appl Microbiol Biotechnol. 2021;105:5067–75. 10.1007/s00253-021-11384-x. 34131780

[19] Wang X, Zhou H, Chen H. et al. Discovery of recombinases enables genome mining of cryptic biosynthetic gene clusters in *Burkholderiales* species. Proc Natl Acad Sci USA. 2018;115:E4255–63. 29666226 10.1073/pnas.1720941115 PMC5939090

[20] van Kessel JC, Hatfull GF. Recombineering in *Mycobacterium tuberculosis*. Nat Methods. 2007;4:147–52. 17179933 10.1038/nmeth996

[21] van Pijkeren JP, Britton RA. High efficiency recombineering in lactic acid bacteria. Nucleic Acids Res. 2012;40:e76. 22328729 10.1093/nar/gks147 PMC3378904

[22] Pinero-Lambea C, Garcia-Ramallo E, Martinez S. et al. *Mycoplasma pneumoniae* genome editing based on oligo recombineering and Cas9-mediated counterselection. ACS Synth Biol. 2020;9:1693–704. 10.1021/acssynbio.0c00022. 32502342 PMC7372593

[23] Datta S, Costantino N, Zhou X. et al. Identification and analysis of recombineering functions from Gram-negative and Gram-positive bacteria and their phages. Proc Natl Acad Sci USA. 2008;105:1626–31. 10.1073/pnas.0709089105. 18230724 PMC2234195

[24] Kozlov AG, Weiland E, Mittal A. et al. Intrinsically disordered C-terminal tails of *E. coli* single-stranded DNA binding protein regulate cooperative binding to single-stranded DNA. J Mol Biol. 2015;427:763–74. 10.1016/j.jmb.2014.12.020. 25562210 PMC4419694

[25] Shereda RD, Kozlov AG, Lohman TM. et al. SSB as an organizer/mobilizer of genome maintenance complexes. Crit Rev Biochem Mol Biol. 2008;43:289–318. 10.1080/10409230802341296. 18937104 PMC2583361

[26] Shinn MK, Chaturvedi SK, Kozlov AG. et al. Allosteric effects of *E. coli* SSB and RecR proteins on RecO protein binding to DNA. Nucleic Acids Res. 2023;51:2284–97. 10.1093/nar/gkad084. 36808259 PMC10018359

[27] Filsinger GT, Wannier TM, Pedersen FB. et al. Characterizing the portability of phage-encoded homologous recombination proteins. Nat Chem Biol. 2021;17:394–402. 10.1038/s41589-020-00710-5. 33462496 PMC7990699

[28] Zhang L, Liu L, Wang KF. et al. Phosphate limitation increases coenzyme Q(10) production in industrial *Rhodobacter sphaeroides* HY01. Synthetic Syst Biotechnol. 2019;4:212–9. 10.1016/j.synbio.2019.11.001. PMC6909082 31890925

[29] Cluis CP, Burja AM, Martin VJ. Current prospects for the production of coenzyme Q10 in microbes. Trends Biotechnol. 2007;25:514–21. 10.1016/j.tibtech.2007.08.008. 17935805

[30] Shi T, Zhang L, Liang M. et al. Screening and engineering of high-activity promoter elements through transcriptomics and red fluorescent protein visualization in *Rhodobacter sphaeroides*. Synthetic Syst Biotechnol. 2021;6:335–42. 10.1016/j.synbio.2021.09.011. PMC8531756 34738044

[31] Lu W, Ye L, Lv X. et al. Identification and elimination of metabolic bottlenecks in the quinone modification pathway for enhanced coenzyme Q10 production in *Rhodobacter sphaeroides*. Metab Eng. 2015;29:208–16. 10.1016/j.ymben.2015.03.012. 25817210

[32] Qu Y, Su A, Li Y. et al. Manipulation of the regulatory genes *ppsR* and *prrA* in *Rhodobacter sphaeroides* enhances lycopene production. J Agric Food Chem. 2021;69:4134–43. 10.1021/acs.jafc.0c08158. 33813825

[33] He X, Wang D, Liu J. et al. Engineering the methylerythritol phosphate pathway and using a temporal promoter for enhanced lycopene production in *Rhodobacter sphaeroides* HY01. J Agric Food Chem. 2024;72:28040–7. 10.1021/acs.jafc.4c07848. 39626274

[34] Qiang S, Su AP, Li Y. et al. Elevated β-carotene synthesis by the engineered *Rhodobacter sphaeroides* with enhanced CrtY expression. J Agric Food Chem. 2019;67:9560–8. 10.1021/acs.jafc.9b02597. 31368704

[35] Zhang Y, Wang X, Yuan J. et al. Multidimensional engineering of *Rhodobacter sphaeroides* for enhanced photo-fermentative hydrogen production. Chem Eng J. 2024;488:150852. 10.1016/j.cej.2024.150852.

[36] Li R, Shi H, Zhao X. et al. Development and application of an efficient recombineering system for *Burkholderia glumae* and *Burkholderia plantarii*. Microb Biotechnol. 2021;14:1809–26. 10.1111/1751-7915.13840. 34191386 PMC8313284

[37] Yu LP, Yan X, Zhang X. et al. Biosynthesis of functional polyhydroxyalkanoates by engineered *Halomonas bluephagenesis*. Metab Eng. 2020;59:119–30. 10.1016/j.ymben.2020.02.005. 32119929

[38] Chen X, Yu L, Qiao G. et al. Reprogramming *Halomonas* for industrial production of chemicals. J Ind Microbiol Biotechnol. 2018;45:545–54. 10.1007/s10295-018-2055-z. 29948194

[39] Xu T, Chen J, Mitra R. et al. Deficiency of exopolysaccharides and O-antigen makes *Halomonas bluephagenesis* self-flocculating and amenable to electrotransformation. Commun Biol. 2022;5:623. 10.1038/s42003-022-03570-y. 35750760 PMC9232590

[40] Sistrom WR. A requirement for sodium in the growth of *Rhodopseudomonas spheroides*. J Gen Microbiol. 1960;22:778–85. 10.1099/00221287-22-3-778. 14447230

[41] Donohue TJ, Kaplan S. Genetic techniques in *Rhodospirillaceae*. Methods Enzymol. 1991;204:459–85. 1658568 10.1016/0076-6879(91)04024-i

[42] Altschul SF, Madden TL, Schäffer AA. et al. Gapped BLAST and PSI-BLAST: a new generation of protein database search programs. Nucleic Acids Res. 1997;25:3389–402. 10.1093/nar/25.17.3389. 9254694 PMC146917

[43] Sievers F, Higgins DG. Clustal Omega for making accurate alignments of many protein sequences. Protein Sci. 2018;27:135–45. 10.1002/pro.3290. 28884485 PMC5734385

[44] Tamura K, Stecher G, Kumar S. MEGA11: Molecular Evolutionary Genetics Analysis version 11. Mol Biol Evol. 2021;38:3022–7. 10.1093/molbev/msab120. 33892491 PMC8233496

[45] Nguyen LT, Schmidt HA, von Haeseler A. et al. IQ-TREE: a fast and effective stochastic algorithm for estimating maximum-likelihood phylogenies. Mol Biol Evol. 2015;32:268–74. 10.1093/molbev/msu300. 25371430 PMC4271533

[46] Hoang DT, Chernomor O, von Haeseler A. et al. UFBoot2: improving the ultrafast bootstrap approximation. Mol Biol Evol. 2018;35:518–22. 10.1093/molbev/msx281. 29077904 PMC5850222

[47] Kalyaanamoorthy S, Minh BQ, Wong TKF. et al. ModelFinder: fast model selection for accurate phylogenetic estimates. Nat Methods. 2017;14:587–9. 10.1038/nmeth.4285. 28481363 PMC5453245

[48] Maddison WP, Maddison DR. Mesquite: a modular system for evolutionary analysis. Version 3.81. 2023. https://www.mesquiteproject.org. (20 April 2023, date last accessed).

[49] Letunic I, Bork P. Interactive Tree of Life (iTOL) v6: recent updates to the phylogenetic tree display and annotation tool. Nucleic Acids Res. 2024;52:W78–82. 10.1093/nar/gkae268. 38613393 PMC11223838

[50] Abramson J, Adler J, Dunger J. et al. Accurate structure prediction of biomolecular interactions with AlphaFold 3. Nature. 2024;630:493–500. 10.1038/s41586-024-07487-w. 38718835 PMC11168924

[51] Pettersen EF, Goddard TD, Huang CC. et al. UCSF ChimeraX: structure visualization for researchers, educators, and developers. Protein Sci. 2021;30:70–82. 10.1002/pro.3943. 32881101 PMC7737788

[52] Henikoff S, Henikoff JG. Amino acid substitution matrices from protein blocks. Proc Natl Acad Sci USA. 1992;89:10915–9. 10.1073/pnas.89.22.10915. 1438297 PMC50453

[53] Xin Y, Guo T, Mu Y. et al. Identification and functional analysis of potential prophage-derived recombinases for genome editing in *Lactobacillus casei*. FEMS Microbiol Lett. 2017;364:fnx243. 10.1093/femsle/fnx243. 29145601

[54] Sun Z, Deng A, Hu T. et al. A high-efficiency recombineering system with PCR-based ssDNA in *Bacillus subtilis* mediated by the native phage recombinase GP35. Appl Microbiol Biotechnol. 2015;99:5151–62. 10.1007/s00253-015-6485-5. 25750031

[55] van Kessel JC, Hatfull GF. Efficient point mutagenesis in mycobacteria using single-stranded DNA recombineering: characterization of antimycobacterial drug targets. Mol Microbiol. 2008;67:1094–107. 18221264 10.1111/j.1365-2958.2008.06109.x

[56] Ind AC, Porter SL, Brown MT. et al. Inducible-expression plasmid for *Rhodobacter sphaeroides* and *Paracoccus denitrificans*. Appl Environ Microb. 2009;75:6613–5. 10.1128/AEM.01587-09. PMC2765152 19684165

[57] Huang X, Sun Y, Liu S. et al. Recombineering using RecET-like recombinases from *Xenorhabdus* and its application in mining of natural products. Appl Microbiol Biotechnol. 2022;106:7857–66. 10.1007/s00253-022-12258-6. 36326838

[58] Zakharova K, Liu M, Greenwald JR. et al. Structural basis for the interaction of Redβ single-strand annealing protein with *Escherichia coli* single-stranded DNA-binding protein. J Mol Biol. 2024;436:168590. 10.1016/j.jmb.2024.168590. 38663547

[59] Eraso JM, Kaplan S. prrA, a putative response regulator involved in oxygen regulation of photosynthesis gene expression in *Rhodobacter sphaeroides*. J Bacteriol. 1994;176:32–43. 10.1128/jb.176.1.32-43.1994. 8282708 PMC205011

[60] Fitschen LJ, Newing TP, Johnston NP. et al. Half a century after their discovery: structural insights into exonuclease and annealase proteins catalyzing recombineering. Engineering Microbiology. 2024;4:100120. 10.1016/j.engmic.2023.100120. 39628787 PMC11611040

[61] Jiang X. Metabolic engineering of *Escherichia coli* and *Rhodobacter sphaeroides* for the production of coenzyme Q₁₀. Fujian Normal University. 2019.

[62] Cao P, Bracun L, Yamagata A. et al. Structural basis for the assembly and quinone transport mechanisms of the dimeric photosynthetic RC-LH1 supercomplex. Nat Commun. 2022;13:1977. 10.1038/s41467-022-29563-3. 35418573 PMC9007983

[63] Kaplan S. Photosynthesis genes and their expression in *Rhodobacter sphaeroides* 2.4.1: a tribute to my students and associates. Photosynth Res. 2002;73:95–108. 10.1023/A:1020437317471. 16245109

[64] Su A, Chi S, Li Y. et al. Metabolic redesign of *Rhodobacter sphaeroides* for lycopene production. J Agric Food Chem. 2018;66:5879–85. 10.1021/acs.jafc.8b00855. 29806774

[65] Mougiakos I, Orsi E, Ghiffary MR. et al. Efficient Cas9-based genome editing of *Rhodobacter sphaeroides* for metabolic engineering. Microb Cell Fact. 2019;18:204. 10.1186/s12934-019-1255-1. 31767004 PMC6876111

[66] Hall AN, Hall BW, Kinney KJ. et al. Tools for genetic engineering and gene expression control in *Novosphingobium aromaticivorans* and *Rhodobacter sphaeroides*. Appl Environ Microb. 2024;90:e0034824. 10.1128/aem.00348-24. PMC11497788 39324814

[67] Filsinger GT, Mychack A, Lyerly E. et al. A diverse single-stranded DNA-annealing protein library enables efficient genome editing across bacterial phyla. Proc Natl Acad Sci USA. 2025;122:e2414342122. 10.1073/pnas.2414342122. 40258142 PMC12054835

[68] Subramaniam S, Erler A, Fu J. et al. DNA annealing by Redβ is insufficient for homologous recombination and the additional requirements involve intra- and inter-molecular interactions. Sci Rep. 2016;6:34525. 10.1038/srep34525. 27708411 PMC5052646

[69] Thresher RJ, Makhov AM, Hall SD. et al. Electron microscopic visualization of RecT protein and its complexes with DNA. J Mol Biol. 1995;254:364–71. 10.1006/jmbi.1995.0623. 7490755

[70] Thomason LC, Costantino N, Court DL. Examining a DNA replication requirement for Bacteriophage lambda Red- and Rac prophage RecET-promoted recombination in *Escherichia coli*. mBio. 2016;7:e01443–16. 10.1128/mBio.01443-16. 27624131 PMC5021808

[71] Caldwell BJ, Norris AS, Karbowski CF. et al. Structure of a RecT/Redbeta family recombinase in complex with a duplex intermediate of DNA annealing. Nat Commun. 2022;13:7855. 10.1038/s41467-022-35572-z. 36543802 PMC9772228

[72] Caldwell BJ, Zakharova E, Filsinger GT. et al. Crystal structure of the Redbeta C-terminal domain in complex with lambda Exonuclease reveals an unexpected homology with lambda Orf and an interaction with *Escherichia coli* single stranded DNA binding protein. Nucleic Acids Res. 2019;47:1950–63. 10.1093/nar/gky1309. 30624736 PMC6393309

[73] Kerr ID, Sivakolundu S, Li Z. et al. Crystallographic and NMR analyses of UvsW and UvsW.1 from bacteriophage T4. J Biol Chem. 2007;282:34392–400. 10.1074/jbc.M705900200. 17878153

[74] Bobay LM, Touchon M, Rocha EP. Manipulating or superseding host recombination functions: a dilemma that shapes phage evolvability. PLoS Genet. 2013;9:e1003825. 10.1371/journal.pgen.1003825. 24086157 PMC3784561

[75] O’Leary NA, Cox E, Holmes JB. et al. Exploring and retrieving sequence and metadata for species across the tree of life with NCBI Datasets. Sci Data. 2024;11:732. 10.1038/s41597-024-03571-y. 38969627 PMC11226681

[76] Corts A, Thomason LC, Costantino N. et al. Recombineering in non-model bacteria. Current Protocols. 2022;2:e605. 10.1002/cpz1.605. 36546891 PMC9793987

