# Supplementary material for: ETfinder: harnessing conserved C-terminal tails of single-stranded DNA-binding proteins for mining and engineering RecET systems in non-model microbial chassis
Source: Nucleic Acids Res. 2026 Aug 20;54(15):gkag818. doi: 10.1093/nar/gkag818 (PMC13490951; doi:10.1093/nar/gkag818)
Supplement: Supplementary file 1 [file gkag818_supplemental_files.zip › SI-revised-3.pdf]

Supplementary Information

ETfinder: harnessing conserved C-terminal tails of single-stranded DNA-binding proteins for mining and engineering RecET systems in non-model microbial chassis

Dongyuan Lv[1], Mindong Liang[1], Yiyang Gu[1], Xiangying Zhu[2], Hailong Wang[3], Menglei Xia[4], Jinkang Hao[4], Youyuan Li[1], Weishan Wang[5], Linquan Bai[6], Jiagao Cheng[1,2], Lixin Zhang[1], Gao-Yi Tan[1, *]

[1] State Key Laboratory of Bioreactor Engineering (SKLBE), and School of Biotechnology, East China University of Science and Technology (ECUST), Shanghai 200237, China
[2] Shanghai Key Laboratory of Chemical Biology, School of Pharmacy, East China University of Science and Technology, Shanghai 200237, China
[3] State Key Laboratory of Microbial Technology, School of Life Science, Shandong University, Jinan 250100, China
[4] Metabolism and Fermentation Process Control, College of Biotechnology, Ministry of Education, Tianjin University of Science and Technology, Tianjin 300457, China
[5] State Key Laboratory of Microbial Resources, Institute of Microbiology, Chinese Academy of Sciences, Beijing 100101, China
[6] State Key Laboratory of Microbial Metabolism and School of Life Sciences & Biotechnology, Shanghai Jiao Tong University, Shanghai 200240, China
To whom correspondence should be addressed. Tel/Fax: +86 21 64253020; Email: tangy@ecust.edu.cn

a

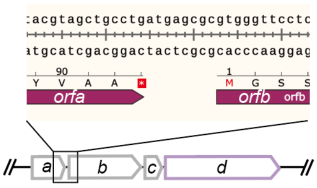

No.	Gene locus	Product/BLAST hit
orfa	DDV93_RS14075	YqaJ viral recombinase family protein
orfb	DDV93_RS14080	YqaJ viral recombinase family protein
orfc	DDV93_RS22590	hypothetical protein
orfd	DDV93_RS14090	recombinase RecT

b

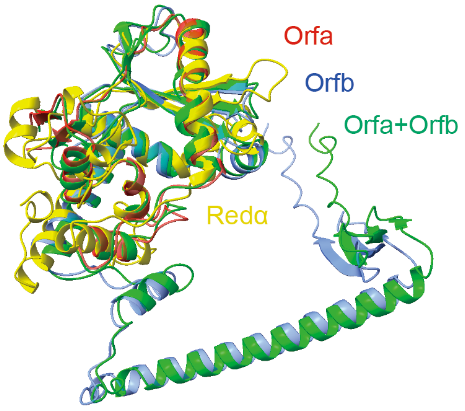

Figure S1. Putative disrupted *orf* of YqaJ homologs in a *Cereibacter johrii* strain. (a) Gene locus and corresponding products of the *recET* operon showing the disrupted *orfa* and *orfb*. a *recET* operon was found in *C. johrii* str. CDR-SL 7Cii SO_7654 (Accession: NZ_QMCC01000008). Detailed information for each open reading frame (ORF) is presented in the table. The putative *recE* (*orfa* and *orfb*) gene appears to be disrupted, as both Orfa and Orfb seem to be parts of a single YqaJ homolog that is interrupted by stop codons. Our experimental results also indicate that the native operon does not facilitate editing in *R. sphaeroides* (data not shown). (b) Structural overlay of Orfa, Orfb, fused Orfa + Orfb and Redα. Using AlphaFold2, we predicted the structures of Orfa (red, semi-transparent), Orfb (blue, semi-transparent), Orfa + Orfb (green), and Redα (yellow). The fusion of Orfa and Orfb, achieved by simply removing the stop codon, reveals that they can form a complete YqaJ domain structure. Therefore, we hypothesize that this is a non-functional RecET system due to disruption by mutations.

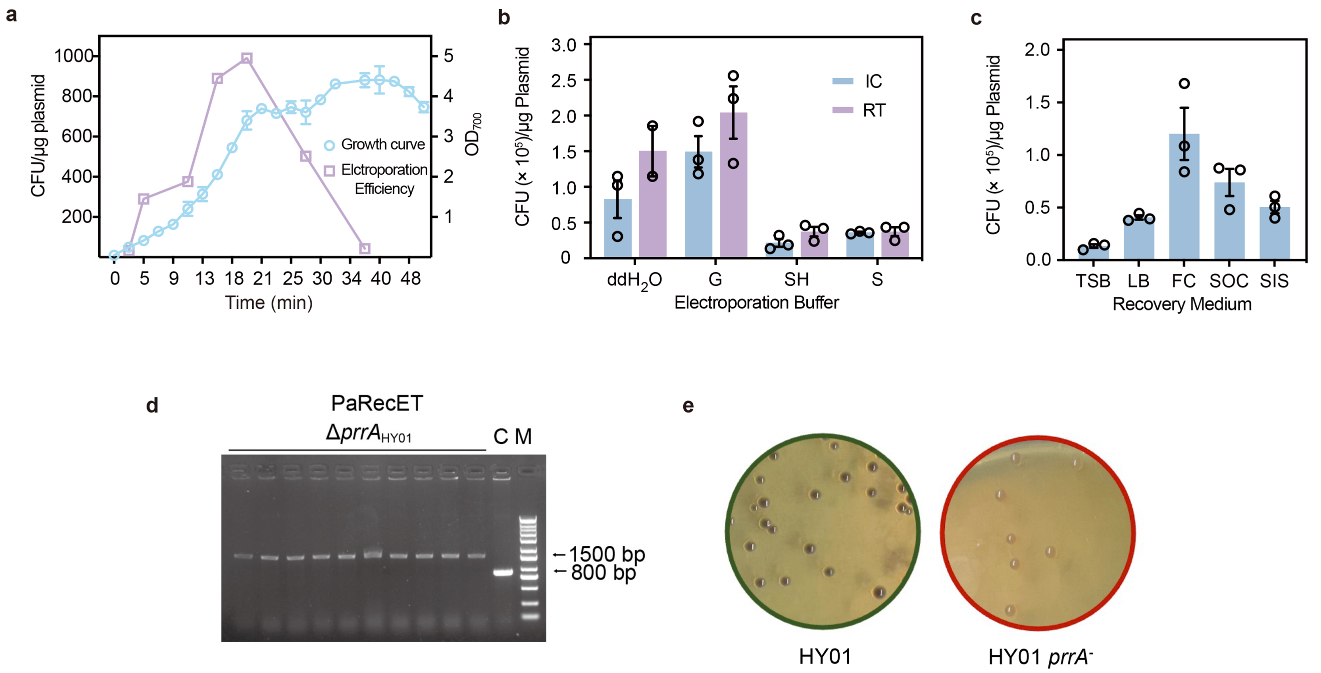

Figure S2. Electroporation optimization and PCR verification of PaRecET function via genome editing in *R. sphaeroides* HY01. **(a)** The electroporation efficiency of HY01 at different growth stages. At 7 time points (2, 5, 11, 15, 27, 36 h) covering different growth stages of HY01 cells, electrocompetent cells were prepared, transformed with fixed concentration plasmids, and transformation efficiency was characterized through CFU counting. **(b)** Effects of electroporation buffer and temperature on the electroporation efficiency in HY01. G: 10% glycerol; SH: 10% glycerol, 1 mM Hepes buffer; S: 10% sucrose; IC: Incubation on ice; RT: Room temperature. **(c)** Effects of different recovery media on the electroporation efficiency in HY01. TSB, tryptone soya broth; FC, fermentation culture; SIS, Sistrom's minimal medium. **(d)** Δ*prrA*$_{HY01}$ indicates knockout of the *prrA* locus of the HY01 genome (see main text). C is the negative control. **(e)** The knockout of the *prrA* gene causes HY01 to shift from a green to a pale pink phenotype.

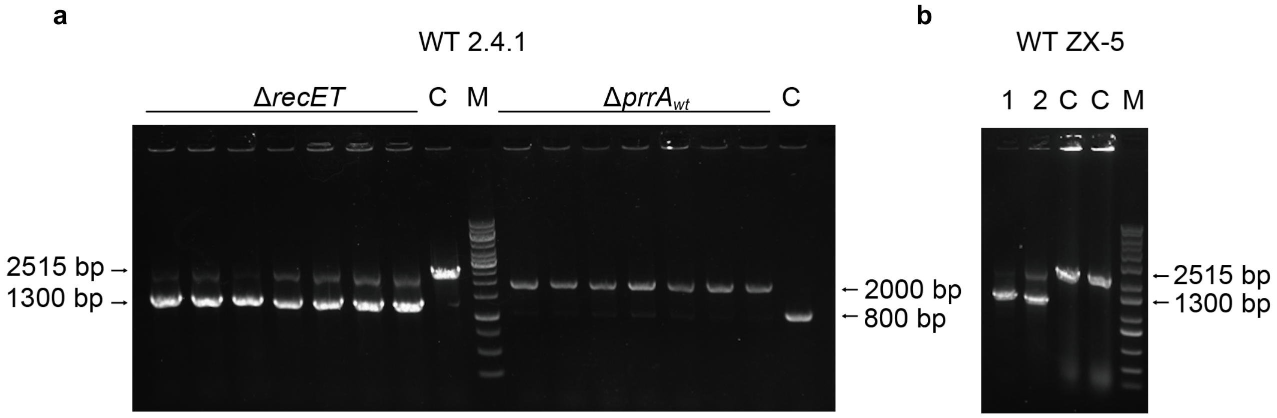

Figure S3. PCR validation of PaRecET function in *R. sphaeroides* wild-type strain 2.4.1 and ZX-5. **(a)** Δ*recET* represents plasmid editing, Δ*prrA* indicates knockout of the *prrA* locus of the 2.4.1 genome (see main text). C is the negative control. **(b)** Plasmid editing validation in ZX-5. Lanes 1 and 2 show positive results, and C represents the two replicates of negative control.

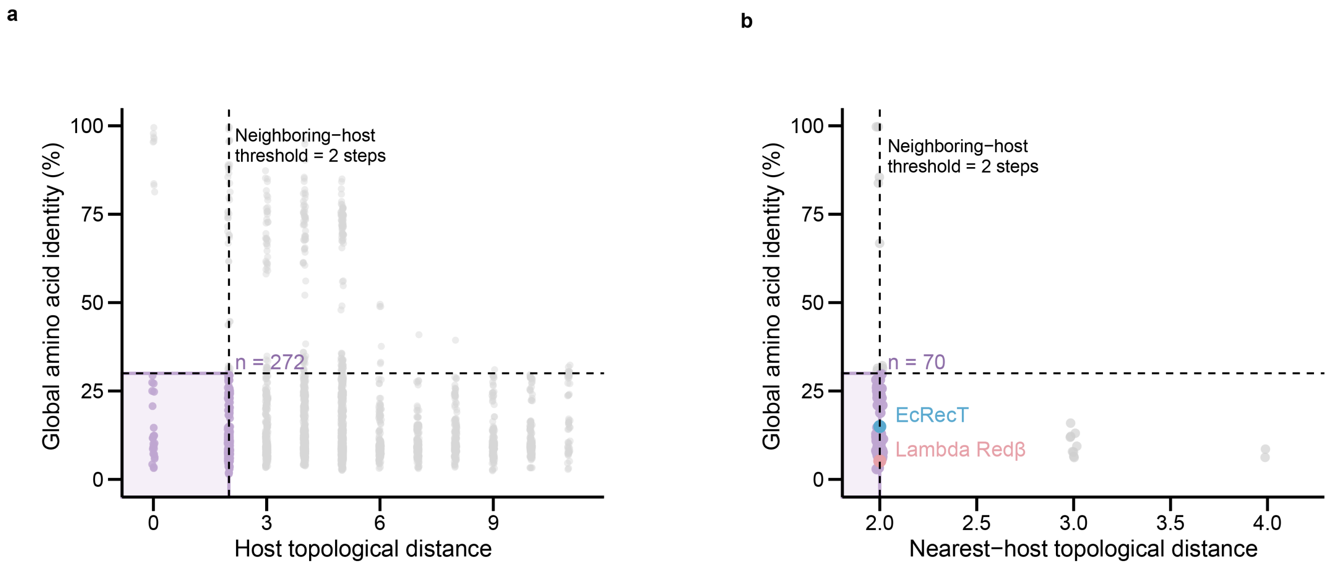

Figure S4. Host-tree topological proximity is poorly coupled to RecT homolog similarity in the *Halomonas* case. Host topological distance was compared with global amino acid identity of RecT homologs in the *Halomonas* dataset, using the host tree and protein sequences shown in Fig. 5b and Fig. 5c. Identity was calculated from the MAFFT alignment as described in the Supplementary Methods. Because the host tree has unit branch lengths, the x axis represents topological rather than calibrated evolutionary distance. Dashed lines indicate the neighboring-host threshold (topological distance ≤2) and the divergent-protein threshold (<30% identity). Grey points show background comparisons; light-purple points satisfy both thresholds, with n indicating their number. **(a)** All 2,628 pairwise comparisons among 73 proteins from 53 host groups (highlighted n = 272). Proteins assigned to the same collapsed host group had a host distance of 0 but retained their pairwise sequence identities. The association was weak (Spearman's ρ = −0.139). **(b)** Nearest-host subset comprising 91 protein comparisons (highlighted n = 70); all protein combinations were retained when a host group contained multiple homologs. The association remained weak (Spearman's ρ = −0.192). EcRecT and Lambda Red Beta are shown as reference overlays and were excluded from background counts, cutoff definitions, highlighted counts, and Spearman correlations. Because pairwise comparisons are non-independent, Spearman's ρ is interpreted as a descriptive effect size. Together with Fig. S6, these results suggest that RecT homolog similarity does not simply track host relatedness in the *Halomonas* case, as identical or topologically neighboring host assignments can contain highly divergent homologs.

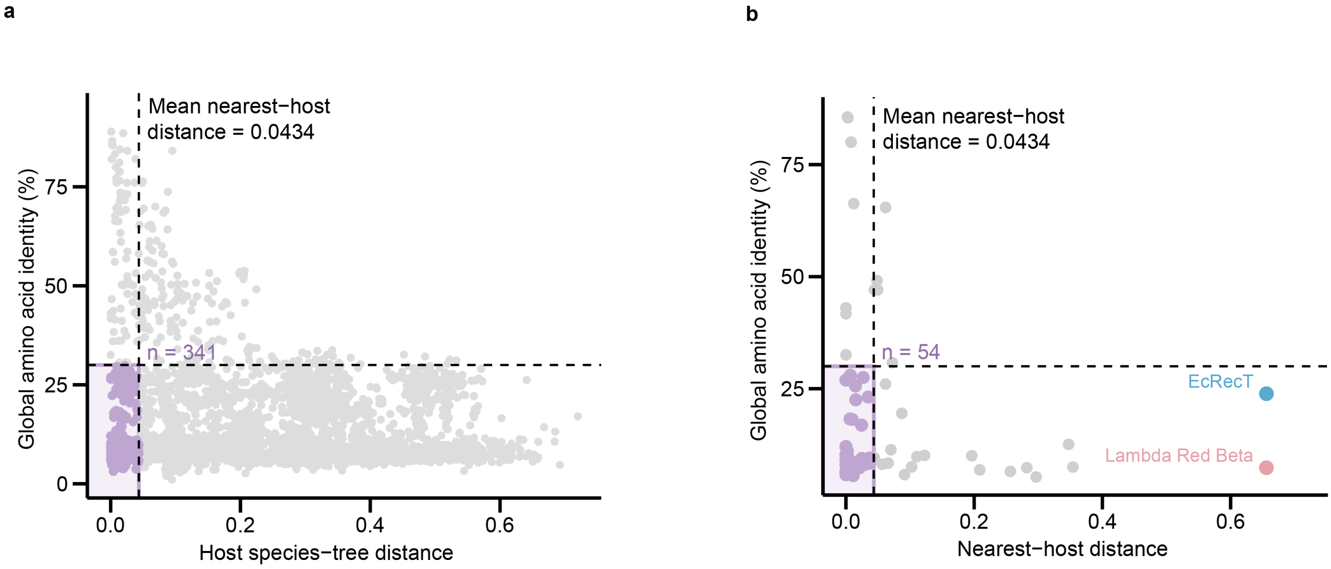

Figure S5. RecT homolog divergence among close host relatives in the *R. sphaeroides* case study. Host phylogenetic distance was compared with RecT homolog amino acid identity for matched RecT-bearing hosts in the *R. sphaeroides*-centered dataset. Data were derived from the host 16S rRNA tree and RecT homolog sequence set shown in Fig. 2b and Fig. 2c. The dashed vertical line indicates the mean nearest-host distance among the 91 non-reference host-RecT entries (0.0434), and the dashed horizontal line indicates 30% amino acid identity. Purple points indicate close-host comparisons with divergent RecT homologs, defined as host distance ≤ 0.0434 and RecT identity <30%; grey points indicate all other non-reference comparisons. **(a)** All pairwise comparisons among the 91 non-reference host-RecT entries, yielding 4,095 non-redundant host-RecT pairwise comparisons. Among them, 341 pairs were within the mean nearest-host distance threshold but had <30% RecT amino acid identity. Across all comparisons, host distance was only weakly associated with RecT identity (Spearman's $\rho = -0.085$). **(b)** Nearest-host comparisons for the 91 non-reference host-RecT entries. Each point represents one host and its closest matched non-reference host in the 16S rRNA tree. Of these 91 nearest-host comparisons, 54 had <30% RecT amino acid identity despite being within the mean nearest-host distance threshold. Among nearest-host comparisons, host distance and RecT identity showed essentially no rank correlation (Spearman's $\rho = 0.008$). EcRecT and Lambda Redβ are shown in blue and pale red, respectively, as reference overlays. Together, these analyses show that, within the *R. sphaeroides*-centered RecT-bearing host set, RecT protein similarity is frequently discordant with 16S rRNA relatedness. Even among close host relatives in this dataset, RecT proteins can be highly divergent, indicating that these systems do not simply follow vertical host evolution and underscoring the broader diversity of RecT/Bet-like recombination systems described in the main text.

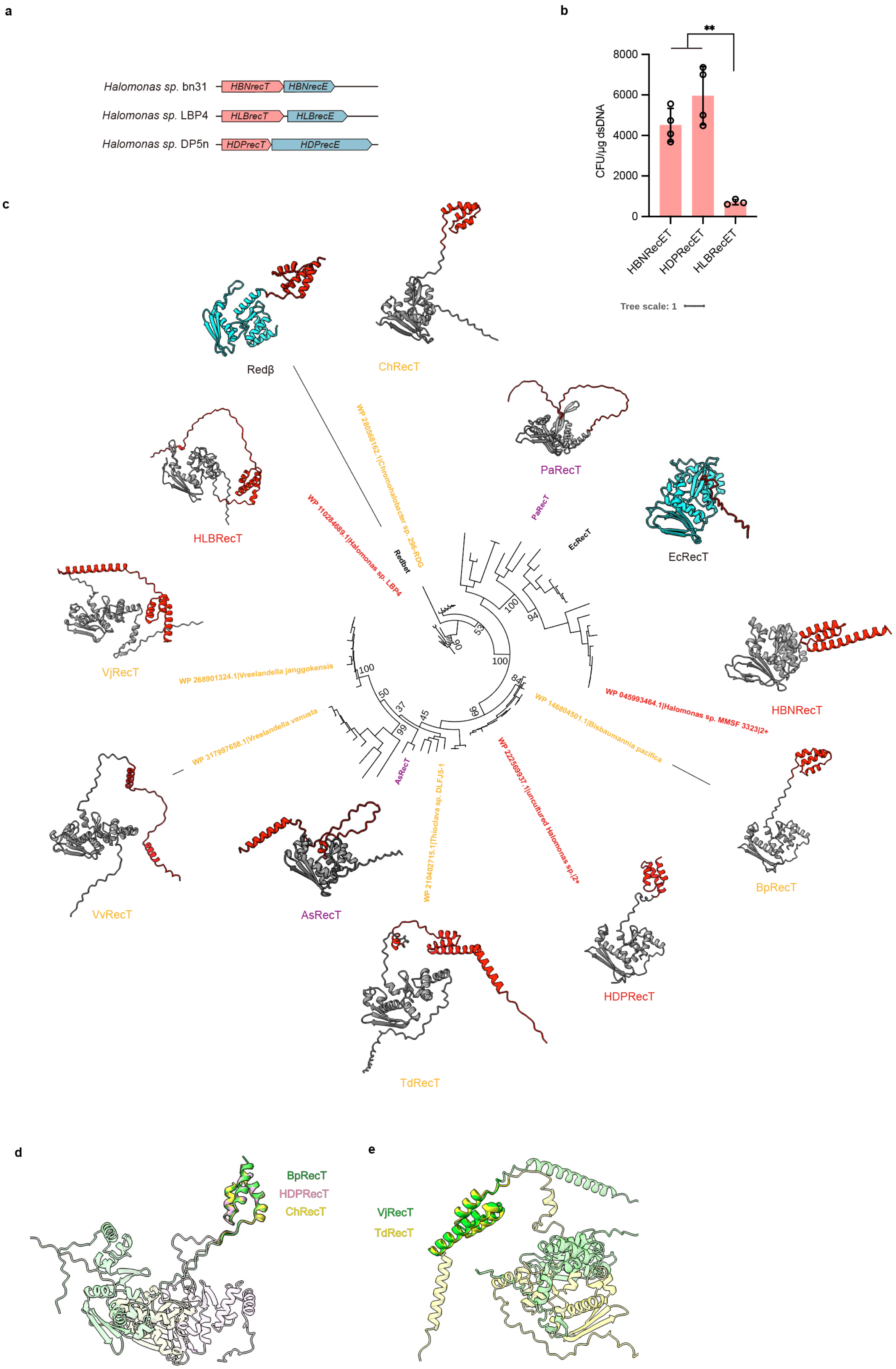
a
Halomonas sp. bn31
HBNrecT
HBNrecE
Halomonas sp. LBP4
HLBrecT
HLBrecE
Halomonas sp. DP5n
HDPrecT
HDPrecE
b
**
8000
6000
4000
2000
0
CFU/μg dsDNA
HBNRecET
HDPRecET
HLBRecET
Tree scale: 1
c
Redβ
ChRecT
PaRecT
EcRecT
HLBRecT
VjRecT
HBNRecT
BpRecT
HDPRecT
TdRecT
AsRecT
VvRecT
d
BpRecT
HDPRecT
ChRecT
e
VjRecT
TdRecT

Figure S6. Genomic context, editing performance, phylogenetic distribution and C-terminal structural comparison of RecT homologs in the *Halomonas* case study. **(a)** Schematic diagrams of the native RecT gene loci from three *Halomonas sp.* strains. Each native RecT is shown together with its cognate RecE (or RecE-like) partner in their genomic arrangement. **(b)** DsDNA recombineering efficiencies of the three native RecT homologs in the *Halomonas* host. Bars show mean CFU μg^{-1} dsDNA, with individual replicates overlaid as points. Error bars indicate standard deviation (SD), and individual biological replicates are overlaid as open circles. Statistical significance was assessed using an unpaired two-tailed Student's *t*-test ($P < 0.01$). **(c)** Phylogeny of RecT homologs identified in *Halomonas* and related taxa. Native *Halomonas* RecTs are highlighted in red, and non-native candidates in yellow. For each representative, the terminal folded domain at the extreme C terminus is shown in red. **(d)** Structural superposition of the C-terminal domains of BpRecT, HDPRecT, and ChRecT. **(e)** Structural superposition of the C-terminal domains of VjRecT and TdRecT, illustrating the shared C-terminal fold among these distant homologs.

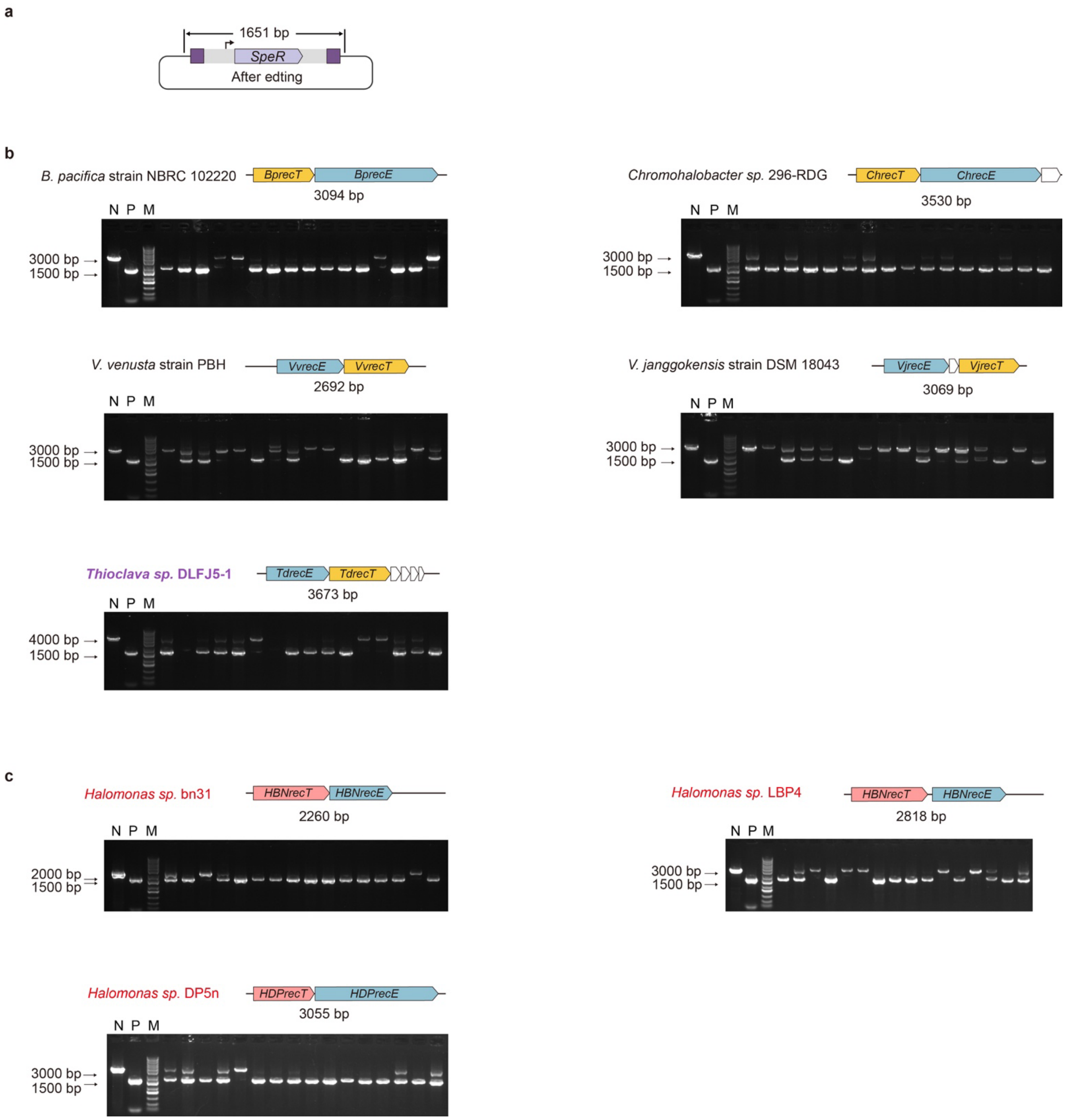

Figure S7. Validation of dsDNA recombineering using non-native and native RecET systems across multiple hosts. **(a)** Schematic of the diagnostic PCR amplicon obtained after successful replacement of the *speR* locus by the dsDNA editing cassette. The expected product length after integration is 1651 bp. **(b)** PCR verification of dsDNA recombineering mediated by non-native RecET systems. For each donor species, the corresponding *recE–recT* genomic arrangement is shown above the gel. Lanes represent negative control (N), positive control (P), and marker (M). Successful edits yield the predicted integrated product for each tested RecET pair. **(c)** PCR verification of dsDNA recombineering using native RecET systems from *Halomonas* strains. Gene locus diagrams are shown above each gel. Native RecETs also generate the expected edited amplicon, confirming functional dsDNA recombination activity.

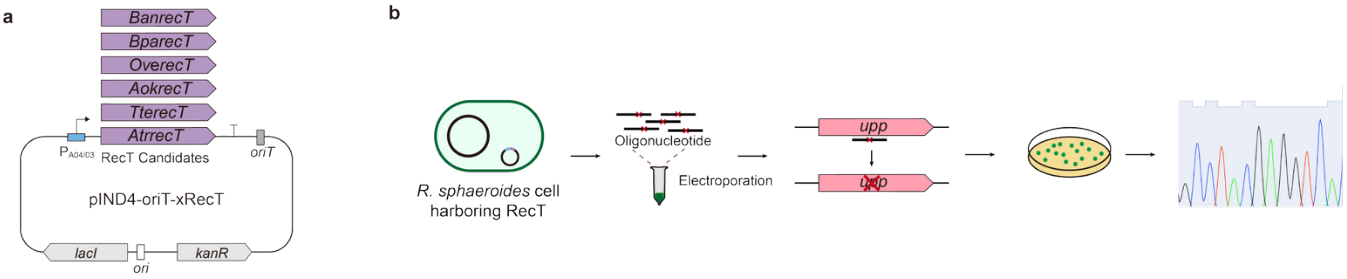

c

Cand.	Pos./Total (n ≥ 2)
PaRecT	0/24
NbRecT	0/24
SpRecT	0/24
AoRecT	0/15
AsRecT	0/15
OveRecT	9/21
AtrRecT	25/33
AokRecT	0/22
TteRecT	0/34
BanRecT	0/25
BpaRecT	0/24

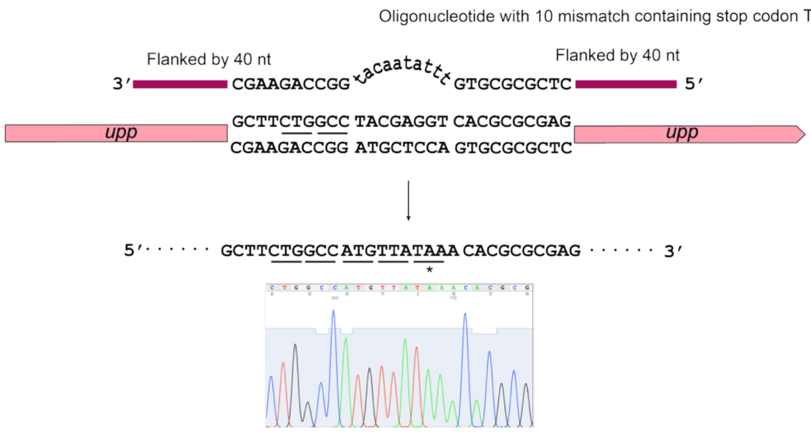

Figure S8. SsDNA editing assay for RecT candidates identified in a second round of SSB-constrained mining. **(a)** Schematic of the pIND4-oriT-xRecT expression constructs used to evaluate structurally Redβ-like RecT candidates (BanRecT, BpaRecT, OveRecT, AokRecT, TteRecT and AtrRecT) in *R. sphaeroides*. RecT expression is driven by the $P_{A1/O4/O3}$ promoter. **(b)** Workflow of the ssDNA editing assay. *R. sphaeroides* cells expressing individual RecT candidates were electroporated with a 40-nt–flanked oligonucleotide containing a central 10-nt mismatch window and an introduced TAA stop codon targeting the *upp* gene. The experimental procedures were performed as described for the dsDNA recombineering assays (see Materials and Methods), except that a single-stranded oligonucleotide was used in place of the linear dsDNA donor. Mutants were selected on 5-fluorouracil plates and subjected to Sanger sequencing. **(c)** Summary of ssDNA editing outcomes for the tested RecT candidates. Candidates above the dashed line are RecTs previously evaluated in the first-round dsDNA editing assay and were expressed using the same construct design as in Fig. S4a. Candidates below the dashed line are RecTs with Redβ-like C-terminal architectures selected for additional ssDNA editing tests. “Pos./Total” denotes the number of confirmed targeted edits among all sequenced colonies. Only OveRecT and AtrRecT produced the designed ssDNA edits, whereas no confirmed targeted mutations were detected for the remaining candidates under the tested conditions. **(d)** Design of the oligonucleotide used for targeted mutagenesis of *upp* and representative Sanger sequencing trace confirming incorporation of the designed nucleotide substitutions. The oligonucleotide contains a 10-nt mismatch region carrying the intended mutations and is flanked by 40-nt homology arms on both sides.

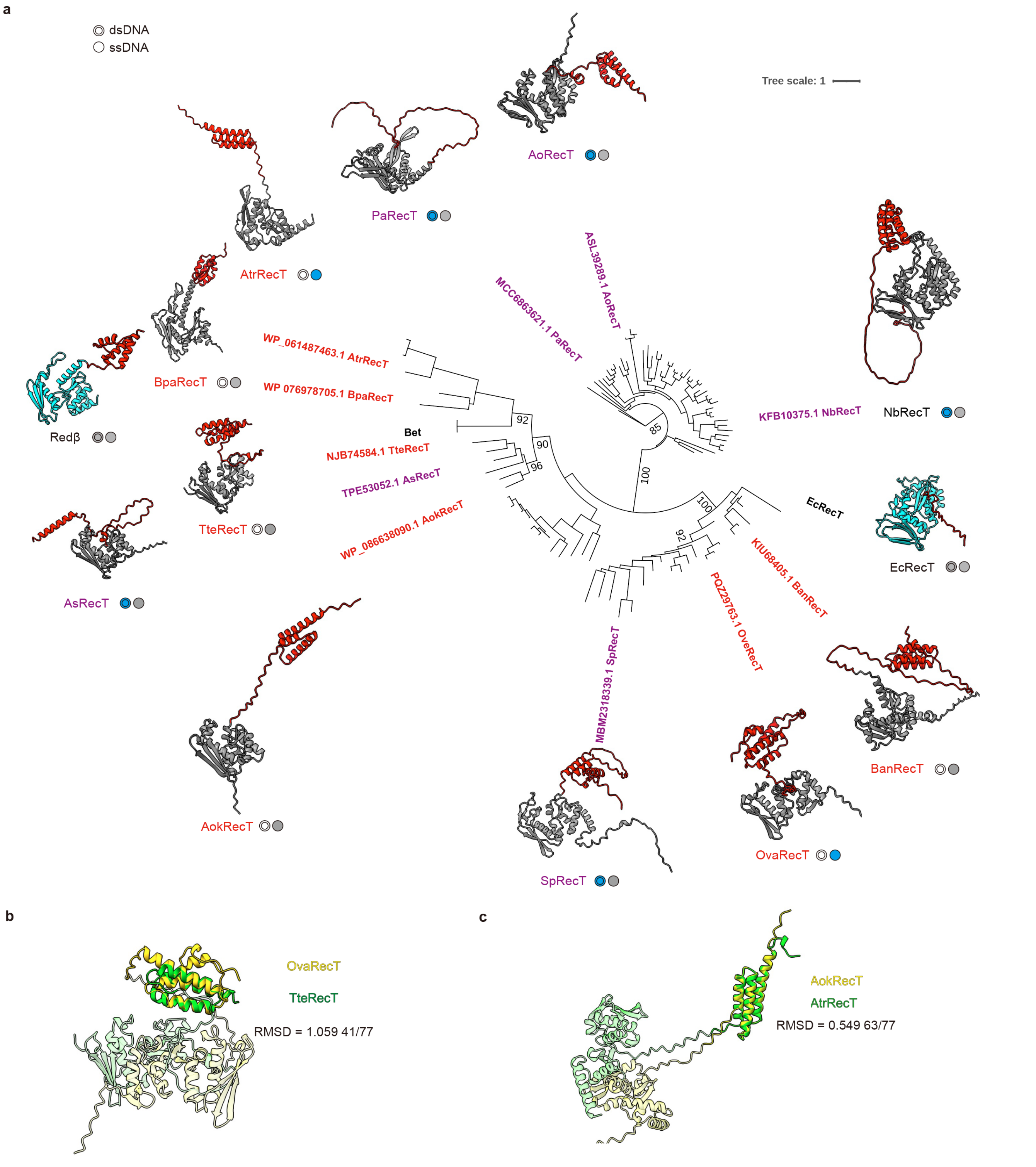

Figure S9. Predicted structures of RecT homologs tested in the *R. sphaeroides* case study and their correspondence to the reconstructed RecT phylogeny. **(a)** Phylogeny reconstructed from the RecT set used in Fig. 2c supplemented with an additional round of candidate mining. RecT homologs recovered in the second search are highlighted in bold red, whereas those from the initial search are highlighted in bold purple. For each representative, the terminal folded domain at the extreme C terminus is shown in red. Circular markers indicate recombination activity: double-line circles denote dsDNA recombination, single-line circles denote ssDNA recombination. Blue marks indicate experimentally validated activity, grey marks indicate no detectable activity, and white marks indicate untested proteins. **(b)** Structural superposition of the C-terminal domains of OvaRecT and TteRecT. The two structures exhibit a closely matching helical fold, with 41 out of 77 aligned residues overlapping in space (RMSD = 1.059 Å). **(c)** Structural superposition of the C-terminal domains of AokRecT and AtrRecT. These two RecT variants also show a high degree of structural similarity in their terminal helical region, with 63 out of 77 aligned residues overlapping (RMSD = 0.549 Å).

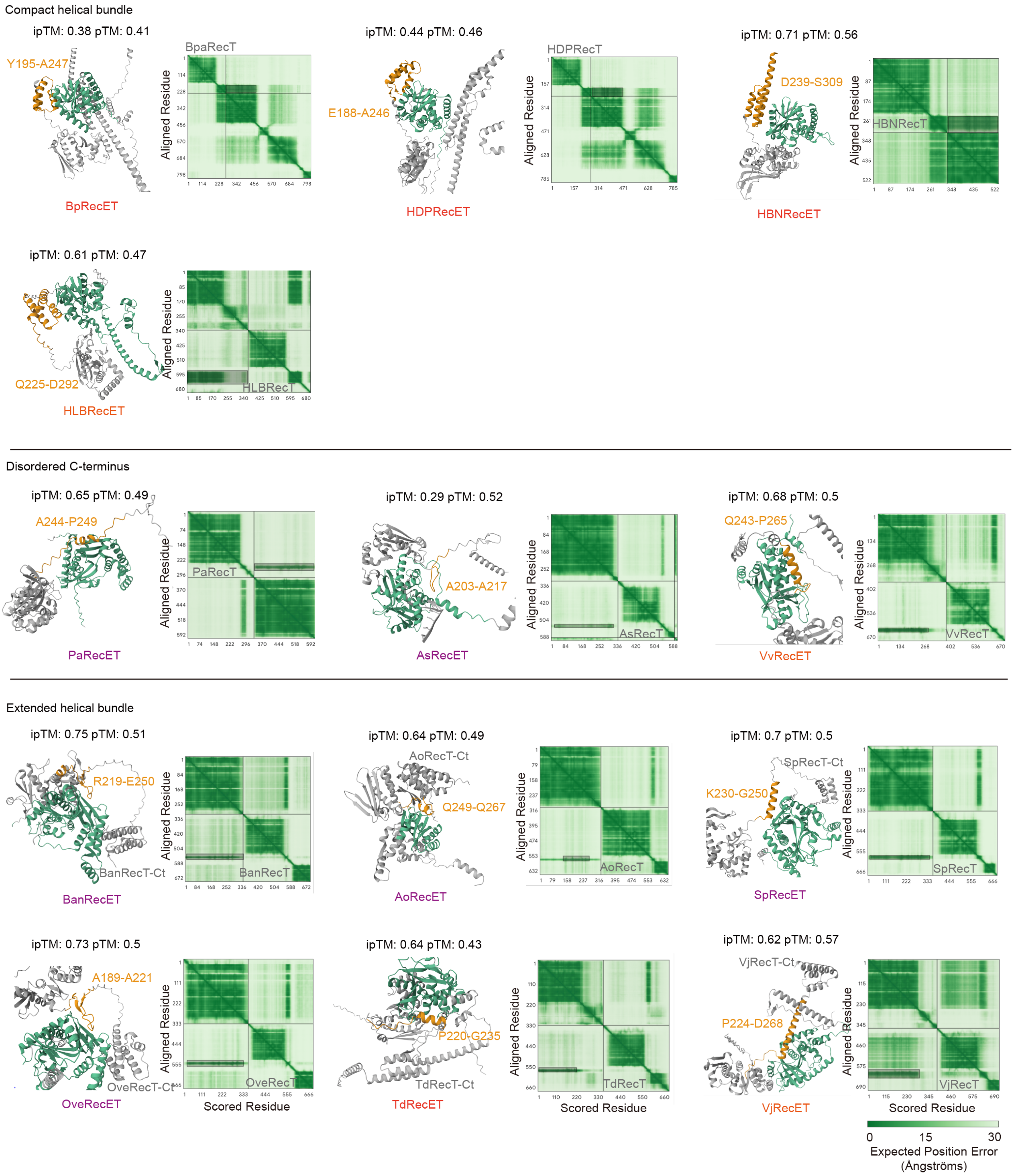

Figure S10. AF3 predictions of full-length RecET complexes reveal diverse RecT C-terminal architectures and putative RecE-facing surfaces. Full-length RecT–RecE complexes were predicted using the AlphaFold3 server for the indicated RecET pairs. The models are grouped according to the predicted architecture of the RecT C-terminal region: a compact helical bundle, disordered C terminus, and extended helical bundle. RecET names shown in purple indicate pairs experimentally tested in the *R. sphaeroides* case study, whereas names shown in red indicate pairs tested in the *Halomonas* case study. For each RecET pair, the predicted complex model is shown together with the corresponding full-length predicted aligned error (PAE) map. In the structural models, the full complex is shown in gray. Inter-chain regions selected from the PAE maps are mapped back onto the complex structures, with the selected RecT segment colored yellow and the corresponding RecE region colored green. The residue range of the highlighted RecT segment is indicated in yellow text next to each model. Darker green regions in the PAE maps indicate lower predicted positional error and higher confidence in the relative placement of residue pairs, whereas lighter regions indicate greater uncertainty. Off-diagonal signals between the RecT and RecE blocks indicate predicted inter-chain contacts or relative positioning between the two proteins. The ipTM and pTM scores are shown above each model where available.

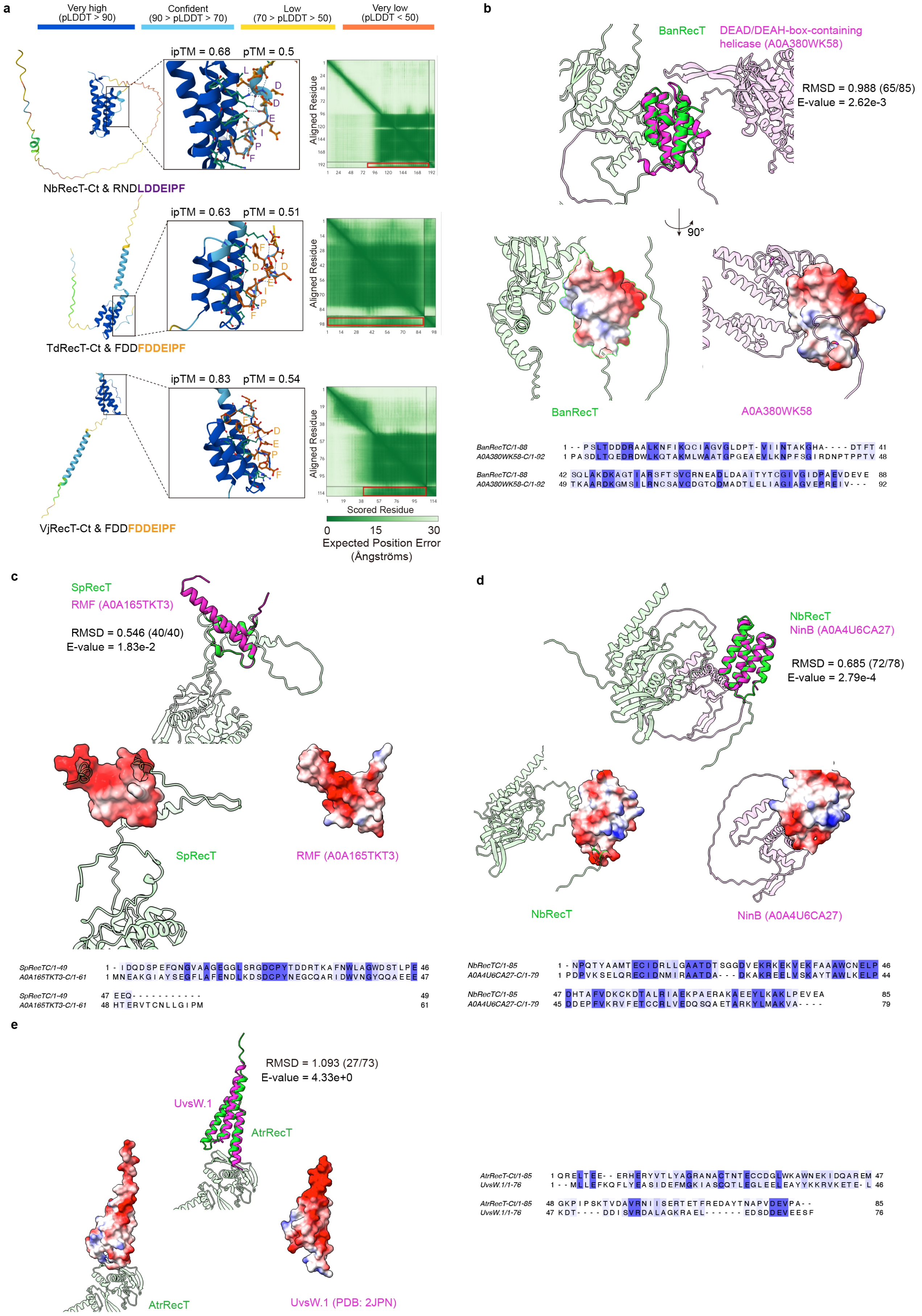
a
Very high (pLDDT > 90)
Confident (90 > pLDDT > 70)
Low (70 > pLDDT > 50)
Very low (pLDDT < 50)
ipTM = 0.68 pTM = 0.5
NbRecT-Ct & RNDLDDEIPF
ipTM = 0.63 pTM = 0.51
TdRecT-Ct & FDDFDDEIPF
ipTM = 0.83 pTM = 0.54
VjRecT-Ct & FDDFDDEIPF
Aligned Residue
Scored Residue
0 15 30
Expected Position Error (Ångströms)
b
BanRecT
DEAD/DEAH-box-containing helicase (A0A380WK58)
RMSD = 0.988 (65/85)
E-value = 2.62e-3
90°
BanRecT
A0A380WK58
BanRecTC/1-88 1 - - PSLTDDDRAALKNFIKQCIAGVGLDPT-VIINTAKGHA- - - - DTFT 41
A0A380WK58-C/1-92 1 PASDLTQEDRDWLKQTAKMLWAATGPGEAEVLKNPFSGIRDNPTPPTV 48
BanRecTC/1-88 42 SQLAKDKAGTIARSFTSVCRNEADLDAAITYTCGIVGIDPAEVDEVE 88
A0A380WK58-C/1-92 49 TKAARDKGMSILRNCSAVCDGTQDMADTLELIAGIAGVEPREIV- - - 92
c
SpRecT
RMF (A0A165TKT3)
RMSD = 0.546 (40/40)
E-value = 1.83e-2
SpRecT
RMF (A0A165TKT3)
SpRecTC/1-49 1 - IDQDSPEFQNGVAAGEGGLSRGDCPYTDDRTKAFNWLAGWDSTLPE 46
A0A165TKT3-C/1-61 1 MNEAKGIAYSEGFLAFENDLKDSDCPYNEGCQARIDWVNGYQQAEEE 47
SpRecTC/1-49 47 EEQ- - - - - - - - - - - - 49
A0A165TKT3-C/1-61 48 HTERVTCNLLGIPM 61
d
NbRecT
NinB (A0A4U6CA27)
RMSD = 0.685 (72/78)
E-value = 2.79e-4
NbRecT
NinB (A0A4U6CA27)
NbRecTC/1-85 1 - NPQTYAAMTECIDRLLGAATDTSGGDVEKRKEKVEKFAAAWCNELP 46
A0A4U6CA27-C/1-79 1 PDPVKSELQRECIDNMIRAATDA- - - DKAKREELVSKAYTAWLKELP 44
NbRecTC/1-85 47 DHTAFVDKCKDTALRIAEKPAERAKAEEYLKAKLPEVEA 85
A0A4U6CA27-C/1-79 45 DDEPFVKRVFETCCRLVEDQSQAETARKYLMAKVA- - - - 79
e
RMSD = 1.093 (27/73)
E-value = 4.33e+0
UvsW.1
AtrRecT
AtrRecT
UvsW.1 (PDB: 2JPN)
AtrRecT-Ct/1-85 1 QRELTEE- - ERHERYVTLYAGRANACTNTECCDGLWKAWNEKIDQAREM 47
UvsW.1/1-76 1 - - MLLEFKQFLYEASIDEFMGKIASCQTLEGLEELEAYYKKRVKETE- L 46
AtrRecT-Ct/1-85 48 GKPIPSKTVDAVRNIISERTETFREDAYTNAPVDEVPA- - 85
UvsW.1/1-76 47 KDT- - - - DDISVRDALAGKRAEL- - - - - - EDSDDEVEESF 76

Figure S11. Exploratory prediction of RecT C-terminal interactions with host SSB C-tails and structural reuse of RecT C-terminal extensions. **(a)** The *R. sphaeroides* host SSB C-tail was modeled as the C-terminal 10-aa peptide containing the LDDEIPF SSB-C7 motif, whereas the *H. bluephagenesis* host SSB C-tail was modeled as the C-terminal 10-aa peptide containing the FDDEIPF SSB-C7 motif. RecT C-terminal regions are colored according to predicted Local Distance Difference Test (pLDDT) scores, an AlphaFold per-residue confidence metric that reflects confidence in local structural geometry. Higher pLDDT values indicate greater confidence in the predicted local structure, whereas lower values indicate greater structural uncertainty and may be associated with flexible or disordered regions. The confidence scale is shown above. SSB C-tail peptides are shown as sticks in the zoomed views, and the SSB-C7 motifs are labeled in orange. The corresponding predicted aligned error (PAE) maps are shown on the right, where lower PAE values indicate greater confidence in the relative positioning of the corresponding residue pairs. Red boxes indicate the inter-chain regions used to inspect the predicted relative positioning of the RecT C-terminal region and SSB-Ct peptide. Mean ipTM and pTM scores across three predictions generated with different random seeds are indicated above each model. **(b–e)** Structural comparisons between RecT C-terminal extensions and representative Foldseek hits. RecT proteins are shown in green, and the matched structural homologues are shown in magenta. Superposed regions are highlighted in bright green and magenta. Electrostatic surface views are shown below the structural alignments to compare the surface properties of the matched regions. **(b)** BanRecT C-terminal extension aligned with a C-terminal region of a DEAD/DEAH-box-containing helicase (A0A380WK58). **(c)** SpRecT C-terminal extension aligned with an RMF-like fold (A0A165TKT3). **(d)** NbRecT C-terminal extension aligned with a NinB C-terminal region (A0A4U6CA27). **(e)** AtrRecT C-terminal extension aligned with the UvsW.1-associated region. E-values were obtained from Foldseek server searches. RMSD values were calculated in ChimeraX (v1.9) (1) after structural superposition; the numbers in parentheses indicate the number of pruned atom pairs used for the reported RMSD relative to the total number of selected atom pairs. Sequence alignments of the structurally matched regions were generated using ClustalW (https://www.ebi.ac.uk/jdispatcher/msa/clustalo, accessed 16 July 2026) and visualized in Jalview (v2.3.11.0) (2) with identity coloring and conservation at a 30% threshold. These structural similarities do not establish direct homology or imply that the RecT extensions perform the same functions as the matched proteins but suggest that RecT C-terminal extensions can adopt diverse helical scaffolds resembling modules found in other nucleic-acid-associated proteins.

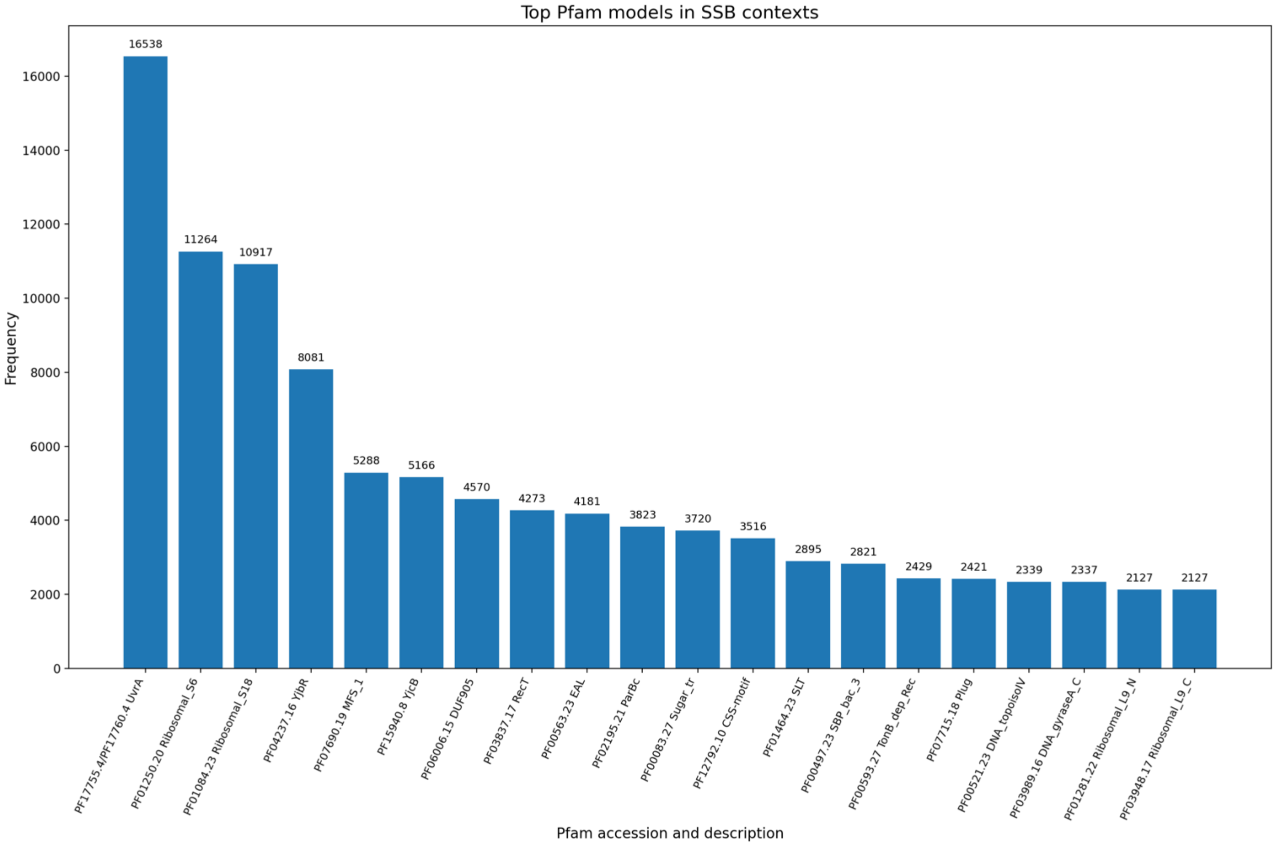

Figure S12. Pfam domain frequencies in the reference SSB context map. Bar plot showing the most frequent Pfam models identified in the reference SSB context map. The x-axis lists Pfam accessions and domain descriptions, and the y-axis indicates their frequency across all retrieved SSB genomic contexts. These counts represent the prevalence of co-occurring protein families surrounding SSB genes in the reference database.

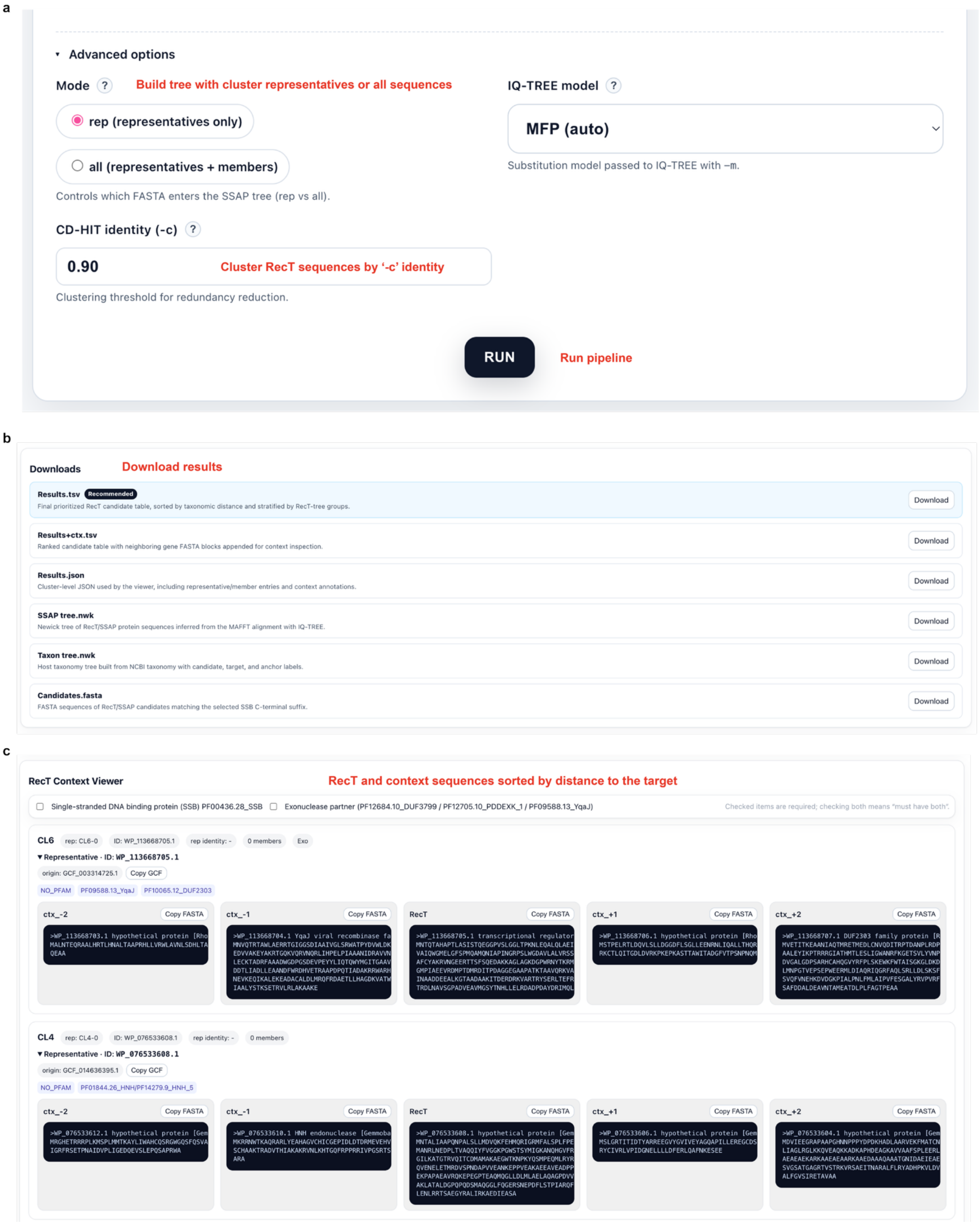
a
Advanced options
Mode
Build tree with cluster representatives or all sequences
rep (representatives only)
all (representatives + members)
Controls which FASTA enters the SSAP tree (rep vs all).
IQ-TREE model
MFP (auto)
Substitution model passed to IQ-TREE with -m.
CD-HIT identity (-c)
0.90
Cluster RecT sequences by '-c' identity
Clustering threshold for redundancy reduction.
RUN
Run pipeline
b
Downloads
Download results
Results.tsv
Recommended
Final prioritized RecT candidate table, sorted by taxonomic distance and stratified by RecT-tree groups.
Download
Results+ctx.tsv
Ranked candidate table with neighboring gene FASTA blocks appended for context inspection.
Download
Results.json
Cluster-level JSON used by the viewer, including representative/member entries and context annotations.
Download
SSAP tree.nwk
Newick tree of RecT/SSAP protein sequences inferred from the MAFFT alignment with IQ-TREE.
Download
Taxon tree.nwk
Host taxonomy tree built from NCBI taxonomy with candidate, target, and anchor labels.
Download
Candidates.fasta
FASTA sequences of RecT/SSAP candidates matching the selected SSB C-terminal suffix.
Download
c
RecT Context Viewer
RecT and context sequences sorted by distance to the target
Single-stranded DNA binding protein (SSB) PF00436.28_SSB
Exonuclease partner (PF12684.10_DUF3799 / PF12705.10_PDDEXK_1 / PF09588.13_YqaJ)
Checked items are required; checking both means "must have both".
CL6
rep: CL6-0
ID: WP_113668705.1
rep identity: -
0 members
Exo
Representative · ID: WP_113668705.1
origin: GCF_003314725.1
Copy GCF
NO_PFAM
PF09588.13_YqaJ
PF10065.12_DUF2303
ctx_-2
Copy FASTA
ctx_-1
Copy FASTA
RecT
Copy FASTA
ctx_+1
Copy FASTA
ctx_+2
Copy FASTA
CL4
rep: CL4-0
ID: WP_076533608.1
rep identity: -
0 members
Representative · ID: WP_076533608.1
origin: GCF_014636395.1
Copy GCF
NO_PFAM
PF01844.26_HNH/PF14279.9_HNH_5
ctx_-2
Copy FASTA
ctx_-1
Copy FASTA
RecT
Copy FASTA
ctx_+1
Copy FASTA
ctx_+2
Copy FASTA

Figure S13. Additional views of the ETfinder local web interface. **(a)** Advanced input options in the ETfinder local web server. Users can choose whether the RecT phylogeny is constructed using cluster representatives only or all clustered sequences, select the IQ-TREE substitution model, adjust the CD-HIT sequence identity threshold for RecT clustering, and start the analysis pipeline. **(b)** Download panel of the result page. The recommended output table (Results.tsv) contains the prioritized RecT candidates, while additional downloadable files include context-augmented candidate tables, cluster-level JSON files, RecT and host taxonomic trees, and candidate FASTA sequences. **(c)** RecT context viewer. Candidate RecT–SSB neighborhoods are displayed in ranked order and can be filtered for adjacent single-stranded DNA binding proteins (SSBs) or exonuclease partners. For each representative candidate, neighboring genomic-context proteins and RecT sequences are shown with options to copy FASTA sequences for downstream analysis.

Table S1. Bacterial strains used in this study.

Strains	Feartures	Source/Ref
E. coli		
DH10b	F- *araD139* Δ*(araA-leu)7697* Δ*(lac)X74 galE15 galK16 galU hsdR2 relA1 rpsL150(strR) spoT1 deoR* Φ*80dlacZ*ΔM15 *endA1 nupG recA1 e14- mcrA* Δ*(mrr hsdRMS mcrBC) tonA*	Gibco BRL
S17-1	RP4-2(Km::Tn7,Tc::Mu-1) *pro-82 recA1 endA1 thiE1 hsdR17 creC510*	This lab
R. sphaeroides		
2.4.1	Wild-type strain	ATCC
ZX-5	Wild-type strain	ATCC
HY01	Industrial strain	This lab
HY01/pIND4-oriT-EcRecET	HY01 carrying plasmid pIEcRecET expressing $RecE_{555}$ and RecT from *E. coli*, original operon; Kan^r	This study
HY01/pIND4-oriT-EcRecET-RBS	HY01 carrying plasmid pIEcRecET-RBS expressing $RecE_{555}$ and RecT from *E. coli*, polycistronic, connected by *puhA* RBS; Kan^r	This study
HY01/pIND4-oriT-Redαβγ	HY01 carrying plasmid pIRedαβγ expressing Redα, Redβ, and Redγ from Lambda phage, original operon; Kan^r	This study
HY01/pIND4-oriT-Redαβγ-RBS	HY01 carrying plasmid pIRedαβγ-RBS expressing edα, Redβ, and Redγ from Lambda phage, polycistronic, connected by *puhA* RBS; Kan^r	This study
HY01/pIND4-oriT-PaRecET	HY01 carrying plasmid pIPaRecET expressing RecE and RecT homologs from *Paracoccaceae* bacterium isolate SJ630, original operon; Kan^r	This study
HY01/pIND4-oriT-SpRecET	HY01 carrying plasmid pISpRecET expressing RecE and RecT homologs from *Sulfitobacter pseudonitzschiae* str. SM26-85, original operon; Kan^r	This study
HY01/pIND4-oriT-AoRecET	HY01 carrying plasmid pIAoRecET expressing RecE and RecT homologs from *Acetobacter oryzifermentans str. dm*, original operon; Kan^r	This study
HY01/pIND4-oriT-AoRecET-RBS	HY01 carrying plasmid pIAoRecET-RBS expressing RecE and RecT homologs from *A. oryzifermentans str. dm*, polycistronic, connected by *puhA* RBS; Kan^r	This study
HY01/pIND4-oriT-NbRecET	HY01 carrying plasmid pINbRecET expressing RecE and RecT homologs from *Nitratireductor basaltis* str. UMTGB225, original operon; Kan^r	This study
HY01/pIND4-oriT-AsRecET	HY01 carrying plasmid pIAsRecET expressing RecE and RecT homologs from *Amaricoccus solimangrovi* str. HB172011, original operon; Kan^r	This study
HY01/pIND4-oriT-AsRecET-RBS	HY01 carrying plasmid pIAsRecET-RBS expressing RecE and RecT homologs from *A. solimangrovi* str. HB172011, polycistronic, connected by *puhA* RBS; Kan^r	This study
HY01/pIND4-oriT-BanRecT	HY01 carrying plasmid pIBanRecT expressing RecT homologs from *Brucella anthropic*; Kan^r	This study
HY01/pIND4-oriT-BpaRecT	HY01 carrying plasmid pIBpaRecT expressing RecT homologs from *Brevirhabdus pacifica*; Kan^r	This study
HY01/pIND4-oriT-OveRecT	HY01 carrying plasmid pIOveRecT expressing RecT homologs from *Ochrobactrum vermis*; Kan^r	This study
HY01/pIND4-oriT-AtrRecT	HY01 carrying plasmid pIAtrRecT expressing RecT homologs from *Acetobacter tropicalis*; Kan^r	This study

(Continued)

Strains	Feartures	Source/Ref
HY01/pIND4-oriT-AokRecT	HY01 carrying plasmid pIAokRecT expressing RecT homologs from *Acetobacter okinawensis*; Kan^r	This study
HY01/pIND4-oriT-TteRecT	HY01 carrying plasmid pITteRecT expressing RecT homologs from *Thalassospira tepidiphila*; Kan^r	This study
HY01/pIND4-oriT-PaRecETtm	HY01 carrying plasmid pIPaRecETtm containing temperature sensitive mutant *repA* (A79G) expressing RecE and RecT homologs from *Paracoccaceae* bacterium isolate SJ630, original operon; Kan^r	This study
2.4.1/pIND4-oriT-PaRecET	Wild-type 2.4.1 carrying plasmid pIPaRecET expressing RecE and RecT homologs from *Paracoccaceae* bacterium isolate SJ630, original operon; Kan^r	This study
ZX-5/pIND4-oriT-PaRecET	Wild-type ZX-5 carrying plasmid pIPaRecET expressing RecE and RecT homologs from *Paracoccaceae* bacterium isolate SJ630, original operon; Kan^r	This study
H. bluephagenesis		
TD1.0 ΔPS1234Δ50k (D50)	The RNAP module of MmP1 was integrated into the chromosome of *H. bluephagenesis* TD01. ΔPS1 cluster, ΔPS2 cluster, ΔPS4 cluster, ΔPS3 cluster, Δ50 kb of flagellar cluster	(3)
D50/pK162-AsRecET	D50 carrying plasmid pK162-AsRecET expressing RecE and RecT homologs from *A.solimangrovi* str. HB172011; Chl^r	This study
D50/pK162-PaRecET	D50 carrying plasmid pK162-PaRecET expressing RecE and RecT homologs from *Paracoccaceae* bacterium isolate SJ630; Chl^r	This study
D50/pK162-EcRecET	D50 carrying plasmid pK162-EcRecET expressing $RecE_{562}$ and RecT from *E. coli* K-12,; Chl^r	This study
D50/pK162-Redαβ	D50 carrying plasmid pK162-Redαβ expressing Redα and Redβ from *E. coli* phage Lambda; Chl^r	This study
D50/pK162-BpRecET	D50 carrying plasmid pK162-BpRecET expressing RecE and RecT homologs from *Bisbaumannia pacifica*; Chl^r	This study
D50/pK162-ChRecET	D50 carrying plasmid pK162-ChRecET expressing RecE and RecT homologs from *Chromohalobacter sp.* 296-RDG; Chl^r	This study
D50/pK162-VjRecET	D50 carrying plasmid pK162-VjRecET expressing RecE and RecT homologs from *Vreelandella janggokensis*; Chl^r	This study
D50/pK162-VvRecET	D50 carrying plasmid pK162-VvRecET expressing RecE and RecT homologs from *Vreelandella venusta*; Chl^r	This study
D50/pK162-TdRecET	D50 carrying plasmid pK162-TdRecET expressing RecE and RecT homologs from *Thioclava sp.* DLFJ5-1; Chl^r	This study
D50/pK162-HDPRecET	D50 carrying plasmid pK162-HDPRecET expressing RecE and RecT homologs from *Halomonas sp.* DP5N; Chl^r	This study
D50/pK162-HLBRecET	D50 carrying plasmid pK162-HLBRecET expressing RecE and RecT homologs from *Halomonas sp.* LBP4; Chl^r	This study
D50/pK162-HBNRecET	D50 carrying plasmid pK162-HBNRecET expressing RecE and RecT homologs from *Halomonas sp.* BN32; Chl^r	This study

Table S2. Antibiotics used in this study.

Antibiotic	Kanamycin	Apramycin	Tetracycline	Chloramphenicol	Spectinomycin
E. coli	50 μg/mL	25 μg/mL	10 μg/mL	30 μg/mL	100 μg/mL
R. sphaeroides	25 μg/mL	10 μg/mL	-	-	-
H. bluephagenesis	-	-	-	25 μg/mL	100 μg/mL

Table S3. Plasmids used in this study.

Plasmid	Features	Resource/Ref
pBBR1MCSK_new(pBBR)	Broad-host-range plasmid pBBR1MCSK2, which features a versatile origin of replication (*rep*) from plasmid pBBR1; Kan^r	This lab
pK18mobsacB(pK18)	ColE1 origin of replication for stable maintenance in *E. coli*, multiple cloning sites (MCS) for gene insertion, a kanamycin resistance gene for selection, the *sacB* gene for counter-selection and containing the *oriT* element recognized by the RP4 system; Kan^r	(4)
pIND4-oriT (pIND4T)	Derived from pIND4 containing the *oriT* element recognized by the RP4 system, *oriT* Kan^r	(5); This lab
pBBR-rfp	Insertion of the red fluorescent protein gene (rfp) between *Nco* I and *Bam*H I sites of pBBR; Kan^r	This lab
pSET152	PCR template for amplification of apramycin resistance cassette; Apr^r	(6)
pSC101-BAD-αβγA-tet	PCR template for amplification of *redαβγ* gene; Kan^r	(7)
pK18-dA	Insertion of *prrA*$_{HY01}$ gene homologous arms and *aprR* resistance gene expression cassette between the *Eco*R I and *Hind* III sites of pK18mobsacB; Apr^r Kan^r	This study
pIND4-oriT-dR	Insertion of the *aprR* resistance gene expression cassette between the *Nco* I and *Hind* III sites of pIND4-oriT; Apr^r Kan^r	This study
pK18-dwtA	Insertion of *prrA*$_{241}$ gene homologous arms and *aprR* resistance gene expression cassette between the *Eco*R I and *Hind* III sites of pK18mobsacB; Apr^r Kan^r	This study
pIND4-oriT-Redαβγ	Insertion of original operon of Lambda phage *redαβγ* between the *Nco* I and *Hind* III sites of pIND4T; Kan^r	This study
pIND4-oriT-Redαβγ-RBS	Insertion of Lambda phage *redαβγ* connected by *puhA* RBS between the *Nco* I and *Hind* III sites of pIND4T; Kan^r	This study
pIND4-oriT-EcRecET	Insertion of original operon of *E. coli recE*$_{555}$ and *recT* between the *Nco* I and *Hind* III sites of pIND4T; Kan^r	This study
pIND4-oriT-EcRecET-RBS	Insertion of *E. coli recE*$_{555}$ and *recT* connected by *puhA* RBS between the *Nco* I and *Hind* III sites of pIND4T; Kan^r	This study
pIND4-oriT-AsRecET	Insertion of original operon of *A. solimangrovi* str. HB172011 *recE* and *recT* homologs between the *Nco* I and *Hind* III sites of pIND4T; Kan^r	This study
pIND4-oriT-AsRecET-RBS	Insertion of *A. solimangrovi* str. HB172011 *recE* and *recT* homologs connected by *puhA* RBS between the *Nco* I and *Hind* III sites of pIND4T; Kan^r	This study
pIND4-oriT-AoRecET	Insertion of original operon of *A. oryzifermentans* str. dm *recE* and *recT* homologs between the *Nco* I and *Hind* III sites of pIND4T; Kan^r	This study
pIND4-oriT-AoRecET-RBS	Insertion of *A. oryzifermentans* str. dm *recE* and *recT* homologs connected by *puhA* RBS between the *Nco* I and *Hind* III sites of pIND4T; Kan^r	This study

(Continued)

Plasmid	Features	Resource/Ref
pIND4-oriT-NbRecET	Insertion of original operon of *N. basaltis* str. UMTGB225 *recE* and *recT* homologs between the *Nco* I and *Hind* III sites of pIND4T; Kan^r	This study
pIND4-oriT-PaRecET	Insertion of original operon of *Paracoccaceae* bacterium isolate SJ630 *recE* and *recT* homologs between the *Nco* I and *Hind* III sites of pIND4T; Kan^r	This study
pIND4-oriT-AtrRecT	Insertion of *A. tropicalis* str. dm *recT* expressing AtrRecT between the *Nco* I and *Hind* III sites of pIND4T; Kan^r	This study
pIND4-oriT-AokRecT	Insertion of *A. okinawensis recT* expressing AokRecT between the *Nco* I and *Hind* III sites of pIND4T; Kan^r	This study
pIND4-oriT-BanRecT	Insertion of *B. anthropic recT* expressing BanRecT between the *Nco* I and *Hind* III sites of pIND4T; Kan^r	This study
pIND4-oriT-BpaRecT	Insertion of *B. pacifica recT* expressing BpaRecT between the *Nco* I and *Hind* III sites of pIND4T; Kan^r	This study
pIND4-oriT-OveRecT	Insertion of O*. vermis recT* expressing OveRecT between the *Nco* I and *Hind* III sites of pIND4T; Kan^r	This study
pIND4-oriT-TteRecT	Insertion of *T. tepidiphila recT* expressing TteRecT between the *Nco* I and *Hind* III sites of pIND4T; Kan^r	This study
pIND4T-PaTm	The pBBR plasmid with a temperature-sensitive mutant replication protein (A73G), carrying the *lacI*, *kanR*, and original Pa*recET* operon from pIPaRecET; Kan^r	This study
pK162-AsRecET	Insertion of gene locus of AsRecET downstream promoter PW186; Chl^r	This study
pK162-PaRecET	Insertion of gene locus of PaRecET downstream promoter PW186; Chl^r	This study
pK162-BpRecET	Insertion of gene locus of BpRecET downstream promoter PW186; Chl^r	This study
pK162-ChRecET	Insertion of gene locus of ChRecET downstream promoter PW186; Chl^r	This study
pK162-VjRecET	Insertion of gene locus of VjRecET downstream promoter PW186; Chl^r	This study
pK162-VvRecET	Insertion of gene locus of VvRecET downstream promoter PW186; Chl^r	This study
pK162-TdRecET	Insertion of gene locus of TdRecET downstream promoter PW186; Chl^r	This study
pK162-HBNRecET	Insertion of gene locus of HBNRecET downstream promoter PW186; Chl^r	This study
pK162-HDPRecET	Insertion of gene locus of HDPRecET downstream promoter PW186; Chl^r	This study
pK162-HLBRecET	Insertion of gene locus of HLBRecET downstream promoter PW186; Chl^r	This study
pK162-ECRecET	Insertion of gene locus of ECRecE$_{555}$T downstream promoter PW186; Chl^r	This study
pK162-Redαβ	Insertion of gene locus of Redαβ downstream promoter PW186; Chl^r	This study

Table S4. Primers used in this study.

Name	Sequence (5' → 3')
Plasmid construction	
C1	ATTAAAGAGGAGAAATTAACCatgaacacctcaacccaaacc
C2	AGTCCAAGCTCAGCTAATTAggaccatcgctgcgttgc
C3	TTAAAGAGGAGAAATTAACCcatgaccgacctcgactttgac
C4	AGTCCAAGCTCAGCTAATTAtcacgccacctttccagac
C5	TTAAAGAGGAGAAATTAACCcatggttatttctaaatctgggatatatgacctaac
C6	AGTCCAAGCTCAGCTAATTAtcattcgccctctcccgg
C7	TTAAAGAGGAGAAATTAACCatgcgcattgaccgccc
C8	AGTCCAAGCTCAGCTAATTattacgaggcatcgcggg
C9	TTAAAGAGGAGAAATTAACCacgacgttgtaaaacgacggc
C10	AGTCCAAGCTCAGCTAATTttaggcgaacacctcggcc
C11	TTAAAGAGGAGAAATTAACCatgaatgatccggaggaaatggaag
C12	GAGTCCAAGCTCAGCTAATTAttattcctctgaattatcgattacactgtattc
C13	TTAAAGAGGAGAAATTAACCatggatattaatactgaaactgagatcaagc
C14	GTCCAAGCTCAGCTAATTactatcgccattgctccccaaatac
C15	ATTATACGAAGTTATGTGCCAGGtctgacgctcagtggaacg
C16	CAGCCAATCGACTGGCG
C17	ACAGCTATGACATGATTACGccgtcagttccgaaagggag
C18	TGGCACATAACTTCGTATAATGTATGCTATACGAACGGTacgcatctacgaaatgtgcg
C19	GCCAGTCGATTGGCTGAgtcatcgtccacgagaagcag
C20	ACGACGGCCAGTGCCAAGCTTcgtggacatcctgacgg
C21	TTAAAGAGGAGAAATTAACCtcagccaatcgactggcgag
C22	AGTCCAAGCTCAGCTAATTAtctgacgctcagtggaacg
C23	CGAATTCGCGCGGCTATACTCACTAGACTTTGCTtcgcaaagtcgtgaccgcctacggc
C24	CTTcGCGTTCCGTTTGCACCC
C25	GGTGCAAACGGAACGCgAAGCGCATGAGGCGTGG
C26	GCAAAAGGGAGAAATGGGGCcgcggggaggcagacaag
C27	GCCCCATTTCTCCCTTTTGC
C28	AGCAAAGTCTAGTGAGTATAgccgcgcgaattcgagctc
C29	ACAGCTATGACATGATTACGCcgtcagttccgaaagggag
C30	TGTATGCTATACGAACGGTAacgtggtcgaggtgctg
C32	CGTAATCATGTCATAGCTGTTTCCTGT
C806	GAGTCCAAGCTCAGCTAATtatcacccctcgtcctcgtc
C707	TTAAGGGGTCCTCCGGTGTCtcatgctgcgtctccatgag
C808	GACACCGGAGGACCCCTTAAatgaaccagaccaccgcc
C505	TTTAAGGGGTCCTCCGGTGTCtcacgcaacttccttccgc
C604	AGTCCAAGCTCAGCTAATTAtcatgctggcacttcttctttc
C606	GACACCGGAGGACCCCTTAAatgaacgcccctcagaaac
C1111	TTAAGGGGTCCTCCGGTGTCttagtcatttgcatattccttagcccag
C1212	GACACCGGAGGACCCCTTAAatgactaagcaaccaccaatcgc

(Continued)

Name	Sequence (5' → 3')
C1309	GACACCGGAGGACCCCTTAAatgacaccggacattatcctgc
C1311	TTAAGGGGTCCTCCGGTGTCtcatgctgccaccttctgct
C1313	TTAAAGAGGAGAAATTAACCatgatgagtactgcactcgcaacg
C1410	CTATCGCCATTGCTCCCCAAAT
C1412	TTGGGGAGCAATGGCGATAGGACACCGGAGGACCCCTTAAatggatattaatactgaaactgagatcaag c
C1414	GAGTCCAAGCTCAGCTAATTattatacctctgaatcaatatcaacctggtg
C1621	TTAAACAAAATTATTTGTAGAGGCTGTTTCGTCCT
C1622	ACTAGAGCTGCTAACAAAGCCCG
CSPE1	CTACAAATAATTTTGTTTAAgcatgctgatcggcacgt
CSPE2	GCTTTGTTAGCAGCTCTAGTTtatttgccgactaccttggtgatct
CBP1	AGAAAGAGGAGAAATACTAGatgaccgcgctgacgcgacaaac
CBP2	GATGCCTGGCTTATCAGGCTttatgccgcctcaagagaggccatggcgtc
CCH1	AGAAAGAGGAGAAATACTAGatgaaccaacctgcgcaagt
CCH2	GATGCCTGGCTTATCAGGCTtcatgcggctttgcgtg
CJA1	AGAAAGAGGAGAAATACTAGatgtctaccgccgtccttactt
CJA2	GATGCCTGGCTTATCAGGCTttattgctgctgggcggac
CBN1	AGAAAGAGGAGAAATACTAGatgagcaacgcagtagccac
CBN2	GATGCCTGGCTTATCAGGCTctacgctgccttgcttcgc
CLB1	AGAAAGAGGAGAAATACTAGatgagcaatgcaactgtccac
CLB2	ATGCCTGGCTTATCAGGCTgttaccagaaacaagagtccacgt
CDP1	AGAAAGAGGAGAAATACTAGatgaatgcaattacacgacccaag
CDP2	GATGCCTGGCTTATCAGGCTtcaagcggcgcgaagatg
CTD1	AGAAAGAGGAGAAATACTAGatgtctgtcgaaccatcccg
CTD2	GATGCCTGGCTTATCAGGCTatcagatttccggcgcg
CVV1	AGAAAGAGGAGAAATACTAGatgccaacactatcatttgctgac
CVV2	GATGCCTGGCTTATCAGGCTccatttcgtcgtttgtaaccgc
CEC1	AGAAAGAGGAGAAATACTAGatgccgcacaatgagaatgct
CEC2	GATGCCTGGCTTATCAGGCTttattcctctgaattatcgat
CAS1	AGAAAGAGGAGAAATACTAGatgcgcattgaccgcc
CAS2	GATGCCTGGCTTATCAGGCTtcacccctcgtcctcgtc
CRED1	AGAAAGAGGAGAAATACTAGatgagtactgcactcgcaacg
CRED2	GATGCCTGGCTTATCAGGCTctatcgccattgctccccaaat
COVE1	TTAAAGAGGAGAAATTAACCatgaaccaggtggcgac
COVE2	AGTCCAAGCTCAGCTAATTActagacctcgggcaggtcgt
CBAN1	ATTAtCATGGAAGCTTATGGtcactcgacctcgtccacct
CBAN2	TTAAAGAGGAGAAATTAACatgaatgacatcagtacgcagcag
Verification	
SV1	AGTGCCACCTGACGTCTAAG
SV2	TTGGCAAAATCCTGTATATCGTGCG
F24	AGCGGATAACAATTTCACACAGGA

(Continued)

Name	Sequence (5' → 3')
R24	CGCCAGGGTTTTCCCAGTCACGAC
Gene editing	
DR1101	CACCTCGAGAAAATTTATCAAAAAGAGTGTTG
DR1102	TTCTCACCAATAAAAAACGCCCG
DA4001	CACTTGGGCTTGAAGCGACT
DA4002	TCTCGATCGATCCCAAGCGC
DA2201	ATTGCACTTTCGGGTAGTCGAATAC
DA2202	ACGGGCTTCGTCGACTTC
DA1701	CGCCTGCCAATGAAAAAGGC
DA1702	CGAGCGAATTTCGTGCTTCG
DA1101	ATAGGTCAGCGCGGGCT
DA1102	GCAGAAGTCCTATCAAGCATCCAG
DA601	GTGCATGTTGAGCCTCCGG
DA602	ACTGAGGATCTGGTATTCGAACTCG
DA401	GCGGTCTCCGAGACGTT
DA402	ACTCGGGGCCGACAGG
DA301	GACGTTGCGGTCGCACATTT
DA302	GACAGGTCCCTGCTTCTCGT
DWA1701	CGCCTGCCAATGAAAAAGGC
DHR1501	ACACCGCTCGTCACATCCTG
DHR1502	AGCATGACCTCTAGTAATCCGGATATAGTTC
Oligo-recombineering	
dupp-2-10	CGTCTGGATCGTCGTCGTGGTCATGTCGAGCTCGCGCGTGtttataacatGGCCAGAAGCAGGCTGATCTCGCGCAGAAGGCGCCGGAAG

Table S5. Analysis of SSB-C7 compatibility in hosts with endogenous Red/ET systems (Derived from Ref. (8))

Receipt Host	Origin of RecET Homologs	SSB-C7-R	SSB-C7-D	Ref.
Gram-positive				
Mycobacterium smegmatis	Gp60 and Gp61 from Mycobacteriophage Che9c (homologs of RecETEc)	ADDEPPF	GDDEPPF	(9,10)
Mycobacterium tuberculosis	Gp60 and Gp61 from Mycobacteriophage Che9c (homologs of RecETEc)	GDDEPPF	GDDEPPF	
Lactobacillus plantarum	Lp_0640-41-42 (homolog of RecT *L.reuteri*) from prophage P1 operon from *L. plantarum* WCFS1	SDDDLPF	SDDDLPF	(11)
Lactobacillus casei	LCABL_13040–50-60 (homolog of λ-Redγβα) coupled to Redγ	ATDDLPF	ATDDLPF	(12)
Corynebacterium glutamicum	WP_010191133.1 and WP_010191134.1 from *C. aurimucosum* (homologs of RecETEc)	ADDEPPF	ADAEPPF	(13)
Bacillus licheniformis	BPR1 (homolog of RecT) from Bacillus phage 049ML001	SDDDLPF	-	(14)
Gram-negative				
Pseudomonas syringae	RecTE from *P. syringae* pv. syringae B728a	FDDDIPF	FDDDIPF	(15)
Acinetobacter baumannii	RecAB (λ Redβα homolog) from *A. baumannii* IS-123	LDDDLPF	LDDDLPF	(16)
Photorhabdus luminescens	Pluγβα from *P. luminiscens* prophage	FDDDIPF	FDDDIPF	(17)
Xenorhabdus stockiae	Pluγβα from *P. luminiscens* prophage	FDDDIPF	FDDDIPF	
Vibrio natriegens	SXT Beta and Exo found in SXT mobile element coupled to λ Gam	LDDDLPF	FDDDIPF	(18)
Burkholderiales	Redαβ7029 (λ Redβα) from *Schlegelella brevitalea* (previously known as *Burkholderiales* strain) DSM 7029	MDDDIPF	MDDDIPF	(19)
Pseudomonas aeruginosa	RecTE from *P. syringae* pv. syringae B728a	FDDDIPF	FDDDIPF	
Pseudomonas fluorescens	RecTE from *P. syringae* pv. syringae B728a	FDDDIPF	FDDDIPF	
Pseudomonas putida	BAS from *Pseudomonas* PhagevB_PaeP_Tr60_Ab31	FDDDIPF	FDDDIPL	(20)
Pseudomonas syringae	BAS from *Pseudomonas* PhagevB_PaeP_Tr60_Ab31	FDDDIPF	FDDDIPL	
Pseudomonas aeruginosa	BAS from *Pseudomonas* PhagevB_PaeP_Tr60_Ab31	FDDDIPF	FDDDIPL	
Pseudomonas protegens	RecTE from *P. syringae* pv. syringae B728a	FDDDIPF	FDDDIPF	(21)

(Continued)

Receipt Host	Origin of RecET Homologs	SSB-C7-R	SSB-C7-D	Ref.
Paraburkholderia megapolitana	Redαβ7029 (λ Redβα) from *Schlegelella brevitalea* (previously known as *Burkholderiales* strain) DSM 7029	MDDDIPF	MDDDIPF	(22)
Pseudomonas parafulva	RecTE from *P. syringae* pv. syringae B728a	FDDDIPF	FDDDIPF	(23)
Burkholderia gladioli	BAS from *P. aeruginosa* phage Ab3 (homolog of λ-Redβα) coupled to λ Gam	MDDDIPF	FDDDIPL	(24)
Burkholderia glumae	RecEThTJI49 from *Burkholderia* sp. TJI49 (homolog of RecETEc)	MDDDIPF	MDDDIPF	(25)
	RecEThe_bdu8 from *Burkholderia* sp. BDU8		MDDDIPF	
	RecETh1h2e_yi23 from *Burkholderia cordobensis* YI23		MDDDIPF	
Rhizobium rhizogenes	ETRHI483 from *Rhizobium sp.* Root483D2 (homolog of RecETEc) coupled to Pluγ	LDDDIPF	MDDDIPF	
	RecETHI145 from *Rhizobium sp*. LC145		LDDDIPF	
	RecETh1h2P3_rhi597 from *Rhizobium leguminosarum* bv. trifolii WSM597		LDDDIPF	
	RecETh1h2h3h4_agrob6 from *Agrobacterium tumefaciens* str. B6a		MDDDIPF	
Agrobacterium tumefaciens	ETRHI145 from *Rhizobium sp.* LC145	MDDDIPF	LDDDIPF	(26)
	RecETh1h2P3_rhi597 from *Rhizobium leguminosarum* bv. trifolii WSM597		LDDDIPF	
	RecETh1h2h3h4_agrob6 from *Agrobacterium tumefaciens* str. B6		MDDDIPF	
	ETRHI483 from *Rhizobium sp*. Root483D2 (homolog of RecETEc) coupled to Pluγ		MDDDIPF	
Agrobacterium fabrum	pluγETRHI145 from *Rhizobium sp.* LC145	MDDDIPF	LDDDIPF	
	RecETh1h2h3h4_agrob6 from *Agrobacterium tumefaciens* str. B6		MDDDIPF	
	RecETh1h2P3_rhi597 from *Rhizobium leguminosarum* bv. trifolii WSM597		LDDDIPF	
	ETRHI483 from *Rhizobium sp.* Root483D2 (homolog of RecETEc) coupled to Pluγ		MDDDIPF	
Xenorhabdus stockiae	XBJ1_1171, XBJ1_1172 and XBJ1_1173 from *X. bovienii* strain SS-2004	FDDDIPF	FDDDIPF	(27)

Table S6. SSB-C7 compatibility of the Lambda Red system in reported host strains (Derived from Ref. (8))

Receipt Host	SSB-C7-R	Activity	B-Score[a]	Fixed Score[b]	Ref.
Gram-positive					
Bacillus subtilis	SDDDLPF	A	31	10	(28,29)
Corynebacterium glutamicum	ADDEPPF	I	22	19	(13)
Staphylococcus aureus	SDDDLPF	I	31	10	(30)
Mycobacterium smegmatis	ADDEPPF	I	22	19	(9,10)
Mycobacterium tuberculosis	GDDEPPF	I	21	20	
Lactobacillus casei	TTDDLPF	A	24	17	(12)
Lactobacillus plantarum	SDDDLPF	I	31	10	(11)
Gram-negative					
Salmonella enterica	FDDDIPF	A	41	0	(31)
Citrobacter freundii	FDDDIPF	A	41	0	(32)
Klebsiella pneumoniae	FDDDIPF	A	41	0	(33)
Klebsiella aerogenes	FDDDIPF	A	41	0	(34)
Pantoea ananatis	FDDDIPF	A	41	0	
Shigella sonnei	FDDDIPF	A	41	0	(35)
Shigella dysenteriae	FDDDIPF	A	41	0	
Shigella flexneri	FDDDIPF	A	41	0	(36)
Yersinia pseudotuberculosis	FDDDIPF	A	41	0	(37)
Yersinia pestis	FDDDIPF	A	41	0	
Yersinia enterocolitica	FDDDIPF	A	41	0	(38)
Citrobacter rodentium	FDDDIPF	A	41	0	(39)
Serratia marcescens	FDDDIPF	A	41	0	(40)
Pseudomonas aeruginosa	FDDDIPF	A	41	0	(41)
Pseudomonas putida	FDDDIPF	A	41	0	(42)
Edwardsiella ictaluri	FDDDIPF	A	41	0	(43)
Aeromonas hydrophila	FDDDIPF	A	41	0	
Vibrio cholerae	FDDDIPF	A	41	0	(44)
Burkholderia cepacia	MDDDIPF	A	35	6	(45)
Burkholderia thailandensis	MDDDIPF	A	35	6	(46)
Burkholderia pseudomalle	MDDDIPF	A	35	6	
Pseudomonas fluorescens	FDDDIPF	I	41	0	(20,47)
Pseudomonas taiwanensis	FDDDIPF	A	41	0	(47)
Agrobacterium tumefaciens	MDDDIPF	A	35	6	(48) (26)
Zymomonas mobilis	LDDDVPF	A	34	7	(49)
Pseudomonas syringae	FDDDIPF	A	41	0	(15,20)
Pseudomonas parafulva	FDDDIPF	A	41	0	(23)
Photorhabdus luminescens	FDDDIPF	A	41	0	(17)
Xenorhabdus stockiae	FDDDIPF	I	41	0	
Vibrio natriegens	LDDDLPF	A	33	8	(18)

(Continued)

Receipt Host	SSB-C7-R	Activity	B-Score[a]	Fixed Score[b]	Ref.
Acinetobacter baumannii	LDDDLPF	I	33	8	(16)
Paraburkholderia megapolitana	MDDDIPF	A	35	6	(22)
Burkholderia gladioli	MDDDIPF	A	35	6	(24)
Burkholderia glumae	MDDDIPF	I	35	6	(25)
Rhizobium rhizogenes	LDDDIPF	I	35	6	(26)
Agrobacterium fabrum	MDDDIPF	A	35	6	
Caldimonas brevitalea	MDDDIPF	I	35	6	(19)
Shewanella oneidensis	WDDDIPF	I	36	5	(50)

a. B-score is obtained using the BLOSUM62 matrix by substituting the SSB-C7 of *E. coli* SSB with FDDDIPF. b. The corrected score is the identical substitution score minus the non-identical substitution score.

Table S7. Detail information of RecT candiates.

Homologs	Gebank ID	Description	Identity (to EcRecT/Redβ)
AsRecT	TPE53052.1	Hypothetical protein	19.46%/18.01%
AoRecT	ASL39289.1	Hypothetical protein	32.39%/18.57%
NbRecT	KFB10375.1	Recombinase RecT	27.27%/16.52%
PaRecT	MCC6863621.1	Recombinase RecT	28.76%/15.28%
SpRecT	MBM2318339.1	Recombinase RecT	12.74%/14.63%
AtrRecT	WP_061487463.1	phage recombination protein Bet	-
AokRecT	WP_086638090.1	Recombinase RecT	-
BpaRecT	WP_076978705.1	phage recombination protein Bet	-
TteRecT	WP_064782245.1	Recombinase RecT	-
BanRecT	WP_043062338.1	recombinase family protein	-
OveRecT	WP_105541778.1	Recombinase RecT	-
ChRecT	WP_280568162.1	Recombinase RecT	See Fig. 5h
BpRecT	WP_146804501.1	Recombinase RecT	See Fig. 5h
VvRecT	WP_317997658.1	RecT family recombinase	See Fig. 5h
VjRecT	WP_268901324.1	hypothetical protein	See Fig. 5h
TdRecT	WP_210402715.1	recombinase RecT	See Fig. 5h
HDPRecT	WP_222569937.1	recombinase RecT	-
HBNRecT	WP_045993464.1	recombinase RecT	-
HLBRecT	WP_110284689.1	phage recombination protein Bet	-

Table S8. Accession-level comparison of SSB-C7- and SSB-C8-based search results.

Metric	Records/percentage
Ct7-based hits	5,795
Ct8-based hits	4,352
Shared Ct7/Ct8 hits	4,342
Ct7-only hits	1,453
Ct8-only hits	10
Ct8 hits overlapping with Ct7	99.77%
Ct7 hits retained by Ct8	74.93%

Supplementary methods

Code implementation for *Rhodobacter sphaeroides*

Genome Assembly Information Retrieval

To retrieve genome assembly information in step 1 (Fig. 1c), we searched for "Alphaproteobacteria" on the NCBI Assembly page. The source database was set to "RefSeq" and the file type to "FASTA (.faa)". The downloaded files were decompressed to obtain protein sequence files from all assemblies. As of July 23, 2023, a total of 18,841 genome assemblies were available.

BLAST Database Construction

For the construction of BLAST databases for the 18,841 protein assemblies in step 1 (Fig. 1c), a separate database was created for each 'faa' record. This was done to directly search for SSB protein sequences within each assembly (if present). A Python script was developed for this purpose. The script iterates through each '.faa' file in the directory, executing the `makeblastdb` command from the NCBI BLAST+ suite with parameters set to `-dbtype prot` and `-parse_seqids` to facilitate subsequent retrieval of sequences by ID. The constructed databases were named according to their respective assembly accession numbers and stored in a designated directory.

Subsequently, in step 4 (Fig. 1c), a merged database was constructed with the same parameters, except that a simple script was used to combine protein sequences from the filtered 'faa' files into a single FASTA file. This database, named SSBC7db, includes all protein sequences from databases containing the same SSB-C7.

Module A: Screening Assemblies for Specified SSB-C7

To search for records containing specific SSB sequence motifs in step 2 (Fig. 1c), we employed Pattern Hit Initiated BLAST (PHI-BLAST) (v2.16.0+) (51). A script was developed to automate this process, iterating through all specified databases and executing PHI-BLAST. The query sequence was the SSB protein from *R. sphaeroides* (*rsp_1793*, SSB_{Rsp}), and the parameters were set as follows: `-max_target_seqs 1`, `-evalue 0.005`, and output format `–outputfmt "6 sseqid sseq"`. The pattern file used for the search contained the following:

ID 1

PA X-LDDEIPF

Extract sequence IDs from the results, retrieve the corresponding assemblies, and then merge these databases according to the method described earlier to obtain the final SSBC7db.

Module B: Searching for SSAPs and Exos Using PSI-BLAST

We conducted PSI-BLAST searches to identify SSAPs and Exos using Position-Specific Scoring Matrices (PSSMs). Four PSSMs were constructed for RecT, Redβ, RecE, and Redα using PSI-BLAST. Sequences for these protein families were downloaded from the Conserved Domain Database (CDD) (52) at NCBI and used to build four corresponding protein databases with the `makeblastdb` command. The representative sequences for each family (EcRecT, Redβ, EcRecE555, and Redα) were used as queries in the `psiblast` program, with parameters set to `-num_iterations 3 -evalue 0.005`, and the specific database (`-db`).

The PSSM files generated were used for subsequent PSI-BLAST searches, with parameters detailed as below.

Table. Unique parameters for four queries of PSSMs

Input PSSM	E-value	Database	Iteration numbers	Notes
Pfam03837	0.05	Pfam03837	5	RecT
TIGR01913	0.05	TIGR01913	9	Redβ
PRK09729	0.0005	PRK09729	2	RecE
Pfam09588	0.05	Pfam09588	6	Redα

Four PSI-BLAST runs were performed consecutively, each using one of the four PSSMs as the input (`-in_pssm`), with SSBC7db as the database (`-db`) and the output format set to `-outputfmt "6 sseqid sseq"`. The E-value (`-evalue`) and the number of iterations (`-num_iterations`) were configured according to the table above. Sequence IDs were then extracted from the results that are then merged homologously. RecT and Redβ IDs were combined to form the SSAPs dataset, and RecE and Redα IDs were combined to form the Exos dataset. These IDs were crucial for subsequent gene upstream and downstream searches.

Identifying Paired RecET Candidates

To identify paired RecET proteins, a custom Python script was developed to process the RecE and RecT candidate lists obtained in step 6. The script compares sequence identifiers (IDs) from the SSAP (RecT) and exonuclease (RecE) datasets, assuming that gene identifiers within each genome assembly are sequentially ordered according to their genomic loci. For each ID, the alphanumeric prefix and numeric suffix were extracted, and all entries were combined and sorted by prefix and numeric order. Adjacent IDs were then compared: if two IDs differed by no more than 2 in their numeric part and originated from different datasets (RecT vs. RecE), they were considered a potential RecET pair. All qualifying ID pairs were written to an output file and classified by their respective source.

To retrieve complete protein sequences for the identified RecET candidates, another script employed the `blastdbcmd` utility from the NCBI BLAST+ suite to extract full-length sequences directly from the BLAST databases using their sequence IDs. The retrieved sequences were exported to designated directories for subsequent alignment and phylogenetic analysis. command from the BLAST+ toolkit. This script retrieves the full protein or nucleic acid sequences based on the sequence IDs and outputs them to a specified location.

Module C: Sequence Filtering and Deduplication

Deduplication of complete RecT sequences in step 7 was performed using CD-HIT(53) with a similarity threshold of 0.95. The deduplicated and paired RecT sequences were then used to search for 16S rDNA sequences within the corresponding genome assembly files. Manual inspection was conducted to remove incomplete sequences (containing ambiguous bases 'N') and incorrectly extracted 16S rDNA sequences. The validated 16S sequences were matched to their corresponding RecT sequences to facilitate subsequent phylogenetic analysis and selection of high-confidence RecET candidates.

Code Implementation for *Halomonas bluephagenesis*

To evaluate portability in a distinct physiological context, we reapplied the SSB-C7–guided ETfinder workflow to *Halomonas* using a lightweight, database-free variant that queries **NR/RefSeq** directly instead of building per-

assembly BLAST databases, and we omitted nuclease–SSAP pairing in this run.

Seed & motif-constrained retrieval. The unique *Halomonas sp.* TD01 SSB (C7 = FDDEIPF) was used as the seed. We performed PSI-BLAST with a PHI pattern constraint for the C7 motif (FDDEIPF) against NR database, retaining subject IDs (accessions) per hit. Protein placements were normalized to RefSeq assemblies by resolving each hit through the NCBI IPG/Assembly metadata (E-utilities) (https://www.ncbi.nlm.nih.gov/books/NBK25501/), filtering to GCF accessions and deduplicating at the assembly level.

Assembly normalization & subsetting. The resulting GCF list was merged with the RefSeq assembly summary to obtain TaxIDs/organisms, then used to create an NR alias subset (blastdb_aliastool) restricted to assemblies encoding FDDEIPF-SSBs. This subset served as the search space for SSAP discovery.

RecT homologos discovery. Within the subset, we ran iterative PSI-BLAST using a Pfam-based SSAP family PSSM (e.g., PF03837 & TIGR01913) to capture divergent SSAPs. Subject lists (e.g., WP_ accessions) from both searches were unioned and de-redundantized to form the nonredundant SSAP set. Full-length sequences were retrieved with blastdbcmd; genomic placements were collected via efetch/ipg for optional neighborhood/context annotation consistent with the main ETfinder pipeline (±2 ORFs; hmmscan vs Pfam-A (54), $E < 1\times10^{-5}$, keep top two distinct domains per protein).

Differences from the canonical ETfinder run. (i) No per-assembly BLAST databases were built; instead, NR/RefSeq was subset on-the-fly via IPG/assembly mapping and alias databases. (ii) Nuclease–SSAP pairing was manually curated in this run (putative RecE/Redα partners were selected by neighborhood inspection rather than inferred automatically). (iii) The entire *Halomonas* run relied exclusively on command-line utilities (NCBI BLAST+ v2.16.0, EDirect, HMMER v3.4, GNU coreutils/AWK), with version-pinned invocations recorded in **Supplementary Dataset S2**. This headless, script-only design enables fully reproducible, containerizable execution without building per-assembly local databases.

Construction of the reference SSB context map for host-type classification

All bacterial reference genomes were retrieved from the NCBI Datasets repository (v16.39.0) (55) and screened for single-stranded DNA-binding protein (SSB) genes. For each genome, the ±2 neighboring genes of every SSB locus were extracted to define local genomic contexts. Protein sequences from these neighboring genes were scanned against the Pfam-A HMM library using HMMER (hmmscan, v3.4) with an E-value cutoff of 1×10^{-5}. For each protein, the two most significant non-redundant Pfam domains were retained as functional annotations. Contextual records were then cleaned by removing placeholder tags (“NO_PFAM”, “EMPTY_POS”) to generate a structured set of context elements for each SSB.

To identify representative functional signatures across taxonomic families, Pfam domain frequencies were calculated within each bacterial family based on all SSB contexts. Pfams that appeared in at least five contexts and represented ⩾ 20 % of the total contexts in that family were subjected to enrichment testing. Family-specific enrichments were assessed using a one-tailed Fisher’s exact test (implemented in SciPy v1.0 (56)) and corrected for multiple testing using the Benjamini–Hochberg false discovery rate procedure (statsmodels (57); $Q < 0.05$). The resulting significantly enriched Pfams were defined as family-level contextual signatures.

Taxonomic families sharing ⩾ 80 % overlap in their Pfam signatures (Jaccard similarity ⩾ 0.80) were merged into contextual clusters, each typically corresponding to a single bacterial order. If multiple orders co-occurred

within a cluster, an MD5-derived identifier was assigned (e.g., "Cluster-7acdf1"). Families with limited sampling (below the third-quartile threshold of total contexts) were treated as low-representation families and assigned to the most similar cluster when their Jaccard similarity to an existing cluster's signature set exceeded 0.30.

For each cluster, the total number of genomes represented (N_{sp}) and the proportion of genomes containing a single-copy SSB were calculated. Clusters with ≥ 80 % single-copy representation were designated **STRONG**, those with 50–80 % as **MOD**, and those below 50 % as **WEAK**. The final output, cluster_summary.txt, lists the cluster name, number of supporting genomes, single-copy ratio, evidence tag, and the top five most frequent Pfam domains. This summary file serves as the reference table for subsequent SSB-context scoring and classification of host-type, non-host, and suspect SSBs in the SSAP–SSB mapping workflow.

Scoring of SSB contexts and host-type classification

Domain category definitions. To define a reproducible criterion for distinguishing chromosomal (host-type) SSBs from phage- or mobile-element–associated copies, we established a set of prior rules derived from conserved genomic architectures in well-annotated reference genomes. In bacterial lineages where only a single SSB locus is present—such as *E. coli* K-12 (C7 = FDDDIPF), *Halomonas spp.* (FDDEIPF), and *R. sphaeroides* (LDDEIPF)—the host SSB consistently appears within a stable neighborhood containing UvrA-associated domains (PF17760.4_UvrA_inter; PF17755.4_UvrA_DNA-bind), translation-related markers (PF01250.20_Ribosomal_S6; PF01084.23_Ribosomal_S18), and metabolic or transport Pfams such as EAL domains, PF00083.27_Sugar_tr / PF07690.19_MFS_1, and PF00563.23_EAL. As supported by our order-level contextual survey, these domain combinations were treated as characteristic host-associated features and incorporated into the prior classification rules. By contrast, loci flanked by DDE transposases (PF01610.20_DDE_Tnp), phage integrases (PF00589.25_Phage_integrase), DUF-rich proteins, SSAP/ERF/RAD52-superfamily factors, or Gp-type recombination modules were treated as phage-like or mobile-element–associated SSBs. These contextual domain patterns, together with the presence or absence of single-copy SSB configurations, were formalized as prior classification rules and incorporated into the automated scoring system described below.

Scoring procedure. Each bacterial order was assigned a reference Pfam profile derived from the contextual clusters described above (cluster_summary.txt). For every genome, the order name was parsed from the lineage annotation, and all SSB loci within that genome were evaluated against the corresponding order-level reference profile. Each contextual Pfam element within ±2 genes of an SSB was compared with the top ≤3 Pfams in the reference signature, weighted by position (weights = 5, 4, 3, 1 for ranks 1–3 and others). Two ribosomal Pfams (PF01250.20_Ribosomal_S6 and PF01084.23_Ribosomal_S18) were assigned a fixed weight of 5 to emphasize translation-associated environments frequently co-occurring with host SSBs. For each SSB, the total context score was calculated as the sum of the maximal weighted overlaps across all context combinations. Within each genome, SSBs were ranked by their scores, and an SSB was classified as host-type when its score exceeded the next highest by at least three points ($\Delta \geq 3$) and reached an absolute score ≥ 9 (strong threshold) or ≥ 5 (weak threshold). Lower-scoring loci were classified as non-host, and ambiguous cases were labeled suspect. Orders without a defined reference signature were treated as weak orders, and all SSBs within them were marked suspect. This scoring procedure integrated contextual Pfam enrichment patterns with taxonomic information to generate the final host-anchored SSB reference table used for SSAP–SSB mapping.

Additional manual curation and rule-based classification. After automated scoring, all SSB loci labeled as suspect were further inspected manually. Contexts whose neighboring genes all contained Pfam-annotated

domains (ending with “PF”) and lacked phage- or mobile element–related domains were classified as **host**. Loci whose ±2 neighboring genes were exclusively associated with phage or mobile element proteins, or whose four contextual positions were all labeled “NO_PFAM”, were directly classified as **non-host**. When two SSB loci shared identical ±2 Pfam neighborhoods and identical SSB-Ct motifs, they were marked as **host_dup**. Loci whose neighborhoods contained domains from the RecT, ERF, or RAD52 superfamilies were annotated as **ssap_cog**. Mixed or ambiguous cases—such as those combining frequent Pfam signatures with mobile-element–associated domains, those ending with non-PF annotations, those lacking reliable genus- or family-level references, or those representing truncated SSB open reading frames—were classified as **host_low**.

Examples of each rule are provided below.

Mixed: Bacteroides distasonis SSB-C7 (DADDLPF) —NO_PFAM; PF01595.23_CNNM/PF03471.20_CorC_HlyC; PF00589.25_Phage_integrase; NO_PFAM. This context contains both housekeeping-associated Pfams (CNNM/CorC_HlyC) and a phage-related integrase domain, representing a hybrid genomic neighborhood. Accordingly, it was classified as **host_low**.

Low-reference: *Iocasia fonsfrigidae* (Class *Clostridia*) SSB-C7 (DNFDVPF) — PF09991.12_DUF2232; PF01084.23_Ribosomal_S18; PF01250.20_Ribosomal_S6; NO_PFAM. Although the neighboring genes included ribosomal domains typically associated with host-type contexts, this lineage lacked any reliable genus- or family-level reference clusters for comparison. Therefore, the locus was conservatively classified as **host_low**.

Truncated SSB: *Segatella copri* SSB-C7 (RPATREQ) — PF01648.23_ACPS; PF01595.23_CNNM/PF03471.20_CorC_HlyC; NO_PFAM; PF00278.25_Orn_DAP_Arg_deC. Although several related species within the same family possess full-length SSB-C7 motifs (e.g., DDEILPF, DDGGLPF, DDTALPF, DKNALPF, DNSALPF, EDDALPF), this locus represents a truncated SSB open reading frame. Moreover, another SSB context in the same genome lacks host-associated features (PF06908.14_YpsA; NO_PFAM; NO_PFAM; NO_PFAM). Accordingly, it was classified as **host_low**.

Construction of the SSAP–SSB mapping database for the web server

We assembled a JSON database that maps RecT (SSAP) candidates to their cognate genome assemblies and corresponding SSB loci. RecT and SSB sequences were first queried against a local NR snapshot (downloaded on 2025-02-23) using PSI-BLAST (BLAST+ v2.16.0+) with custom PSSMs for RecT and SSB, respectively. For each hit, the protein identifier was resolved to its AssemblyAccession (GCF via NCBI IPG/EDirect (efetch/esummary/xtract), and organism and strain metadata were recorded. For every accession, NCBI Datasets (v16.39.0) was used to download the corresponding .gbff, from which we parsed the ±2 ORFs around each RecT and SSB: protein accessions, product descriptions, amino-acid sequences, coordinates, and strand. Neighborhood proteins were optionally annotated against Pfam-A with hmmscan (HMMER v3.4; $E < 1\times10^{-5}$), retaining at most two distinct domains per protein.

Host SSB assignment for each assembly was performed automatically using the SSB-Ct probe and the order-level scoring table described above; records that failed automatic labeling were manually curated with the same rule set used for scoring (host/non_host/host_low/host_dup/ssap_cog). The resulting JSON serves as the canonical, versioned data source for the web server.

To ensure that experimentally tested RecT candidates span the full breadth of the RecT tree rather than clustering within a single dense lineage, ETfinder applies a two-stage stratified sampling strategy.

Stratified sampling of RecT sequences

The goal of stratification is to prevent phylogenetically clustered RecT lineages from dominating the top of the ranked list.

Selecting seed leaves to define phylogenetic regions. Users specify the desired number of RecT representatives (K). To divide the RecT phylogeny into K broad regions, ETfinder identifies K "seed" leaves that are as far apart from one another as possible along branch lengths. The procedure starts from an arbitrary leaf, then repeatedly chooses the leaf most distant from the already selected seeds. This simple farthest-point heuristic spreads the seeds across the tree and provides a natural partition of the phylogeny into K regions. Each seed defines a clade-like group containing all leaves closest to that seed.

Ranking leaves within each region. Within every region, ETfinder orders the RecT sequences by their evolutionary distance to the region's seed. This creates a structured list in which sequences near the seed (the center of that phylogenetic region) appear first, and progressively more divergent members follow.

When a target host species is provided, these distances can also incorporate host phylogenetic proximity so that RecT variants originating from hosts closer to the user-specified species rise toward the top of the ranking.

Combining regions via round-robin interleaving. After each region is individually ranked, ETfinder merges the lists using a round-robin procedure: one item is taken from region 1, then region 2, continuing across all K regions before cycling back again. This interleaving ensures that the highest positions in the final ranked list correspond to RecT variants sampled from widely separated portions of the phylogeny. As a result, no single lineage dominates the early part of the output. If a region runs out of entries, the scheduler simply continues with the remaining regions.

Interpretation. The final ranking therefore presents users with a highly diverse, phylogenetically balanced set of RecT candidates. The approach avoids redundancy, allows tunable exploration of RecT space through the single parameter K, and yields a sorted list in which early entries are both evolutionarily distant from one another and close to their respective phylogenetic centers.

Phylogenetic Tree Construction

Phylogenetic trees were constructed manually using complete selected sequences. For the RecT-associated 16S rDNA sequences, we included sequences from *E. coli* str. K-12 and *R. sphaeroides* str. 2.4.1 for comparison. For the RecT protein phylogenetic tree, we included EcRecT and Redβ sequences. Multiple sequence alignments were computed with ClustalW (58) for the *R. sphaeroides* case study and with MAFFT (v7.526; --auto) (59) in the web-server implementation. The aligned sequences were converted to the 'phy' format using Mesquite (60) (version 3.81 http://www.mesquiteproject.org). Phylogenetic trees were constructed using iqtree2 (v2.3.6) (61), with ultrafast bootstrap testing set to 1000 iterations (`-B 1000`) (62), automatic model selection enabled (`-T`) (63), and Bootstrap Nearest Neighbor Interchanges (BNNI) (`-bnni`) activated to mitigate bias from local optima. The resulting trees were visualized and refined using iTOL (64).

Host–RecT distance–identity analysis

RecT/Bet-like amino acid sequences were aligned with MAFFT (59). Pairwise amino acid identity was calculated as the number of identical amino acid residues divided by the number of alignment columns in which at least one sequence contained an amino acid. Thus, unilateral gaps were counted as mismatches, whereas columns containing gaps in both sequences were excluded.

For the *R. sphaeroides* case dataset, host phylogenetic distance was calculated as the patristic distance between host tips in the 16S rRNA tree using the `cophenetic.phylo` function in the R package ape. Patristic distance was defined as the sum of branch lengths along the path connecting two host tips. For each host, the nearest-host distance was defined as the minimum patristic distance to any other matched non-reference host. The mean nearest-host distance was used as a descriptive threshold for close-host comparisons. The main analysis included 91 non-reference host-RecT entries with both a matched 16S rRNA tree position and a RecT protein sequence. EcRecT and Lambda Red Beta were overlaid as *E. coli*-associated reference proteins using the *E. coli* 16S rRNA position. These reference points were included only for visual orientation and were excluded from the 91-entry sample statistics, threshold definition, highlighted counts, and Spearman correlation calculations.

For the *Halomonas* dataset, host topological distance was calculated as the tip-to-tip path length on the supplied *Halomonas* host tree. Because this tree used unit branch lengths rather than calibrated molecular evolutionary distances, this metric represents tree-topological distance, not substitutions per site or divergence time. For protein pairs assigned to the same host group, host topological distance was set to 0; these points represent within-host RecT/Bet-like protein-pair comparisons rather than evolutionary distance between host tips. Topologically proximate comparisons were defined as pairs with a topological distance $\leqslant 2$, where 2 was the minimum nonzero distance observed among matched host tips.

For the *Halomonas* nearest-host subset, the topologically nearest host group was identified for each focal host group. When either member of a nearest-host group pair contained multiple RecT/Bet-like homologs, all pairwise protein combinations between the two host groups were retained rather than selecting a representative sequence. Identical general host names were collapsed into a single host group, while all associated protein sequences were retained. Unclassified or genus-level host names were distributed among compatible species- or strain-level candidate tips when available. The background analysis included 73 matched host-protein entries from 53 host groups. Five high-rank MULTISPECIES entries were excluded because they could not be assigned to a specific candidate host tip: three *Gammaproteobacteria* entries, one *Pseudomonadota* entry, and one *Halomonadaceae* entry. EcRecT and Lambda Redβ were included as reference overlays only and were excluded from background counts, cutoff definition, highlighted counts, and Spearman correlation calculations.

Associations between host distance and RecT homolog amino acid identity were summarized using Spearman's rank correlation. Because pairwise comparisons are non-independent in both datasets, Spearman's rho was interpreted as a descriptive effect size rather than a formal test statistic. For the *Halomonas* dataset, this interpretation was further qualified using a unit-branch host tree, such that host distance represents tree-topological distance rather than calibrated molecular evolutionary distance. The scripts used to generate these distance–identity plots are provided as **Supplementary Code S5**.

Supplementary Dataset S1. Script-level implementation of the ETfinder strategy used in the *R. sphaeroides* case study.

Supplementary Dataset S2. Command-line workflows and output files generated using the ETfinder strategy in the

H. bluephagenesis D50 case study.

Supplementary Dataset S3. Order-level SSB reference contexts and Pfam frequency summaries used to construct the SSB-context map for ETfinder.

Supplementary Dataset S4. SSB-context scoring table for all reference genomes, including gene-neighborhood annotations, matched Pfams, and host/non-host assignments.

Supplementary Code S5. Scripts for host–RecT distance–identity analysis and visualization.

Reference

1. Pettersen, E.F., Goddard, T.D., Huang, C.C., Meng, E.C., Couch, G.S., Croll, T.I., Morris, J.H. and Ferrin, T.E. (2021) UCSF ChimeraX: Structure visualization for researchers, educators, and developers. *Protein. Sci.*, **30**, 70-82.

2. Waterhouse, A.M., Procter, J.B., Martin, D.M., Clamp, M. and Barton, G.J. (2009) Jalview Version 2--a multiple sequence alignment editor and analysis workbench. *Bioinformatics*, **25**, 1189-1191.

3. Xu, T., Chen, J., Mitra, R., Lin, L., Xie, Z., Chen, G.Q., Xiang, H. and Han, J. (2022) Deficiency of exopolysaccharides and O-antigen makes *Halomonas bluephagenesis* self-flocculating and amenable to electrotransformation. *Commun. Biol.*, **5**, 623.

4. Schäfer, A., Tauch, A., Jäger, W., Kalinowski, J., Thierbach, G. and Pühler, A. (1994) Small mobilizable multi-purpose cloning vectors derived from the Escherichia coli plasmids pK18 and pK19: selection of defined deletions in the chromosome of *Corynebacterium glutamicum*. *Gene*, **145**, 69-73.

5. Ind, A.C., Porter, S.L., Brown, M.T., Byles, E.D., de Beyer, J.A., Godfrey, S.A. and Armitage, J.P. (2009) Inducible-expression plasmid for *Rhodobacter sphaeroides* and *Paracoccus denitrificans*. *Appl. Environ. Microbiol.*, **75**, 6613-6615.

6. Bierman, M., Logan, R., O'Brien, K., Seno, E.T., Rao, R.N. and Schoner, B.E. (1992) Plasmid cloning vectors for the conjugal transfer of DNA from *Escherichia coli* to *Streptomyces* spp. *Gene*, **116**, 43-49.

7. Zhang, Y., Muyrers, J.P., Testa, G. and Stewart, A.F. (2000) DNA cloning by homologous recombination in *Escherichia coli*. *Nat. Biotechnol.*, **18**, 1314-1317.

8. Corts, A., Thomason, L.C., Costantino, N. and Court, D.L. (2022) Recombineering in non-model bacteria. *Curr. Protoc.*, **2**, e605.

9. van Kessel, J.C. and Hatfull, G.F. (2007) Recombineering in *Mycobacterium tuberculosis*. *Nat. Methods*, **4**, 147-152.

10. van Kessel, J.C. and Hatfull, G.F. (2008) Efficient point mutagenesis in mycobacteria using single-stranded DNA recombineering: characterization of antimycobacterial drug targets. *Mol. Microbiol.*, **67**, 1094-1107.

11. Yang, P., Wang, J. and Qi, Q. (2015) Prophage recombinases-mediated genome engineering in *Lactobacillus plantarum*. *Microb. Cell Fact.*, **14**, 154.

12. Xin, Y., Guo, T., Mu, Y. and Kong, J. (2017) Identification and functional analysis of potential prophage-derived recombinases for genome editing in *Lactobacillus casei*. *FEMS Microbiol. Lett.*, **364**.

13. Chang, Y., Wang, Q., Su, T. and Qi, Q. (2021) Identification of phage recombinase function unit in genus *Corynebacterium*. *Appl. Microbiol. Biotechnol.*, **105**, 5067-5075.

14. Xue, F., Ma, X., Luo, C., Li, D., Shi, G. and Li, Y. (2023) Construction of a bacteriophage-derived recombinase system in *Bacillus licheniformis* for gene deletion. *AMB Express*, **13**, 89.

15. Swingle, B., Bao, Z., Markel, E., Chambers, A. and Cartinhour, S. (2010) Recombineering using RecTE from *Pseudomonas syringae*. *Appl. Environ. Microbiol.*, **76**, 4960-4968.

16. Tucker, A.T., Nowicki, E.M., Boll, J.M., Knauf, G.A., Burdis, N.C., Trent, M.S. and Davies, B.W. (2014) Defining gene-phenotype relationships in *Acinetobacter baumannii* through one-step chromosomal gene inactivation. *mBio*, **5**, e01313-01314.

17. Yin, J., Zhu, H., Xia, L., Ding, X., Hoffmann, T., Hoffmann, M., Bian, X., Muller, R., Fu, J., Stewart, A.F. *et al.* (2015) A new recombineering system for *Photorhabdus* and *Xenorhabdus*. *Nucleic Acids Res.*, **43**, e36.

18. Lee, H.H., Ostrov, N., Gold, M.A. and Church, G.M. (2017) Recombineering in *Vibrio natriegens*. *bioRxiv*.

19. Wang, X., Zhou, H., Chen, H., Jing, X., Zheng, W., Li, R., Sun, T., Liu, J., Fu, J., Huo, L. *et al.* (2018) Discovery of recombinases enables genome mining of cryptic biosynthetic gene clusters in *Burkholderiales* species. *Proc. Natl. Acad. Sci.*, **115**, E4255-E4263.

20. Yin, J., Zheng, W., Gao, Y., Jiang, C., Shi, H., Diao, X., Li, S., Chen, H., Wang, H., Li, R. *et al.* (2019) Single-stranded DNA-binding protein and exogenous RecBCD inhibitors enhance phage-derived homologous recombination in *Pseudomonas*. *iScience*, **14**, 1-14.

21. Yu, F., Jing, X., Li, X., Wang, H., Chen, H., Zhong, L., Yin, J., Pan, D., Yin, Y., Fu, J. *et al.* (2019) Recombineering *Pseudomonas protegens* CHA0: An innovative approach that improves nitrogen fixation with impressive bactericidal potency. *Microbiol. Res.*, **218**, 58-65.

22. Zheng, W., Wang, X., Zhou, H., Zhang, Y., Li, A. and Bian, X. (2020) Establishment of recombineering genome editing system in *Paraburkholderia megapolitana* empowers activation of silent biosynthetic gene clusters. *Microb. Biotechnol.*, **13**, 397-405.

23. Zheng, W., Wang, X., Chen, Y., Dong, Y., Zhou, D., Liu, R., Zhou, H., Bian, X., Wang, H., Tu, Q. *et al.* (2021) Recombineering facilitates the discovery of natural product biosynthetic pathways in *Pseudomonas parafulva*. *Biotechnol. J.*, **16**, e2000575.

24. Chen, H., Sun, T., Bai, X., Yang, J., Yan, F., Yu, L., Tu, Q., Li, A., Tang, Y., Zhang, Y. *et al.* (2021) Genomics-driven

activation of silent biosynthetic gene clusters in *Burkholderia gladioli* by screening recombineering system. *Molecules*, **26**.

25.Li, R., Shi, H., Zhao, X., Liu, X., Duan, Q., Song, C., Chen, H., Zheng, W., Shen, Q., Wang, M. *et al.* (2021) Development and application of an efficient recombineering system for *Burkholderia glumae* and *Burkholderia plantarii*. *Microb. Biotechnol.*, **14**, 1809-1826.

26.Bian, Z., Li, S., Yang, R., Yin, J., Zhang, Y., Tu, Q., Fu, J. and Li, R. (2022) Development of a new recombineering system for *Agrobacterium* species. *Appl. Environ. Microbiol.*, **88**, e0249921.

27.Huang, X., Sun, Y., Liu, S., Li, Y., Li, C., Sun, Y., Ding, X., Xia, L., Hu, Y. and Hu, S. (2022) Recombineering using RecET-like recombinases from *Xenorhabdus* and its application in mining of natural products. *Appl. Microbiol. Biotechnol.*, **106**, 7857-7866.

28.Wang, Y., Weng, J., Waseem, R., Yin, X., Zhang, R. and Shen, Q. (2012) *Bacillus subtilis* genome editing using ssDNA with short homology regions. *Nucleic Acids Res.*, **40**, e91.

29.Sun, Z., Deng, A., Hu, T., Wu, J., Sun, Q., Bai, H., Zhang, G. and Wen, T. (2015) A high-efficiency recombineering system with PCR-based ssDNA in *Bacillus subtilis* mediated by the native phage recombinase GP35. *Appl. Microbiol. Biotechnol.*, **99**, 5151-5162.

30.Penewit, K., Holmes, E.A., McLean, K., Ren, M., Waalkes, A. and Salipante, S.J. (2018) Efficient and scalable precision genome editing in *Staphylococcus aureus* through conditional recombineering and CRISPR/Cas9-mediated counterselection. *mBio*, **9**.

31.Datta, S., Costantino, N., Zhou, X. and Court, D.L. (2008) Identification and analysis of recombineering functions from Gram-negative and Gram-positive bacteria and their phages. *Proc. Natl. Acad. Sci.*, **105**, 1626-1631.

32.Nyerges, A., Csorgo, B., Nagy, I., Balint, B., Bihari, P., Lazar, V., Apjok, G., Umenhoffer, K., Bogos, B., Posfai, G. *et al.* (2016) A highly precise and portable genome engineering method allows comparison of mutational effects across bacterial species. *Proc. Natl. Acad. Sci.*, **113**, 2502-2507.

33.Wei, D., Wang, M., Shi, J. and Hao, J. (2012) Red recombinase assisted gene replacement in *Klebsiella pneumoniae*. *J. Ind. Microbiol. Biotechnol.*, **39**, 1219-1226.

34.Wu, Y., Hao, Y., Wei, X., Shen, Q., Ding, X., Wang, L., Zhao, H. and Lu, Y. (2017) Impairment of NADH dehydrogenase and regulation of anaerobic metabolism by the small RNA RyhB and NadE for improved biohydrogen production in *Enterobacter aerogenes*. *Biotechnol. Biofuels*, **10**, 248.

35.Katashkina, J.I., Hara, Y., Golubeva, L.I., Andreeva, I.G., Kuvaeva, T.M. and Mashko, S.V. (2009) Use of the lambda Red-recombineering method for genetic engineering of *Pantoea ananatis*. *BMC Mol. Biol.*, **10**, 34.

36.Ohya, K., Handa, Y., Ogawa, M., Suzuki, M. and Sasakawa, C. (2005) IpgB1 is a novel *Shigella* effector protein involved in bacterial invasion of host cells. Its activity to promote membrane ruffling via Rac1 and Cdc42 activation. *J. Biol. Chem.*, **280**, 24022-24034.

37. Derbise, A., Lesic, B., Dacheux, D., Ghigo, J.M. and Carniel, E. (2003) A rapid and simple method for inactivating chromosomal genes in *Yersinia*. *FEMS Immunol. Med. Microbiol.*, **38**, 113-116.

38. Trülzsch, K., Sporleder, T., Igwe, E.I., Rüssmann, H. and Heesemann, J. (2004) Contribution of the major secreted yops of *Yersinia enterocolitica* O:8 to pathogenicity in the mouse infection model. *Infect. Immun.*, **72**, 5227-5234.

39. Deng, W., Puente, J.L., Gruenheid, S., Li, Y., Vallance, B.A., Vázquez, A., Barba, J., Ibarra, J.A., O'Donnell, P., Metalnikov, P. *et al.* (2004) Dissecting virulence: systematic and functional analyses of a pathogenicity island. *Proc. Natl. Acad. Sci.*, **101**, 3597-3602.

40. Rossi, M.S., Paquelin, A., Ghigo, J.M. and Wandersman, C. (2003) Haemophore-mediated signal transduction across the bacterial cell envelope in *Serratia marcescens*: the inducer and the transported substrate are different molecules. *Mol. Microbiol.*, **48**, 1467-1480.

41. Lesic, B. and Rahme, L.G. (2008) Use of the lambda Red recombinase system to rapidly generate mutants in *Pseudomonas aeruginosa*. *BMC Mol. Biol.*, **9**, 20.

42. Chen, Z., Ling, W. and Shang, G. (2016) Recombineering and I-SceI-mediated *Pseudomonas putida* KT2440 scarless gene deletion. *FEMS Microbiol. Lett.*, **363**.

43. Hossain, M.J., Thurlow, C.M., Sun, D., Nasrin, S. and Liles, M.R. (2015) Genome modifications and cloning using a conjugally transferable recombineering system. *Biotechnol. Rep. (Amst)*, **8**, 24-35.

44. Yamamoto, S., Izumiya, H., Morita, M., Arakawa, E. and Watanabe, H. (2009) Application of lambda Red recombination system to *Vibrio cholerae* genetics: simple methods for inactivation and modification of chromosomal genes. *Gene*, **438**, 57-64.

45. Jia, B., Yang, J.K., Liu, W.S., Li, X. and Yan, Y.J. (2010) Homologous overexpression of a lipase from *Burkholderia cepacia* using the lambda Red recombinase system. *Biotechnol. Lett.*, **32**, 521-526.

46. Kang, Y., Norris, M.H., Wilcox, B.A., Tuanyok, A., Keim, P.S. and Hoang, T.T. (2011) Knockout and pullout recombineering for naturally transformable *Burkholderia thailandensis* and *Burkholderia pseudomallei*. *Nat. Protoc.*, **6**, 1085-1104.

47. Asin-Garcia, E., Garcia-Morales, L., Bartholet, T., Liang, Z., Isaacs, F.J. and Martins Dos Santos, V.A.P. (2023) Metagenomics harvested genus-specific single-stranded DNA-annealing proteins improve and expand recombineering in *Pseudomonas* species. *Nucleic Acids Res.*, **51**, 12522-12536.

48. Hu, S., Fu, J., Huang, F., Ding, X., Stewart, A.F., Xia, L. and Zhang, Y. (2014) Genome engineering of *Agrobacterium tumefaciens* using the lambda Red recombination system. *Appl. Microbiol. Biotechnol.*, **98**, 2165-2172.

49. Khandelwal, R., Agrawal, S., Singhi, D., Srivastava, P. and Bisaria, V.S. (2018) Deletion of pyruvate decarboxylase gene in *Zymomonas mobilis* by recombineering through bacteriophage lambda red genes. *J. Microbiol. Methods*, **151**, 111-117.

50. Corts, A.D., Thomason, L.C., Gill, R.T. and Gralnick, J.A. (2019) Efficient and precise genome editing in *Shewanella* with recombineering and CRISPR/Cas9-mediated counter-selection. *ACS Synth. Biol.*, **8**, 1877-1889.

51. Altschul, S.F., Madden, T.L., Schäffer, A.A., Zhang, J., Zhang, Z., Miller, W. and Lipman, D.J. (1997) Gapped BLAST and PSI-BLAST: a new generation of protein database search programs. *Nucleic Acids Res.*, **25**, 3389-3402.

52. Lu, S., Wang, J., Chitsaz, F., Derbyshire, M.K., Geer, R.C., Gonzales, N.R., Gwadz, M., Hurwitz, D.I., Marchler, G.H., Song, J.S. *et al.* (2020) CDD/SPARCLE: the conserved domain database in 2020. *Nucleic Acids Res.*, **48**, D265-d268.

53. Li, W. and Godzik, A. (2006) Cd-hit: a fast program for clustering and comparing large sets of protein or nucleotide sequences. *Bioinformatics*, **22**, 1658-1659.

54. Mistry, J., Chuguransky, S., Williams, L., Qureshi, M., Salazar, G.A., Sonnhammer, E.L.L., Tosatto, S.C.E., Paladin, L., Raj, S., Richardson, L.J. *et al.* (2021) Pfam: The protein families database in 2021. *Nucleic Acids Res.*, **49**, D412-d419.

55. O'Leary, N.A., Cox, E., Holmes, J.B., Anderson, W.R., Falk, R., Hem, V., Tsuchiya, M.T.N., Schuler, G.D., Zhang, X., Torcivia, J. *et al.* (2024) Exploring and retrieving sequence and metadata for species across the tree of life with NCBI Datasets. *Sci. Data*, **11**, 732.

56. Virtanen, P., Gommers, R., Oliphant, T.E., Haberland, M., Reddy, T., Cournapeau, D., Burovski, E., Peterson, P., Weckesser, W., Bright, J. *et al.* (2020) SciPy 1.0: fundamental algorithms for scientific computing in Python. *Nat. Methods*, **17**, 261-272.

57. Seabold, S. and Perktold, J. (2010), *9th Python in Science Conference*, Austin, pp. 57-61.

58. Sievers, F., Wilm, A., Dineen, D., Gibson, T.J., Karplus, K., Li, W., Lopez, R., McWilliam, H., Remmert, M., Söding, J. *et al.* (2011) Fast, scalable generation of high-quality protein multiple sequence alignments using Clustal Omega. *Mol Syst Biol*, **7**, 539.

59. Katoh, K. and Standley, D.M. (2013) MAFFT multiple sequence alignment software version 7: improvements in performance and usability. *Mol. Biol. Evol.*, **30**, 772-780.

60. Maddison, W.P. and Maddison, D.R. (2023) Mesquite: a modular system for evolutionary analysis. Version 3.81. https://www.mesquiteproject.org. Accessed 20 April 2023.

61. Nguyen, L.T., Schmidt, H.A., von Haeseler, A. and Minh, B.Q. (2015) IQ-TREE: a fast and effective stochastic algorithm for estimating maximum-likelihood phylogenies. *Mol. Biol. Evol.*, **32**, 268-274.

62. Hoang, D.T., Chernomor, O., von Haeseler, A., Minh, B.Q. and Vinh, L.S. (2018) UFBoot2: Improving the ultrafast bootstrap approximation. *Mol. Biol. Evol.*, **35**, 518-522.

63. Kalyaanamoorthy, S., Minh, B.Q., Wong, T.K.F., von Haeseler, A. and Jermiin, L.S. (2017) ModelFinder: fast model

selection for accurate phylogenetic estimates. *Nat. Methods*, **14**, 587-589.

64. Letunic, I. and Bork, P. (2024) Interactive Tree of Life (iTOL) v6: recent updates to the phylogenetic tree display and annotation tool. *Nucleic Acids Res.*, **52**, W78-W82.
